# Applying Ligands Profiling Using Multiple Extended Electron Distribution Based Field Templates and Feature Trees Similarity Searching in the Discovery of New Generation of Urea-Based Antineoplastic Kinase Inhibitors

**DOI:** 10.1371/journal.pone.0049284

**Published:** 2012-11-20

**Authors:** Eman M. Dokla, Amr H. Mahmoud, Mohamed S. A. Elsayed, Ahmed H. El-Khatib, Michael W. Linscheid, Khaled A. Abouzid

**Affiliations:** 1 Department of Pharmaceutical Chemistry, Faculty of Pharmacy, Ain Shams University, Cairo, Egypt; 2 Department of Chemistry, Humboldt-Universität zu Berlin, Berlin, Germany; Wake Forest University, United States of America

## Abstract

This study provides a comprehensive computational procedure for the discovery of novel urea-based antineoplastic kinase inhibitors while focusing on diversification of both chemotype and selectivity pattern. It presents a systematic structural analysis of the different binding motifs of urea-based kinase inhibitors and the corresponding configurations of the kinase enzymes. The computational model depends on simultaneous application of two protocols. The first protocol applies multiple consecutive validated virtual screening filters including SMARTS, support vector-machine model (ROC = 0.98), Bayesian model (ROC = 0.86) and structure-based pharmacophore filters based on urea-based kinase inhibitors complexes retrieved from literature. This is followed by hits profiling against different extended electron distribution (XED) based field templates representing different kinase targets. The second protocol enables cancericidal activity verification by using the algorithm of feature trees (Ftrees) similarity searching against NCI database. Being a proof-of-concept study, this combined procedure was experimentally validated by its utilization in developing a novel series of urea-based derivatives of strong anticancer activity. This new series is based on 3-benzylbenzo[d]thiazol-2(3H)-one scaffold which has interesting chemical feasibility and wide diversification capability. Antineoplastic activity of this series was assayed in vitro against NCI 60 tumor-cell lines showing very strong inhibition of GI_50_ as low as 0.9 uM. Additionally, its mechanism was unleashed using KINEX™ protein kinase microarray-based small molecule inhibitor profiling platform and cell cycle analysis showing a peculiar selectivity pattern against Zap70, c-src, Mink1, csk and MeKK2 kinases. Interestingly, it showed activity on syk kinase confirming the recent studies finding of the high activity of diphenyl urea containing compounds against this kinase. Allover, the new series, which is based on a new kinase scaffold with interesting chemical diversification capabilities, showed that it exhibits its “emergent” properties by perturbing multiple unexplored kinase pathways.

## Introduction

**Figure 1:**
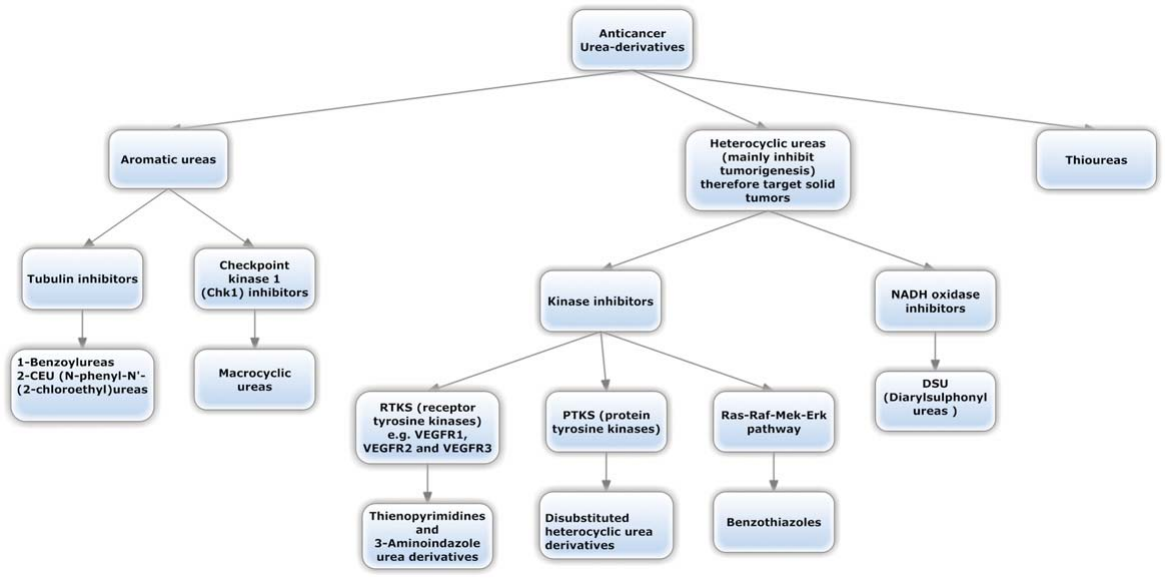
Classification of urea-based antineoplastic kinase inhibitors according to the general chemical structure and highlighting the general mechanism.

Within the past years, a huge number of researches on the synthesis, structure-activity relationships (SAR) and the anticancer activities of the urea derivatives were reported [1]. According to the review done by Li et al [1], they were classified into three groups: aromatic, heterocyclic and thioureas. The classification was done on a chemical structure basis which we summarized and additionally included the mechanistic action (Figure 1).

It is obvious from this classification that many anticancer heterocyclic urea derivatives act as kinase inhibitors [2], [3]. Bearing this fact in mind, we decided accordingly to explore this branch and tried to develop a computational protocol which can lead to the discovery of new generations of kinase inhibitors with cancericidal activity based on new heterocyclic urea derivatives. One important aspect which was of primary concern here was to achieve novelty in the discovered structures such that they have a different selectivity profile against kinome by applying the concept of fuzziness and remote hopping in compounds screening using Cresset Field technology. We didn't restrict choice on those compounds that are merely selective on a specific kinase as this is practically very difficult. Additionally, this didn't deter the development of clinically significant kinase inhibitors and the evidence is that most approved kinase inhibitors have limited selectivity and target kinases [4]–[6]. This is with the exception of the highly selective inhibitor lapatinib [7].Restricting choice on highly selective compounds actually is very difficult if we take into consideration a large part of the kinome panel due to the high similarity of the binding site among different kinases. It is of course preferable that we find a highly selective inhibitor, but we didn't let such restriction prevent us from choosing compounds that show selectivity against different kinases while showing anticancer activity hoping that it might be clinically safe.

## Design Process

This study can be divided into several parts:

First: Developing a novel computational procedure that allows screening of urea derivatives that can act as kinase inhibitors.

Second: Developing another computational procedure that allows verification of cancericidal activity of the hits in order to prioritize selection.

Third: Experimental verification through in-vitro cytotoxicity assay using human tumor cell lines for general anticancer activity and high throughput kinase profiling for mechanistic action exploration.

**Figure 2:**
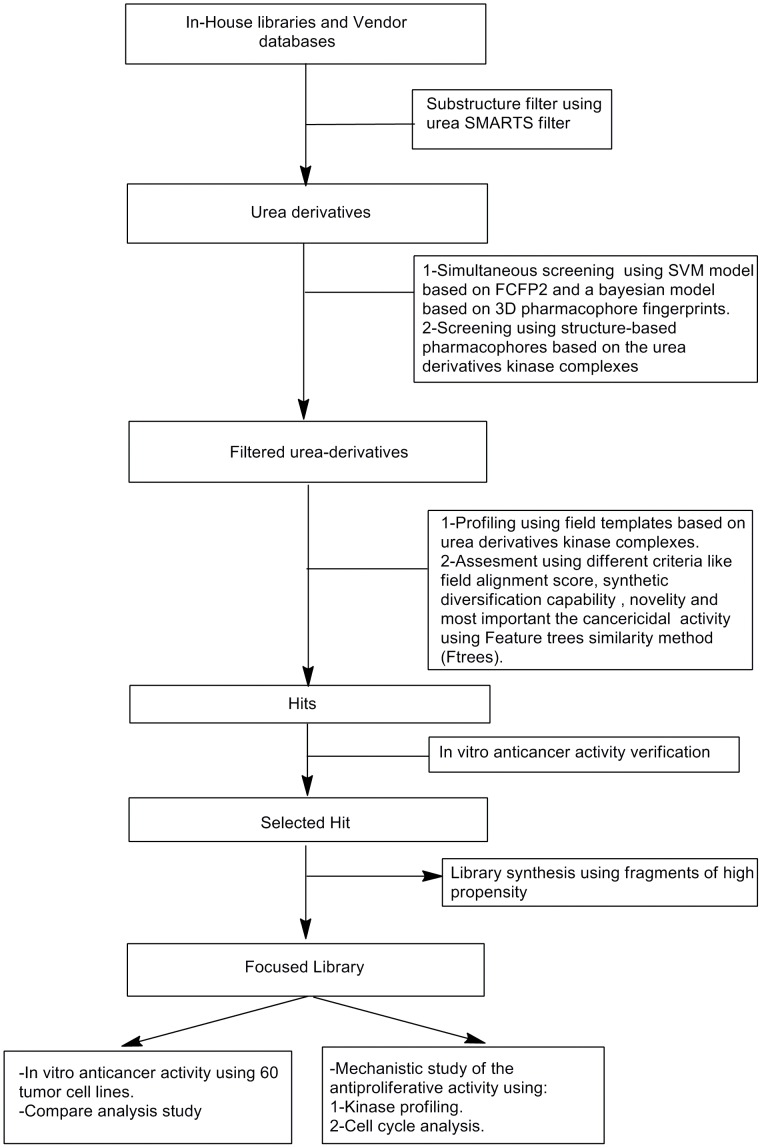
General workflow of the study which includes the computational procedure of ligand profiling using multiple field templates, the protocol of cancericidal verification using features similarity method, the in vitro cytotoxicity assays and finally the mechanistic study using high-throughput kinase profiling and cell cycle analysis.

The general workflow of the study was summarized in Figure 2.

## Results and Discussion

### Molecular modeling

#### Profiling of heterocyclic-urea derivatives against kinases

The first step in the molecular modeling was to develop a procedure that allows screening of urea derivatives against kinases. One approach is to use a general pharmacophore for kinase inhibitors [8] to screen urea derivatives. However, this approach neglects all the cumulative literature data regarding these types of inhibitors and thus lengthens the discovery pathway by including avoidable false positives. This problem was solved easily by deploying a knowledge-based strategy as will be described.

We decided to screen urea-based derivatives by applying consecutive filters followed by profiling against a panel of kinases with available structural-data (urea-based inhibitor-kinase complexes) using an array of field templates [9] created using these complexes. Field templates are types of pharmacophores that are based on field points instead of conventional pharmacophore features (H-bond donor, H-bond acceptor etc.). This field technology was developed by Cresset BioMolecular Discovery. It relies on field that is defined by a new force field called the eXtended Electron Distribution (XED. [10], [11]. These fields encode information about electrophilicity, electrophobicity (nucleophilicity), van der Waals attraction (referred to as ‘sticky’ points), and hydrophobicity. The field templates were generated using Field align 2.1 (Cresset BioMolecular Discovery, Hertfordshire, UK).

Field templates were selected as the final virtual screening element for many reasons:

1. It takes into consideration the structural data available which includes:
  - 3D conformations of the bioactive conformers of the inhibitors.
  - The binding mode of the urea-based inhibitors in the binding site which vary considerably. This can be achieved by constraining urea-fragments field points in the different regions it can act on.
  - The different configuration patterns of kinase enzymes.
2. It depends on the field perception of various inhibitors and not on geometrical features. This includes the electrostatic and van der Waal properties thus describing what the receptor actually “sees” in terms of charge distribution and shape rather than merely focusing on the underlying structural skeleton. This has an additional advantage of achieving novelty as this can lead to a remote shift in the structures discovered. Here in this study, we used field align that make use of the extended electron distribution force field to describe the charge distribution.
3. It is highly flexible and allows selection according to complex criteria which can include:
  - alignment scores against the field templates
  - The general profile attained against various kinases by heat map inspection

These criteria can be considered together with others like novelty, synthetic feasibility and most important the cancericidal activity.

The computational procedure described above was carried out on many steps:

#### Retrieval of urea-based kinase inhibitors complexes from the PDB

**Figure 3:**
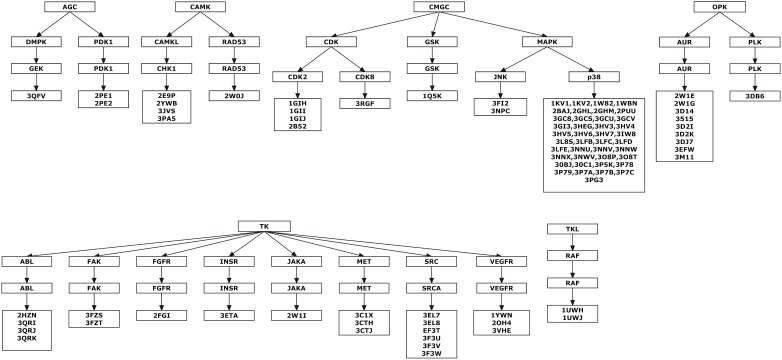
Classification of urea derivatives kinases complexes deposited in literature according to their families, subfamilies and groups and listing the PDB codes of each group.

Urea derivatives kinases complexes were retrieved and classified according to the kinome groups, subgroups and families. We listed the pdb complexes under each family as shown in Figure 3.

#### Analyzing the complexes binding motifs and creating field templates

**Figure 4:**
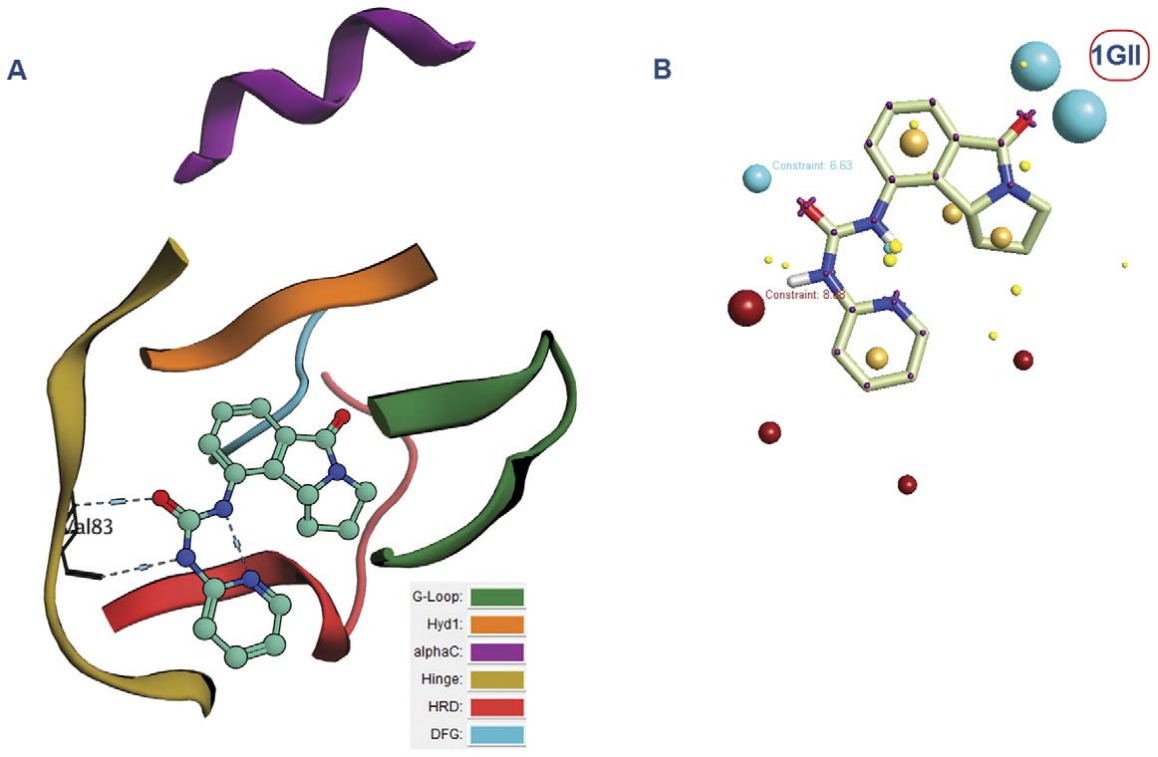
Human cyclin dependent kinase 2 complexed with urea-based cdk4 kinase inhibitor (1GII): (A)The complex illustrated using the color codes that represent the different regions of the binding site: G-loop, Hyd1, alphaC, Hinge, HRD and DFG regions. The urea fragment binds to the Hinge region, (B) The corresponding field template derived from the complex. Color codes of the field template are listed in the supplementary data (Text S2).

**Figure 5:**
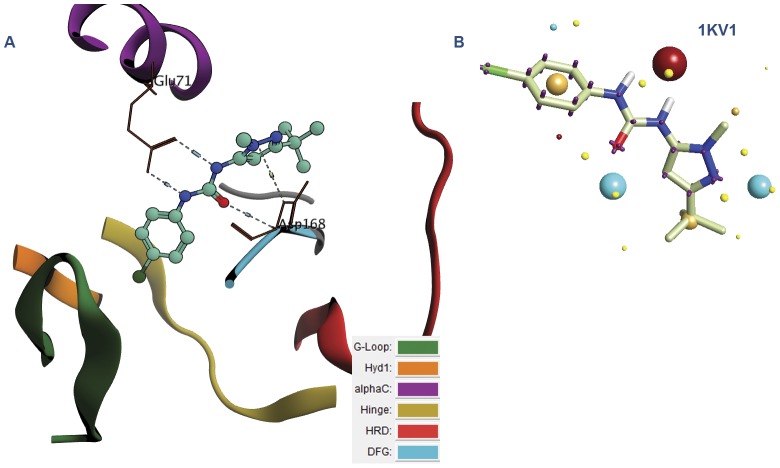
P38 MAP Kinase in Complex with urea-based inhibitor (1KV1): (A) The color codes represent the different regions of the binding site: G-loop, Hyd1, alphaC, Hinge, HRD and DFG regions. The urea fragment binds to the DFG and alphaC regions. (B) The corresponding field template derived from the complex. Color codes of the field template are listed in the supplementary data (Text S2).

The first thing done with these retrieved complexes was the analysis of the binding mode adopted by different inhibitors. This was carried out by classifying the binding site into several regions: G-loop,Hyd1, alphaC, Hinge, HRD and DFG regions [12] where the secondary structure was color coded according to these regions to allow rapid analysis (see Figure 4 and Figure 5). According to the detailed analysis (see Text S1) of the binding site, it was generally deduced that the urea moiety can bind either to DFG region, the Hinge region or Hyd1 region. This affects the type according to which the inhibitor can be classified (type I or type II) [13]. Besides, this analysis allowed the inspection of different configurations of the kinases whether they are DFG-in or out and if the alphaC, which deals with the highly conserved Lys72 with respect of Glu91 in the center of αC-helix, is in or out. In the *in* conformation, Glu91 forms an ionic interaction with Lys72. The analysis was clarified by including the Pymol session files of the retrieved complexes (see File S1). Each file focuses on the binding site region and incorporates the secondary structures color coded according to the different regions to make it easy to detect the region where the urea moiety binds. The files can be opened using Pymol v1.3 and above (Schrödinger, LLC, Portland, USA).

Practically, the urea derivatives were used to generate the field templates (or field pharmacophore) while the kinases (proteins) were used as excluded volumes. An additional criterion was used in which the urea was constrained in all these templates to maintain the positional aspect in the different binding motifs (see Figure 4 and Figure 5). It is should be noted that field templates are color coded. These color codes are explained in the supplementary data (see Text S2). Summing up, we used the retrieved panel of kinases complexes in order to encompass all the available structural data of urea-based kinase inhibitors, different kinases configurations and different binding motifs in the ligand profiling process while attaining fuzziness through the usage of field technology.

#### Screening Vendor databases and in-house libraries using rapid consecutive virtual screening filters

Vendor databases like Chemdiv were selected as one of the world's largest collection of small molecules for various applications. Additionally, other databases which were supplied with MOE package were included together with our in-house libraries (MOE version 2010.10 The Molecular Operating Environment, Chemical Computing Group Inc., Montreal, Canada).

The compound libraries were virtually screened using a set of consecutive filters before they were profiled against the field templates.

First, urea-based compounds were extracted from the databases using a simple substructure searching which depends on SMILES Arbitrary Target Specification (SMARTS) pattern implemented in Accelrys Discovery studio v 3.0 (Accelrys Software Inc., San Diego, CA, USA) and Schrodinger Canvas 1.5 (Schrödinger, LLC, Portland, USA).

**Figure 6:**
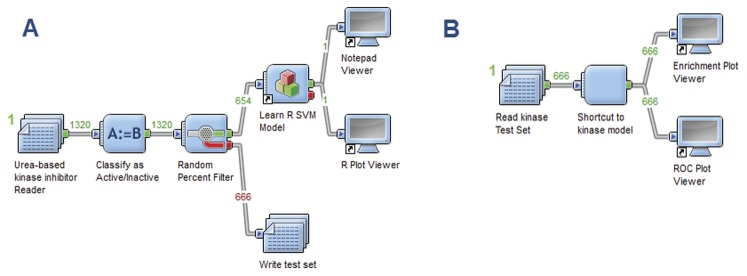
Pipeline pilot workflow used to carry out the SVM model using R statistics package. (A) Shows the usage of R-statistics node in pipeline pilot and its usage in learning the training set, after splitting, followed by giving the cross-validated ROC score via R plot viewer. (B) Shows the usage of the test set to validate the model using enrichment plot and R plot viewer.

Second, the retrieved compounds with urea fragments were screened simultaneously using two filters based on two models. These models were developed and validated in our lab. The first model is a support vector machine [14] which was constructed using a SciTegic 2D-fingerprint descriptor (ECFP_4) as the independent property to learn from. This model was used as it will likely enrich the results with hits that have fragments highly available in the known references. It was carried out using a simple Pipeline pilot workflow as depicted in Figure 6. The Accelrys Pipeline Pilot 8.0 was used (Accelrys Software Inc., San Diego, CA, USA). The second model is based on Bayesian categorization that uses 3-point feature pharmacophore 3D fingerprints as the independent property. This was used as it can enrich results with hits that are structurally different from the references yet having the same pharmacophore pattern. In other words, it was used to ensure retrieval of hits with different Chemotype. The two models were constructed and validated both internally using 5-fold cross validation and externally using an enrichment plot, ROC plot, and computing an overall ROC score as described in the supplementary data (see Text S3 and Text S4). After that, the hits retrieved were merged and duplicates were removed.

**Figure 7:**
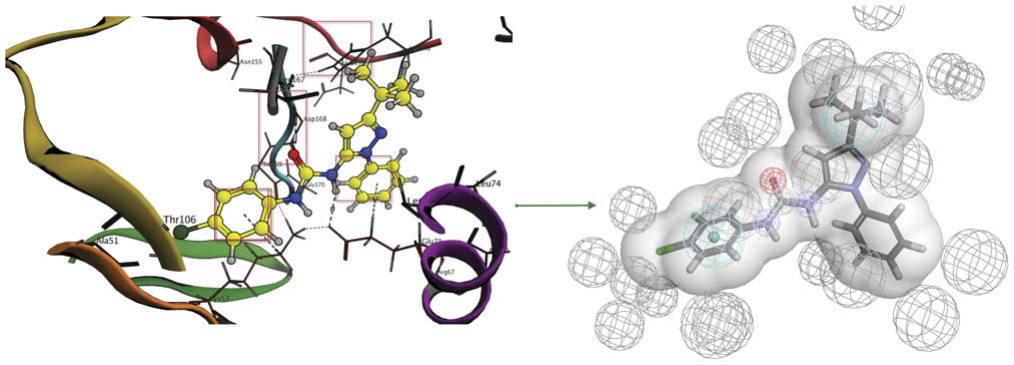
Structure-based pharmacophore construction general outline. The interactions are translated into pharmacophore features while the binding site amino acids are translated into excluded volumes. The shape of the binding site is also added to increase sensitivity of the pharmacophore.

Third, the hits were further filtered using structure-based pharmacophores that represent the different configurations and binding motifs of the urea-based kinase inhibitors. These structure-based pharmacophores were constructed according to the following steps:

1-Complexes were categorized according to the urea-binding region (DFG or Hinge or HRD region) in each kinase group (AGC, CAMK, CMGC, OPK, TK and TKL).
2- Structure-based pharmacophores were created for the complexes in each category using Wolber technique [15] and modified by adding a custom feature for urea fragment in order to ensure the correct positioning of this specific fragment in the binding site. The features in this technique are perceived from the observed interactions and excluded volumes are added according to the binding site amino acids (Figure 7). This technique was carried out by the structure-based pharmacophore protocol implemented in Accelrys Discovery studio 3.0 (Accelrys Software Inc., San Diego, CA, USA).
3-Pharmacophores in each category were then clustered and cluster centers were only kept.

The pharmacophores retrieved for the kinases complexes are given in details in the supplementary data (Text S5).

Theses structure-based pharmacophores serve very important functions which can be summarized as following:

1-General rapid pre-filtering for ligands that takes important features in consideration and this is the general function for any pharmacophore.
2-Proper positioning of the ligands in the binding sites by finding the proper conformations which can map properly with the pharmacophore features inside these binding sites while avoiding bumping with the excluded volumes that represent the binding sites amino acids. This is specific feature for structure-based pharmacophores where it can act as alternative to docking [15].

In spite of these advantages, the pharmacophores extracted are not sensitive enough to show selectivity against a specific kinase but allover they can retrieve all the inhibitors extracted from the complexes. In other words, they can't be used in kinase profiling. This is mainly because kinases generally share the same binding site features where slight differences between ligands can't be perceived using general geometrical features. That is why we followed it by field templates ligand profiling as it considers the detailed electrostatic and steric map of each ligand while comparing it to the reference and thus gives a more precise selectivity.

#### Profiling the retrieved compounds by field aligning against the generated array of field templates

**Figure 8:**
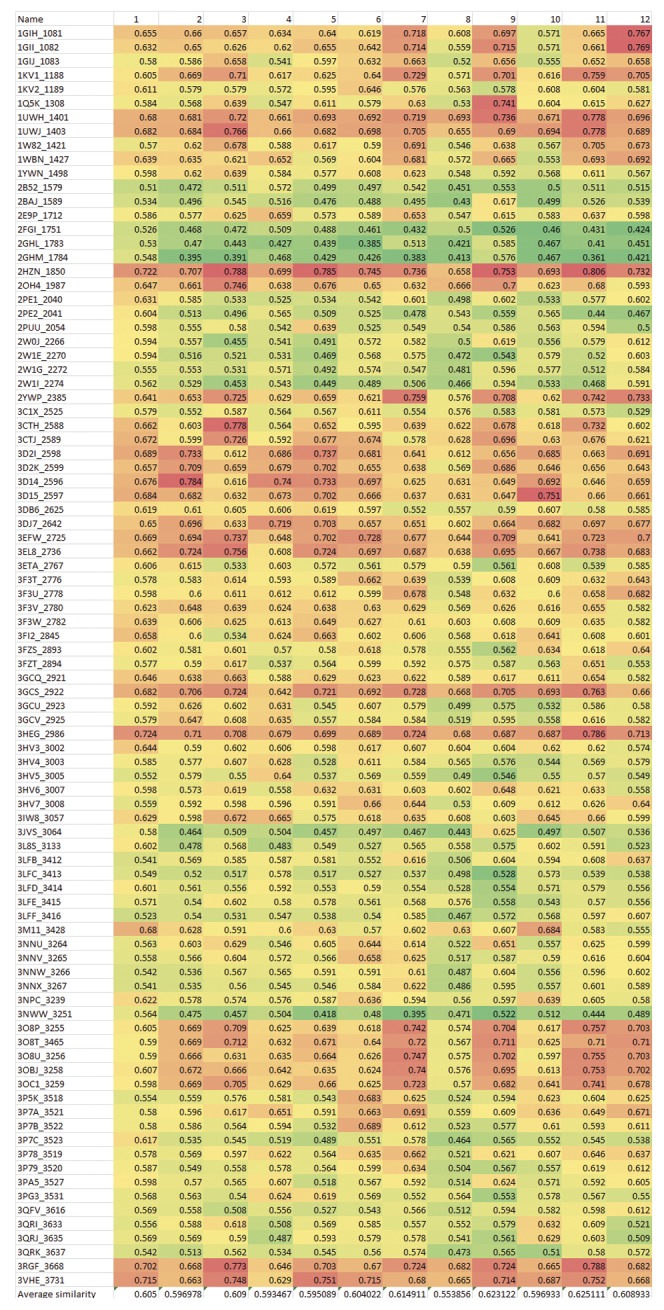
Heat map of twelve urea-based derivatives against a panel of 90 field templates representing urea –based kinases inhibitors complexed with their corresponding kinase enzymes as retrieved previously. The color codes used here is the red-yellow-green scale as indicative for decreasing similarity.

**Figure 9:**
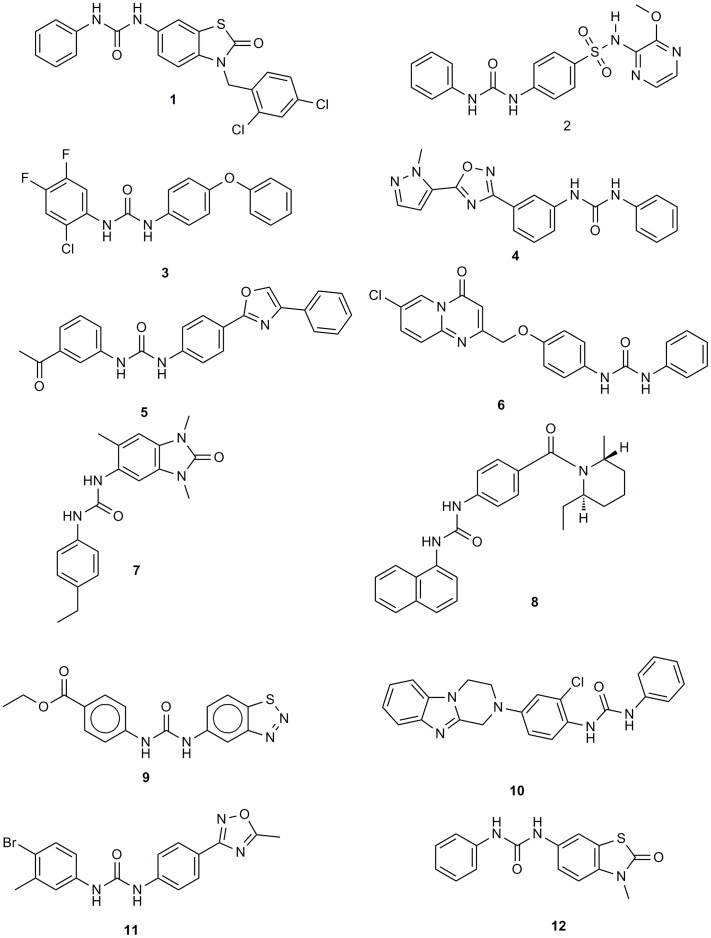
Structures of the hits mentioned in **figure 8** as an example of the screening protocol results.

Finally the hits were profiled against the generated field templates and scored using the alignment score included in Field Align 2.1 (Cresset BioMolecular Discovery, Hertfordshire, UK). In order to demonstrate the results of alignment, we gave an excerpt of the hits alignment scores in Figure 8. Those hits are given in Figure 9.

#### Developing a computational procedure that allows verification of the cancericidal activity

After the urea derivatives were profiled against the field templates, selection was carried out. Selection criteria, which we focused on here, were mainly the alignment score, novelty and most important is the likelihood of being anticancer which required us to develop a computational procedure to be able to evaluate this possibility. This is because not all kinase inhibition can be translated into cancericidal activity. We have limited knowledge regarding this interpretation as only few kinase targets reported in literature were clinically approved if compared to the larger percentage of untargeted cancer kinome [16].

In other words, the kinase inhibition profile, especially if different from the common patterns of known inhibitors, can't be translated into a probable cancericidal activity. Therefore, we developed a method which can check this.

It is also important to note that one can restrict the choice on ligands with high similarity to known anticancer references or not as the advantage of using field similarity lies mainly in the finding highly remote structures that don't share the same common skeleton and thus will likely have a different activity pattern.

In an attempt to verify computationally, the cancericidal activity of our hits, we decided to carry out similarity searching of the hits against NCI compounds with reported anticancer activities. This will give us a close picture of similar compounds to those of our hits if there already exist and thus prioritizing hits selection, thereby adding an important factor to be considered while profiling ligands against kinases. One can verify this method easily by comparing the biological pattern of the hits against those of the similar compounds retrieved from NCI.

Herein, we have chosen a fast method for similarity searching that depends on non-linear descriptors (feature-tree descriptors) that can capture key properties of the compound. The Feature Tree descriptor represents the molecule as an unrooted tree where the nodes of the tree describe the major building blocks of the molecule. The comparison of two Feature Trees is based on a recursive matching algorithm, splitting the trees into smaller and smaller subtrees. The Feature Tree approach has several advantages, the most important being the fact that the alignment of two Feature Trees can be translated into a comprehensible mapping of the two underlying molecules [17].

#### Selection and experimental verification

Allover, the workflow was implemented as shown in the supplementary data (see Text S6). In virtual screening studies, normally hits found (Figure 9 and Text S6) are diverse having different scaffolds where each hit can be considered as a separate template for developing SAR analysis and optimization studies.

We decided here to exploit one of the template hits rather than discussing diverse solutions. In our opinion, this was important because we wanted to address the importance of using fragments of high propensity in kinase inhibitors in developing derivatives.

**Figure 10:**
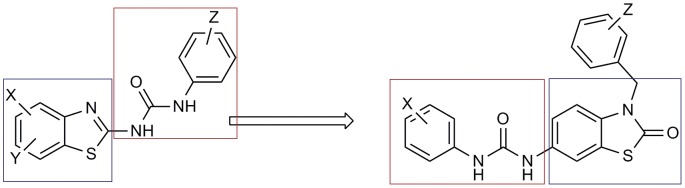
Scaffold morphing observed in the hit was used as one of the selection criteria. The diversification is achieved by having different attachment points and thus different geometrical diversity in the virtual space of its substituents.

Virtual screening hit 1 (Figure 9) was selected based on many aspects:

1-Average similarity scores across the panel of 90 kinases were used as a preliminary filter. We kept only compounds with average similarity above 0.6. This was based on a validation study which proved the better enrichment of urea-based kinase inhibitors recovered at the top 10% when the average similarity score is set to above 0.6.
  We should point out here that the most important aspect of this method is the flexibility in choosing the hits. For instance, we were concerned here with the antineoplastic activity of the hits so we set the second filter according to the similarity score of the hit to the clinically validated antineoplastic drug Sorafenib. We ranked the filtered hits according to their similarity score to this drug.
  In order to demonstrate the process of selection, we gave an excerpt of the hits in Figure 8. The average similarity is given for each hit in the last raw of the heat map. Sorafenib pdb code is **3HEG** where compound **1** (later **12a** according to the synthetic scheme) was selected according to the rank of its similarity.
2-The high propensity of thiazole [18], [19] (and benzothiazole [20]) moiety among urea-based kinase inhibitors and especially those of anticancer activity.
3-The diversification and the scaffold morphing by drifting from the substituted 2-amino thiazole pattern of inhibitors to thiazol-2(3H)-one (see Figure 10). The diversification is achieved by having different attachment points and thus different geometrical diversity in the virtual space of its substituents. The versatility also is achieved through the synthetic feasibility of snapping different substituents to the different attachment points thus creating a wide range of possible derivatives through combinatorial chemistry.
4-The results of FTrees similarity searching against NCI which showed many ligands with high similarity to the chosen ligand. Example of the hits retrieved using Ftrees similarity searching is mentioned in the supplementary data (Text S7).

The cytotoxicity of the selected virtual screening hit was tested after it has been synthesized (see next section) against Huh-7 colorectal adenocarcinoma as a preliminary test to verify the anticancer activity (It showed IC50 of 2.8 uM) (Table 1). Based on the results, we developed a series of compounds using the same template while varying the substituents. The diversification was intended to explore different kinase targeted fragments like haloarens and thiourea. Haloarenes represent the hydrophobic feature in the general kinase pharmacophore and was varied to check its effect on the series [8]. Thiourea is a main class of anticancer urea-derivatives and was also checked for its effect [1].

**Table 1:** IC_50_ of the synthesized compounds on Huh-7 colorectal carcinoma cell line.

Compound	IC50 (µm)
**12a**	2.8
**12b**	2.2
**12c**	1.7
**12d**	0.4
**12e**	0.8
**12f**	0.3
**12g**	0.8
**12h**	0.7
**12i**	NT
**12j**	NT
**12k**	1.0
**12l**	1.6
**13**	0.3

### Synthesis

**Figure 11:**
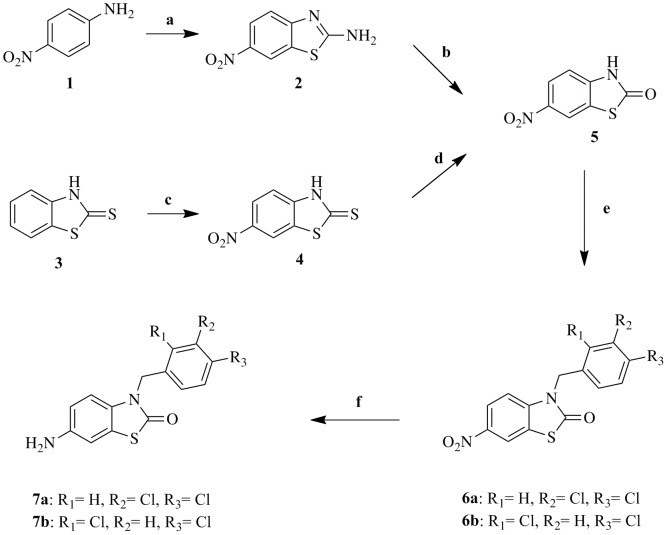
Synthesis of intermediates 7a–b. Reagents and conditions: (a) Br_2_, NH_4_SCN, AcOH, below 10°C, 3 h; (b) NaNO_2_, H_2_SO_4_, below 10°C for 15 min, then at r.t. for 1 h ; (c) H_2_SO_4_, NaNO_3_, 0°C, 1 h; (d) KMnO_4_ (10%), NaOH (25%), 80–90°C, 30 min; (e) Acetone, KOH (85%), H_2_O, ArCH_2_Cl, Reflux, 24 h; (f) H_2_/10%Pd/C, EtOH/THF (3∶1), 35 psi, r.t., 6 h.

**Figure 12:**
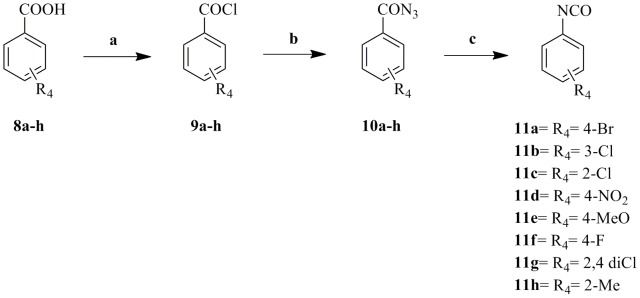
Synthesis of intermediates 11a–h. Reagents and conditions: (a) SOCl_2_, reflux, 5 h; (b) NaN_3_, acetone, −10°C, 30 min; (c) Benzene, 70°C, 3 hrs.

**Figure 13:**
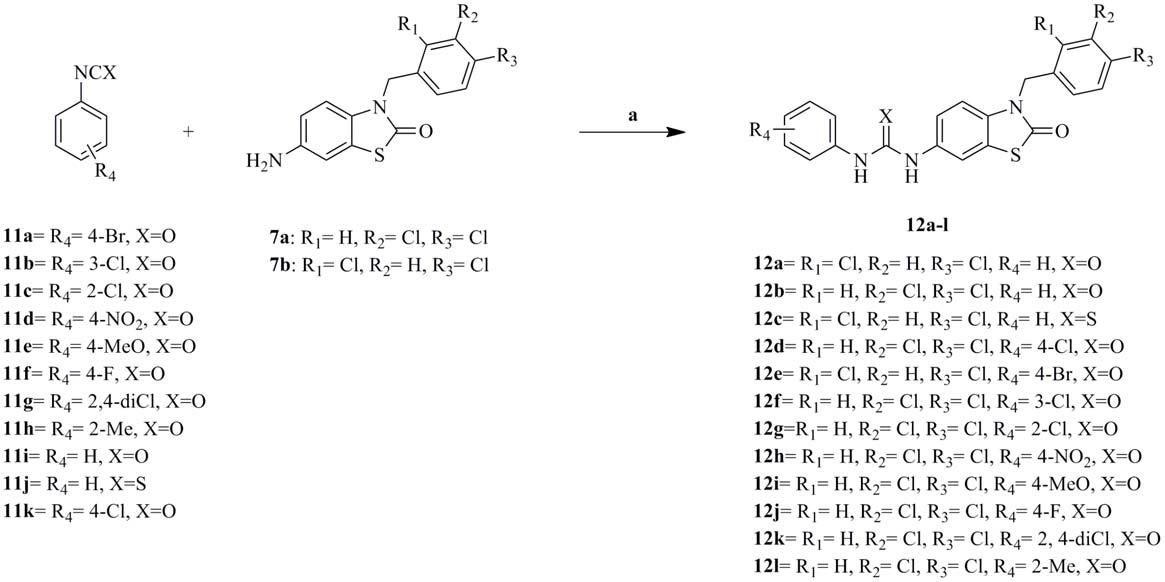
Synthesis of target compounds (12a–l). Reagents and conditions: (a) CH_2_Cl_2_, r.t, overnight.

**Figure 14:**
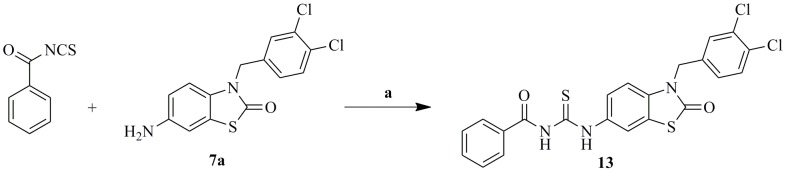
Synthesis of target compound (13). Reagents and conditions: (a) CH_2_Cl_2_, r.t, overnight.

The synthetic approaches adopted to obtain the different derivatives (**12a–l, 13**) are outlined in Figure 11, Figure 12, Figure 13 and Figure 14.

Two synthetic routes were attempted to synthesize 6-nitro-2, 3-dihydrobenzo[d]thiazol-2-one (5). Method (A) started with p-nitroaniline cyclization to 6-nitro-2-amino-2, 3-dihydrobenzo[d]thiazole (**2**) under standard conditions [21], then diazotization and hydrolysis of (**2**) in acidic medium [22], to give the intermediate (**5**) in an overall low yield (26%). In method (B) we started with 2-mercapto-2, 3-dihydrobenzo[d]thiazole (**3**) where nitration using sodium nitrate in sulfuric acid gave the nitro derivative (**4**) in high yield [23], then oxidation of (**4**) using potassium chromate (10%) in basic medium [24] gave the intermediate (**5**) in an overall moderate yield (56%).

Alkylation of (**5**) to give the corresponding unreported N-alkylated intermediates (**6a–b**) was attempted in KOH/acetone/water mixture using appropriate aralkyl halide under reflux conditions [25] to obtain the desired intermediates (**6a–b**) in an excellent yield.

Reduction of the N-alkylated intermediates (**6a–b**) was carried under standard conditions reported by Abdelaal et al [22] using 10%Pd/C and hydrogenation at 35 psi to give the corresponding amine (**7a–b**). Carrying the reduction in ethanol/THF in a ratio (3∶1) improved the yield dramatically.

The aryl isocyanates intermediates (**11a–h**) were prepared according to [scheme 2] starting from the corresponding acids (**8a–h**), converting them to the acid chloride (**9a–h**) using thionyl chloride under reflux conditions [26], then reacting the appropriate acid chloride with sodium azide in acetone for 30 min at 0°C to give the corresponding aryl azides (**10a–h**) [27], finally Curtis rearrangement of the aryl azides (**10a–h**) to the corresponding aryl isocyanates (**11a–h**) was achieved by heating the aryl azides for 3 hours at 70°C in dry benzene [28].

The amine (**7a–b**) was reacted with the appropriate aryl isocyanates or isothiocyanate (**11a–k**) or benzoyl isothiocyanate in methylene chloride to give the target products (**12a–l, 13**) in moderate to high yields.

### Biological activity

#### In vitro antiproliferative activity

Cytotoxicity of these derivatives was preliminarily tested just like 12a on Huh-7 colorectal cells (see Table 1). Based on the promising results of this preliminary test, we submitted the compounds to be evaluated in the full panel of 60 different human tumor cell lines, representing leukemia, melanoma and cancers of the lung, colon, brain, ovary, breast, prostate, and kidney which represent the panel of cell lines of national cancer institute NCI. Six of the newly synthesized compounds (12b, 12d, 12e, 12i, 12j, 12k) were selected for the first stage of the NIH screening in which the compounds were evaluated against the 60 cell lines at a single dose of 10 uM (see Table 2).

**Table 2:** Inhibition percentage of compounds (12b, 12d, 12e, 12i, 12j and 12k) at 10 µm dose in the full panel of 60 cell line.

Cell Name	12b	12d	12e	12i	12j	12k
***Leukemia***						
CCRF-CEM	25.62	97.30	72.58	27.55	3.32	87.78
HL-60(TB)	4.14	139.09	80.16	0.26	−7.98	108.27
MOLT-4	3.15	85.75	51.77	22.67	5.14	78.95
RPMI-8226	40.69	116.32	98.31	47.69	25.84	118.61
SR	−2.72	94.05	82.56	10.19	−10.98	100.76
***Non-Small Cell Lung Cancer***						
A549/ATCC	39.42	110.95	58.95	20.85	19.07	89.00
EKVX	47.34	93.22	47.33	32.60	21.35	63.16
HOP-62	49.87	104.50	54.23	−6.36	0.07	85.57
HOP-92	7.32	138.96	110.20	5.75	NT	119.32
NCI-H226	62.11	93.06	30.40	15.79	14.74	57.02
NCI-H23	39.95	116.40	78.29	14.77	10.14	92.60
NCI-H322M	39.09	78.33	28.74	21.43	27.24	37.54
NCI-H460	25.99	122.28	82.03	17.36	1.55	99.13
NCI-H522	14.36	109.55	55.20	19.64	−5.43	70.02
***Colon Cancer***						
COLO 205	81.08	197.92	69.49	−18.61	−22.93	128.13
HCC-2998	32.04	99.49	77.86	6.65	2.49	87.91
HCT-116	50.66	166.38	91.05	22.24	19.83	146.55
HCT-15	45.85	99.92	82.60	16.16	19.33	90.97
HT29	1.23	114.35	65.60	14.06	−19.41	89.25
KM12	24.22	98.13	78.58	9.91	13.72	85.95
SW-620	8.60	95.33	78.91	4.79	−8.92	93.02
***CNS Cancer***						
SF-268	2.18	64.44	27.82	1.36	4.81	30.17
SF-295	61.41	134.97	94.00	40.50	27.96	98.76
SF-539	13.19	79.95	13.03	6.61	−8.27	46.15
SNB-19	67.30	92.94	58.83	2.02	−3.76	62.84
SNB-75	17.29	99.50	55.73	7.18	13.70	62.63
U251	25.57	133.06	65.56	6.64	10.51	82.85
***Melanoma***						
LOX IMVI	31.17	106.54	55.32	15.45	4.15	77.24
MALME-3M	9.89	118.67	56.59	17.35	−2.63	99.82
M14	24.99	110.05	67.77	14.31	0.65	80.86
MDA-MB-435	14.52	101.91	48.06	13.80	1.85	80.20
SK-MEL-2	9.33	121.49	57.92	3.50	−21.30	47.68
SK-MEL-28	21.39	75.59	41.31	7.19	−0.90	38.23
SK-MEL-5	73.50	178.21	36.60	26.36	16.85	70.54
UACC-257	8.29	128.05	42.91	4.61	14.35	44.50
UACC-62	86.54	93.77	60.95	32.34	20.93	72.78
***Ovarian Cancer***						
IGROV1	1.11	58.62	52.92	−5.11	−13.61	68.55
OVCAR-3	17.62	116.13	104.38	21.96	19.88	129.03
OVCAR-5	20.82	68.41	25.58	0.76	−0.46	54.47
OVCAR-8	24.46	96.71	41.99	6.92	−3.55	54.73
NCI/ADR-RES	54.22	98.76	58.51	20.38	12.70	70.98
SK-OV-3	16.44	98.07	14.33	−6.44	8.05	44.85
***Renal Cancer***						
786-0	21.87	94.48	66.53	19.33	12.15	84.14
A498	30.76	106.89	39.58	11.66	5.40	88.82
ACHN	66.67	122.68	90.22	18.40	−11.85	110.28
CAKI-1	53.53	101.39	79.65	28.57	19.66	68.83
RXF 393	28.98	95.97	25.60	4.74	4.73	83.62
SN12C	71.74	95.41	58.57	9.51	9.54	56.15
TK-10	14.70	80.78	26.30	7.87	−20.30	52.84
UO-31	25.05	96.84	35.50	19.51	10.87	41.51
***Prostate Cancer***						
PC-3	25.09	121.66	82.58	22.25	8.73	96.33
DU-145	−4.15	90.01	65.10	8.33	−0.06	78.82
***Breast Cancer***						
MCF7	53.92	94.32	74.61	21.85	17.87	90.34
MDA-MB-231/ATCC	56.91	97.80	52.27	27.72	17.85	57.78
HS 578T	25.51	96.72	39.65	−3.58	−2.41	28.72
BT-549	13.38	92.21	33.89	12.03	3.73	19.80
T-47D	86.74	104.46	86.72	26.45	21.72	93.75
MDA-MB-468	77.45	123.31	94.28	26.19	31.75	118.21

The mean inhibition percentages of all of the tested compounds over the full panel of cell-lines are shown in Table 3. It is obvious that the tested compounds have expressed weak (**12i,12j**) to moderate (**12b,12e,12k**) mean inhibition over the whole cell-lines panel except for compound **12d**, where a great mean inhibition of 106.24%was observed over the cancer cell-lines, indicating that the compound effect has exceeded the inhibitory limit (100% inhibition) to the lethal effect (regression of tumor size from the original size at the beginning of the experiment) at the test dose (10 uM). The multiple inhibitions of compounds (**12b**, **12d**, **12e**, **12k**) over the 60 cell-lines are illustrated in the supplementary data (Text S8). The inhibitory effect of the compound **12d** approaches the limit of 200% (100% lethality or complete tumor regression) in some of the cell lines at the test dose. The inhibitory effect is very strong over almost all of the 60 cell-lines, with a significant lethality at some of colon and melanoma cell-lines. The main observation regarding the activity is that highest potency is related to the halogen substitution at the urea phenyl ring.

**Table 3:** Mean growth inhibition of 6 compounds at 10 µm dose (12b, 12d, 12e, 12i, 12j and 12k) while highlighting the selected ones for further IC_50_ evaluation.

Compound	12b	12d	12e	12i	12j	12k
Mean growth inhibition	32.58%	106.24	60.45%	13.86%	5.96%	77.88%

According to these results, 4 compounds (**12b, 12d, 12e, 12k**) which exhibit significant growth inhibition were evaluated against the panel of 60tumor cell lines at five concentration levels. Three dose response parameters are calculated for each experimental agent. Growth inhibition of 50% (GI_50_), total growth inhibition (TGI) and the 50% lethal concentration (LC_50_) (see Table 4). All compounds tested exhibited NCI-60 mean GI_50_ lower than 5 uM. Compounds **12d**, **12e** and **12k** exhibited cytotoxic effect at higher doses displaying selectivity towards leukemia, CNS cancer, renal cancer and prostate cancer.

**Table 4:** GI_50_, TGI and LD_50_ values mean and Full Panel GI_50_ Mean Graph Mid-Point (MG-MID) for the selected four compounds (12b, 12d, 12e and 12k).

	12b			12d			12e			12k		
Cell Name	GI50	TGI	LC50	GI50	TGI	LC50	GI50	TGI	LC50	GI50	TGI	LC50
***Leukemia***												
CCRF-CEM	3.7	17.0	>100	2.1	4.5	9.3	2.5	34.7	69.2	1.6	3.3	6.9
HL-60(TB)	3.9	100.0	>100	1.1	10.5	53.7	2.3	>100	53.7	1.7	4.0	9.5
MOLT-4	3.9	>100	>100	2.8	16.6	>100	3.3	22.9	67.6	3.3	14.1	46.8
RPMI-8226	2.8	8.7	>100	0.9	4.3	43.7	1.4	15.5	89.1	1.3	3.5	9.8
SR	13.2	>100	>100	3.5	12.0	87.1	3.6	72.4	60.3	2.4	5.9	25.7
mean	5.5	>100	>100	2.1	9.6	>100	2.6	>100	**68.0**	2.1	6.2	**19.7**
***Non-Small Cell Lung Cancer***												
A549/ATCC	3.5	15.8	66.1	1.7	6.5	25.1	2.8	>100	46.8	1.7	3.5	7.2
EKVX	3.5	29.5	>100	1.9	14.5	>100	2.4	>100	77.6	2.0	10.0	>100
HOP-62	7.4	21.4	51.3	3.3	11.7	49.0	5.8	>100	74.1	3.3	8.7	31.6
HOP-92	2.3	5.8	28.2	1.4	4.0	14.5	1.4	9.5	19.5	2.0	4.3	9.1
NCI-H226	10.0	25.7	64.6	3.2	13.5	47.9	4.9	>100	>100	3.0	12.9	79.4
NCI-H23	2.6	8.5	>100	1.2	5.0	33.9	2.1	12.3	30.9	2.1	5.0	16.2
NCI-H322M	4.4	77.6	>100	2.8	17.0	>100	5.6	>100	>100	3.0	12.0	>100
NCI-H460	3.4	11.5	>100	1.4	4.1	14.5	2.6	>100	63.1	1.7	3.7	8.1
NCI-H522	4.2	16.2	67.6	2.0	5.2	18.6	3.1	43.7	79.4	2.0	4.6	12.0
mean	4.6	23.6	>100	2.1	9.0	>100	3.4	>100	>100	2.3	7.2	>100
***Colon Cancer***												
COLO 205	3.5	10.0	38.0	1.9	3.5	6.5	3.9	56.2	50.1	2.0	3.6	6.5
HCC-2998	3.5	21.9	>100	1.9	7.4	47.9	3.0	>100	>100	2.2	6.9	>100
HCT-116	3.3	11.2	43.7	1.3	3.6	10.2	2.4	>100	33.9	1.7	3.5	7.2
HCT-15	3.4	30.9	>100	2.2	11.5	97.7	3.0	>100	>100	2.1	7.1	>100
HT29	6.0	46.8	>100	2.3	6.0	28.8	3.2	>100	43.7	2.0	4.7	18.2
KM12	3.6	16.2	>100	1.0	4.2	24.0	2.8	>100	43.7	2.1	5.4	36.3
SW-620	4.0	>100	>100	2.6	11.7	>100	3.3	>100	>100	2.5	7.1	83.2
mean	3.9	>100	>100	1.9	6.9	>100	3.1	>100	>100	2.1	5.5	>100
***CNS Cancer***												
SF-268	11.5	47.9	>100	3.2	12.6	47.9	4.8	>100	64.6	3.2	11.0	41.7
SF-295	2.5	6.9	>100	0.7	3.3	19.1	1.8	>100	34.7	1.7	4.4	13.8
SF-539	13.5	32.4	79.4	3.1	9.5	32.4	7.1	>100	>100	2.8	6.6	24.5
SNB-19	4.4	21.9	87.1	2.6	10.5	47.9	3.5	>100	>100	3.1	11.5	57.5
SNB-75	7.6	20.9	49.0	2.3	6.0	21.4	2.8	>100	50.1	2.3	5.9	20.0
U251	3.8	13.5	38.9	1.7	3.7	8.3	2.3	>100	30.9	1.7	3.5	7.2
mean	7.2	23.9	>100	2.3	7.6	29.5	3.7	>100	>100	2.5	7.1	27.5
***Melanoma***												
LOX IMVI	3.8	25.1	>100	1.7	4.3	11.7	2.6	>100	38.9	2.0	4.5	9.8
MALME-3M	6.2	21.9	69.2	2.5	5.6	21.4	2.6	69.2	52.5	2.3	5.0	13.2
M14	4.1	22.9	>100	2.7	7.6	38.0	3.8	>100	>100	2.5	6.8	>100
MDA-MB-435	3.6	89.1	>100	3.0	12.6	>100	4.6	>100	>100	2.8	10.7	>100
SK-MEL-2	3.2	10.2	47.9	2.3	5.5	18.2	4.6	52.5	60.3	2.2	5.5	20.9
SK-MEL-28	6.9	22.9	63.1	3.2	12.0	36.3	4.4	>100	97.7	3.0	9.5	>100
SK-MEL-5	4.9	17.4	43.7	2.1	4.8	12.9	5.2	>100	95.5	3.1	10.7	52.5
UACC-257	3.8	23.4	>100	1.9	5.0	22.4	3.1	55.0	>100	2.3	7.4	>100
UACC-62	2.9	13.8	75.9	1.3	10.7	38.9	1.9	>100	>100	1.5	6.3	36.3
mean	4.4	27.4	>100	2.3	7.6	>100	3.6	>100	>100	2.4	7.4	>100
***Ovarian Cancer***												
IGROV1	6.2	95.5	>100	3.8	33.9	>100	4.7	>100	>100	3.0	7.8	>100
OVCAR-3	3.5	11.0	52.5	1.4	5.5	27.5	2.3	83.2	34.7	1.9	4.0	8.5
OVCAR-4	3.7	18.6	>100	2.2	6.9	38.9	2.2	>100	53.7	1.7	3.9	NT
OVCAR-5	6.6	50.1	>100	2.9	11.0	72.4	5.5	>100	>100	2.8	8.9	>100
OVCAR-8	4.0	14.8	47.9	1.8	4.7	14.8	2.9	>100	72.4	1.8	3.8	7.9
NCI/ADR-RES	3.4	18.2	>100	1.4	5.9	38.0	2.4	>100	52.5	1.9	5.2	25.7
SK-OV-3	11.2	26.9	63.1	2.7	6.6	24.0	9.8	>100	74.1	3.0	7.4	30.9
mean	5.5	33.6	>100	2.3	10.6	>100	4.3	>100	>100	2.3	5.9	>100
***Renal Cancer***												
786-0	5.8	30.2	>100	3.3	14.1	87.1	5.5	>100	>100	2.0	4.8	17.0
A498	2.2	9.5	67.6	1.2	5.4	28.8	1.1	32.4	52.5	1.4	4.2	25.1
ACHN	3.5	10.7	33.9	1.5	5.9	26.3	2.1	>100	55.0	1.9	3.8	7.6
CAKI-1	3.2	14.8	>100	2.1	11.0	72.4	2.3	>100	>100	2.3	7.6	97.7
RXF 393	5.0	18.2	44.7	2.5	7.1	28.2	3.1	>100	58.9	1.9	4.4	9.5
SN12C	4.5	20.9	93.3	2.3	9.5	40.7	3.2	>100	>100	2.3	7.6	49.0
TK-10	5.8	25.1	>100	3.2	15.1	>100	4.6	>100	83.2	2.2	5.8	37.2
UO-31	3.2	14.1	60.3	1.9	8.1	51.3	2.5	91.2	>100	2.0	5.0	26.3
mean	4.1	17.9	>100	2.3	9.5	>100	3.0	>100	>100	2.0	5.4	**33.7**
***Prostate Cancer***												
PC-3	2.5	8.7	>100	0.9	4.3	26.9	1.7	>100	30.2	1.8	5.0	27.5
DU-145	4.7	22.9	>100	3.1	10.7	55.0	3.4	>100	52.5	2.7	6.9	58.9
mean	3.6	15.8	>100	2.0	7.5	40.9	2.5	>100	**41.3**	2.3	6.0	**43.2**
***Breast Cancer***												
MCF7	3.0	22.4	>100	2.5	11.0	50.1	3.4	>100	63.1	2.1	5.9	25.1
MDA-MB-231/ATCC	6.0	28.8	>100	1.9	5.2	25.1	3.0	>100	91.2	1.7	3.7	7.9
HS 578T	4.0	28.8	>100	2.0	8.5	>100	3.2	>100	>100	2.7	11.5	>100
BT-549	12.6	32.4	81.3	3.0	9.8	37.2	5.9	>100	69.2	4.0	15.1	49.0
T-47D	3.9	23.4	>100	2.2	12.6	>100	3.2	>100	>100	2.3	6.9	69.2
MDA-MB-468	2.6	9.3	77.6	1.4	5.5	51.3	2.0	>100	>100	1.8	5.0	33.9
mean	5.3	24.2	>100	2.2	8.8	>100	3.4	>100	>100	2.4	8.0	>100
**Full panel GI_50_ MG-MID**	**4.9**			**2.2**			**3.3**			**2.3**		

**Figure 15:**
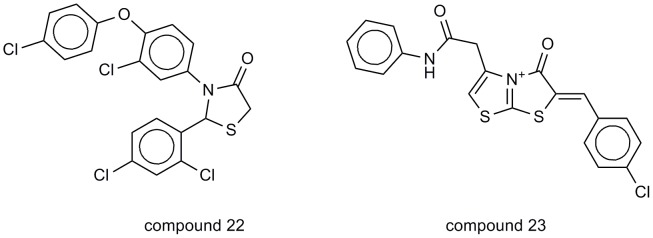
Two compounds showing feature trees similarity of 0.876 to the compound 12b were also retrieved in compare analysis biological pattern similarity with correlation above 0.6.

We decided to inspect the pattern of the 60 cell line dose response produced by the compounds so we used a program for pattern recognition (COMPARE program [29]). This program is provided by NCI and is based on the fact that while the particular inhibitory response of a single cell line might be relatively uninformative, the pattern of response of the cell lines as a group can be used to rank a compound according to the likelihood of sharing common mechanisms. The COMPARE algorithm (a computer program) qualifies this pattern and searches an inventory of screened agents to compile a list of the compounds that have the most similar patterns of cellular sensitivity and resistance. Interestingly, when it was applied using our profiled compounds, two of the compounds retrieved in the pattern similarity searching were those which showed high structural similarity during cancericidal activity verification using Ftrees (see Figure 15). This gave us reliability in usage of Ftrees in cancericidal verification. Moreover, this inspired us with a future work based on the idea of screening ligands using parallel filtration against wide panel of inhibitors by deploying an extremely fast algorithm like Ftrees. This can be a good choice when someone wants to profile database of compounds against a panel of inhibitors belonging to one or more class without having to develop a common-feature pharmacophore or QSAR model.

#### Mechanistic studies of the antiproliferative activity

In this study, we verified the cancericidal activity first and followed it by high-throughput kinase profiling against a panel of 200 kinases [30]. We didn't focus on clinically validated targets [16] and prefer to see the whole picture because we believe that the activity of an anticancer drug emerges from the perturbation of multiple cellular pathways. This was the main reason too that we developed the computational protocol to generate a general kinase inhibitor. This can enable us to highlight affected kinases that don't belong to the landscape of the clinical kinase targets (about 42 kinases as shown in Figure S1 of the supplementary data). Additionally, this can trigger a future work regarding optimization of the inhibitor while aiming to increase its selectivity towards these untargeted kinases to be able to functionally annotate them in the complex cell signaling system.

**Figure 16:**
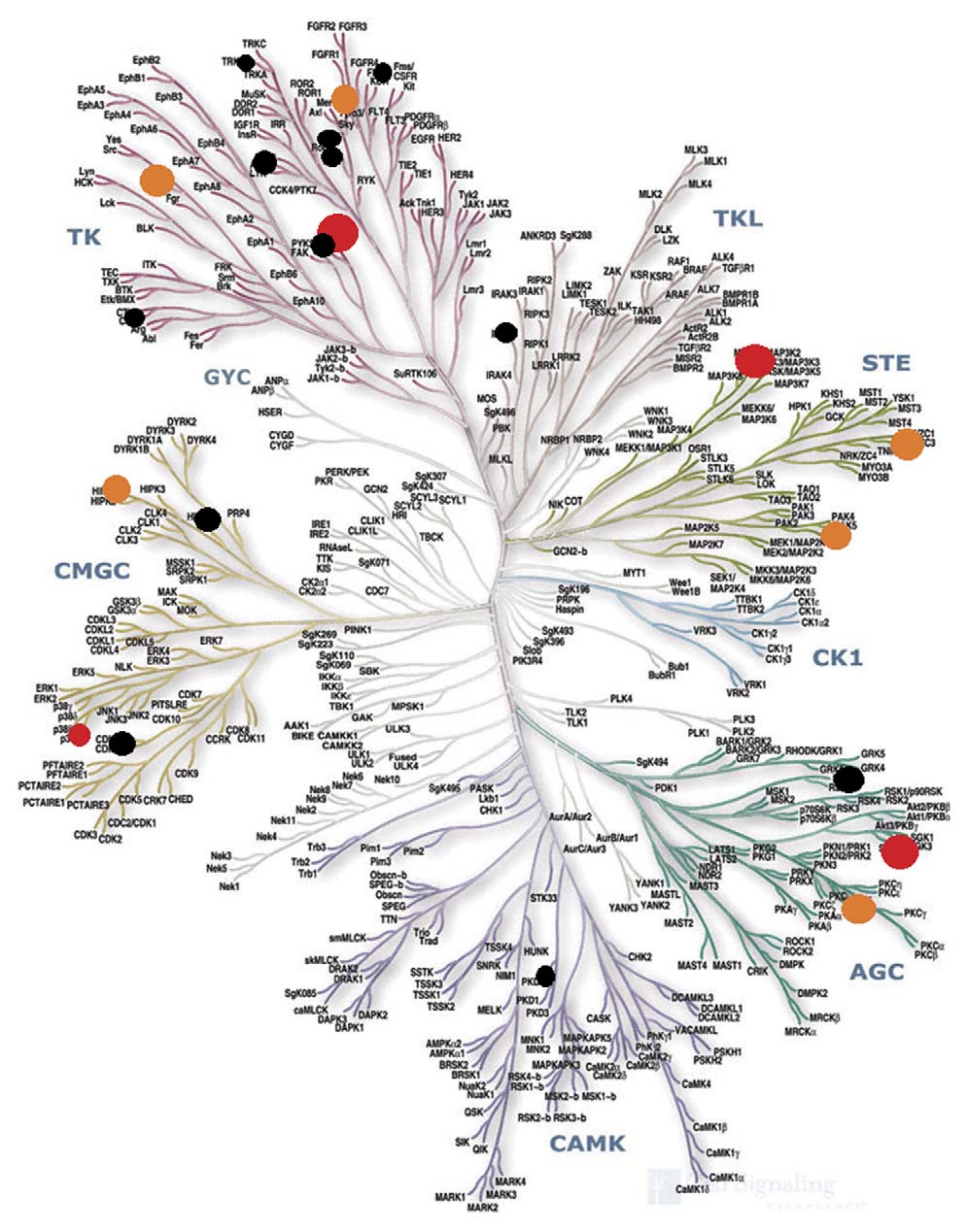
Kinome map of the compound 12a inhibition % is scaled using color coding as follow: 20%–40% black circles, 40%–70% orange circles and >70% in red circles. The radius of the circle corresponds to the inhibition % within this range.

The kinase selectivity pattern was explored. Compound **12a** was assayed using KINEX™ protein kinase microarray-based small molecule inhibitor profiling platform. This microarray comprises 200 protein kinases belonging to various kinase families as AGC, CAMK, CMGC, TK, STE, TKL and other (OPK). Our compound showed significant inhibition at 10 µM concentration against a panel of tyrosine kinases (TKs) as Zap70 (78%), FYNA (67%) and RET kinase (44%), while it showed weak activity against Met, RON, Syk, FLT1 and CSK tyrosine kinases (20 to 40%). The kinome inhibition map is illustrated in Figure 16. The data are also given in Table S1 of the supplementary data.

Compound **12a** also showed significant inhibition against other protein kinases as STE family kinases: MINK1 (58%) and Mekk2 (87%). These enzymes were recently reported to have a relation with the progression of some types of cancer. For instance, Mink-1 kinase or Misshapen-like kinase-1 is a serine/threonine kinase belonging to (GCK) family [31]. The role of Mink1 in cancer has been recently studied [32]. Interestingly, previous reports have shown that oncogenic KRas activity causes increased MINK1 activity and expression [33], and that MAP4K4 expression is heightened in tumor cell lines and tumor tissues compared with their normal counterparts [34], [35]. Those reports suggested the interesting possibility that Msn kinases (which include MINK1) might be involved in inhibiting the tumor-suppressing functions of TGF-β/BMP in various cancers.

On the other hand, Mitogen-activated protein kinase kinase kinase 2 (MEKK2) is a member of the MAPK signaling pathway which is able to activate c-Jun N-terminal kinase (JNK) and ERK5 [36], [37]. When MEKK2 gene was knocked out in lab animals it showed effect on the T-cell receptor, epidermal growth factor (EGF) and fibroblast growth factor 2 (FGF-2) signaling pathways [36], [38], [39]. It has been reported that MEKK2 is able to discriminate tumor from normal cells [40], suggesting that MEKK2 may play important roles in the development of cancer.

Additionally, the microarray results triggered us to further explore accurately one of the interesting targets which is found in hemopoeitic tumors; Syk kinase. Recent reports mentioned Syk kinase as a highly expressed kinase in different B-cell malignancies. Antigen-independent phosphorylation of Syk has been observed in follicular lymphoma, diffuse large B-cell lymphoma, mantle cell lymphoma and B-cell chronic lymphocytic leukemia. Down regulation of Syk in some B-cell malignancies resulted in decreased phosphorylation of downstream signaling molecules and inhibition of proliferation and survival, indicating that constitutively active Syk contributes to the growth of these malignancies [41]–[43].

**Figure 17:**
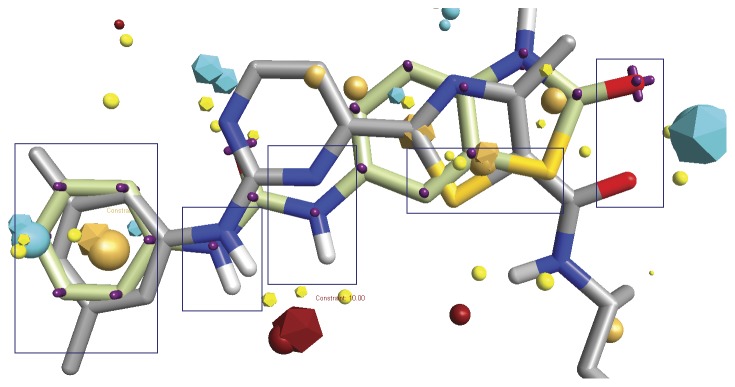
Similarity of urea-based derivative 12a with a Syk kinase inhibitor using field alignment method. The boxes highlight regions of high field similarity. Both of the inhibitors are having thiazole moiety if we considered structural similarity.

Syk inhibition was of primary concern for us because a recent study conducted by Bamborough et al [44] regarding selectivity of kinase inhibitor fragments showed an interesting result regarding the 1,3 diphenylurea fragment. It showed a mean percentage inhibition profile of 87% against a group of compounds conducted in their study although the kinase has not been reported to bind in DFG-out mode before. We tried to understand the observed activity against Syk by finding out how much the urea-based inhibitor is similar to known Syk inhibitors using field similarity. This was carried out by field aligning compound (**12a**) against a set of 141 Syk inhibitors collected from different literature resources (File S2) [45]–[57]. According to the alignment scores, the compound shown in Figure 17 showed highest similarity with our ligand. Focusing on the features similarity, it is clear that our inhibitor shares a lot of common features with the reported inhibitor. This can serve to develop a more selective inhibitor for this specific kinase.

It is obvious that field alignment method can be used to understand an observed activity without the need to develop a common field template or pharmacophore which may ignore some important features that serve to increase selectivity simply because it is not shared among all the members of the training set. Besides, these techniques usually are based on a group of ligands selected on some basis and not all the inhibitors found for a certain class which may cause loss of important information regarding the essential features if the division of the compounds to training and test set was not ideal.

Cell cycle analysis was also performed to examine the influence of these derivatives on the progression of the cell cycle. Compound **12a** was selected for a 24 h treatment of MCF-7 cells at two concentrations; 1, 10 µM. The result showed that compound **12a** induced G_0_/G_1_ arrest in MCF-7 cells, and the effect was observed in a dose-dependent manner. A 24 h treatment with 1, 10 µM concentration of **12a** resulted in a significant accumulation of MCF-7 cells in the G0/G1 phase (60.2% and 68.6%, respectively compared to 58.5% in the control). Slight apoptosis (sub G_0_) was observed at the higher concentration (10 µM) as compared to the control (6.6% against 1.3% in the control) (Table 5).These findings indicated a continuing impairment of cell division and further supports that compound **12a** acts as an antiproliferative agent. The inhibitor seems to have cytostatic activity with mild cytotoxicity at higher doses. The cytotoxicity, however, is exacerbated in more potent derivatives like **12d** and that was obvious from the cytotoxicity assay carried out on the panel of 60 cell lines where growth inhibition exceeded the inhibitory limit (100% inhibition) to the lethal effect in some cell lines of melanoma and colon cancer but this is not the case in all the series.

**Table 5:** Effect of 12a on MCF-7 cell cycle progression.

Concentration (Mm)	Cell cycle distribution (%)			
	Sub G_0_	G_0_/G_1_	S	G_2_/M
Control	1.35	58.50	36.65	4.85
1	1.14	60.27	34.79	4.93
10	6.63	68.63	30.32	1.05

Allover, it is clear here that this series derive their efficacy from simultaneously targeting multiple kinases. In other words, they exhibit their “emergent” properties in their ability to perturb multiple kinase pathways. However, the kinase inhibition may be one of the mechanisms by which these compounds exerts their anticancer effect as we have strong belief that compounds which are urea-based have another more leading mechanism that is responsible for the strong antineoplastic activity like tubulin formation inhibition but this was beyond the scope of the study.

## Conclusions

This study is hoped to serve as a stimulant for new thoughts in the quest for rational design of urea-based antineoplastic kinase inhibitors. The study highlights important facts regarding the different binding modes that urea derivatives can assume in the kinases binding sites besides the different configurations taken by the kinase enzymes themselves. The structural knowledge retrieved from a wide panel of urea-kinases complexes was deployed in creating a screening protocol that filter urea-based ligands through multiple field-based pharmacophores, each representing a kinase complex. Thus, we considered all the possible conformations and probable binding motifs that can render the urea-ligand a kinase inhibitor. Besides, we provided a tool for checking cancericidal activity of the candidate hits by deploying the feature-based similarity searching of the candidate against NCI database using the extremely fast algorithm of Ftrees. The study was verified experimentally through a successful attempt of developing novel urea-based benzothiazolone derivatives with potent antineoplastic activity. Mechanistic studies carried out using kinase microarray technique and cell-flow cytometry casted a shadow on the possible mode of action of this novel series.

## Experimental

### Molecular modeling

#### Urea-derivatives retrieval from commercial and In house libraries

Commercial databases supplied with MOE 2010 together with our in house library were exposed to substructure 2D searching using urea fragment as a query (see supplementary data Text S6 for details regarding these commercial databases). Accelrys Pipeline Pilot 8 (Accelrys Software Inc., San Diego, CA, USA) was used in this process by using SMARTS filter module to carry out substructure search.

#### Simultaneous screening using SVM and Bayesian models

The details of these models construction, descriptors used, test and training set division, internal and external validation are given in the supplementary data (Text S3 and Text S4).

#### Screening using Structure-based pharmacophores

The structure-based pharmacophore protocol implemented in Accelrys Discovery studio 3.0 (Accelrys Software Inc., San Diego, CA, USA) was used. The details are given in the supplementary data (Text S5).

#### Ligands profiling using multiple field templates

PDB of urea-derivatives kinase complexes were retrieved from kinase database supplied with MOE 2010. The pdb files were processed in Accelrys Discovery studio 3 and divided into ligands and their corresponding proteins. They were used as inputs for Field Align 2.1 software (Cresset BioMolecular Discovery, Hertfordshire, UK) to generate field pharmacophores. Urea derivatives retrieved from different databases were aligned using conformation hunting method which applies Monte Carlo approach combined with fast molecular dynamics for ring conformations. XED forcefield was used for minimization of the conformations and charges assignment. The default parameters were used, where maximum number of conformers was set to 200. Number of high T-dynamics for flexible ring was set to 10. Gradient cut-off for conformer minimization = 0.5 Kcal/mol/A.

#### Antineoplastic activity verification using Ftrees

Each ligand retrieved via field template ligand profiling was checked for antineoplastic activity via BioSolveIt Ftrees v2.4. The ligands were searched against NCI database. Minimum similarity was set to 0.8. Search algorithm used was the Split-Search algorithm where a divide and conquer algorithm which recursively splits the Feature Trees into smaller and smaller subtrees. The best matches of the smallest subtrees are calculated first and used to calculate the best matching at the next level of recursion, and so on until the best matching between the complete trees has been found. Gap penalty was set to global.

#### Field alignment usage to understand Syk inhibition

Field alignment was used to retrieve a Syk inhibitor which is most similar to our ligand (12a) in order to understand the reason beyond Syk inhibition. This was carried out by field aligning compound (12a) against a set of 141 Syk inhibitors collected from different literature resources (File S2) [45]–[57].The parameters were used just like that used in ligand profiling.

### Chemistry

All chemicals used were purchased from Aldrich (USA). Melting points are uncorrected and determined in one end open capillary tubes using Stuart Scientific apparatus. Microanalysis was carried out at Department of Chemistry, Humbolt Universität zu Berlin. The NMR spectra were recorded on a BrukerAvance II 500-OC NMR spectrometer. ^1^H spectra were run at 500 MHz and ^13^C spectra were run at 126 MHz in deuterated chloroform (CDCl_3_) or dimethylsulfoxide (DMSO-d_6_). Chemical shifts are quoted in **δ** and were related to that of the solvents. The high resolution ESI-FTICR-MS spectra were recorded using a LTQ FT Ultra mass spectrometer (Thermo Fisher Scientific). TLC were carried out using Art.DC-Plastikfolien, Kieselgel 60 F254 sheets (Merck, Darmstadt, Germany), the developing solvents were DCM/MeOH (9∶1), with visualization under U.V. light (254 nm).

### Synthesis of 6-nitro-2, 3-dihydrobenzo[d]thiazol-2-one (5)

#### Method (A)

A mixture of p-nitroaniline (**1**) (13.8 g, 100 mmol, 1.0 equiv) and ammonium thiocyanate (100 mmol, 1.0 equiv) in glacial acetic acid (100 ml) was cooled and stirred. To this solution bromine (100 mmol, 1.0 equiv) was added dropwise at such a rate to keep the temperature below 10°C throughout the addition. Stirring was continued for an additional 3 h then hot water (200 ml) was added to dissolve the formed precipitate and the solution was neutralized with NaOH solution (25%). The formed precipitate was filtered, washed with water and dried; recrystallization out of ethanol/water gave a yellow solid of 6-nitro-2-amino-2, 3-dihydrobenzo[d]thiazole (**2**), yield (75%).

A solution of (**2**) (10 g, 50 mmol, 1.0 equiv) in concentrated sulfuric acid (200 ml) was cooled to −10°C. To this solution, cold solution of NaNO_2_ (6.9 g, 100 mmol, 2.0 equiv) in water (10 ml) was added dropwise keeping temperature below 10°C. Stirring was continued for 15 min. at 0–10°C, then for 60 min. at room temperature. The solution is then added to a boiling mixture of concentrated sulfuric acid (150 ml) and water (300 ml), and refluxed for 30 minutes. The formed precipitate is filtered, washed with water and dried. Crystallization from ethanol/water gave 6-nitro-2, 3-dihydrobenzo[d]thiazol-2-one (**5**) as a light brown solid, with an overall yield (26%).

#### Method (B)

A solution of 2-mercapto-2, 3-dihydrobenzo[d]thiazole (**3**) (11.37 g, 68 mmol, 1.0 equiv) in concentrated sulfuric acid (120 ml) was cooled to −10°C. To this solution, a cold solution of NaNO_3_ (5.78 g, 68 mmol, 1.0 equiv) in concentrated sulfuric acid (100 ml) was added dropwise over 60 min. keeping temperature at 0°C. The resulting solution is poured on ice/water mixture (1 litre), filtered and dried. Recrystallization from glacial acetic acid gave 6-nitro-2-mercapto-2, 3-dihydrobenzo[d]thiazole (**4**) as pale yellow solid, yield (80%).

To a stirred solution of (**4**) (8.35 g, 50 mmol) in 25% aqueous NaOH (25 ml) at room temperature was added slowly a solution of 10% KMnO_4_ over 30 min. The reaction mixture is heated to 80–90°C for 30 min and the MnO_2_ sludge is filtered off and washed with hot water. The filtrate was acidified to pH = 2 with concentrated HCl and refluxed until the evolving of SO_2_ is finished. The obtained precipitate was collected by filtration, washed with water and dried. Recrystallization from ethanol/water gave 6-nitro-2, 3-dihydrobenzo[d]thiazol -2-one (**5**) as a light brown solid, with an overall yield (56%).

#### General procedure for the synthesis of 3-substitutedbenzyl-6-nitro-2, 3-dihydrobenzo[d]thiazol-2-one (6a-b)

To a stirred solution of (**5**) (2.943 g, 15 mmol, 1.0 equiv) in acetone (22.5 ml), water (0.75 ml) and 85% KOH (0.99 g, 30 mmol, 2.0 equiv) was added the appropriate benzyl chloride (15 mmol, 1.0 equiv) in one portion. The reaction mixture was refluxed for 24 hours, cooled to 5°C, 60 gm ice-water was added and the mixture was stirred at 0–10°C for 1 hour. The obtained solid was filtered, washed with old water then diethyl ether, dried at room temperature and Crystallized from acetone/water to give pure compounds (**6a–c**).

#### 3-(3, 4-dichlorobenzyl)-6-nitro-2, 3-dihydrobenzo[d]thiazol-2-one (6a)

Yield: 92%; ^1^H NMR (500 MHz, DMSO-d_6_) δ 8.77 (d, J = 2.5 Hz, 1H), 8.23 (dd, J = 8.9, 2.4 Hz, 1H), 7.67 (d, J = 2.2 Hz, 1H), 7.61 (d, J = 8.3 Hz, 1H), 7.55 (d, J = 9.0 Hz, 1H), 7.26 (dd, J = 8.4, 2.1 Hz, 1H), 5.29 (s, 2H).; ^13^C NMR (126 MHz, DMSO-d_6_) δ 169.95, 143.07, 141.63, 136.27, 131.36, 131.01, 130.57, 129.43, 127.34, 122.79, 119.44, 111.57, 109.48, 44.60; HRMS (ESI), m/z (M)^+^: calcd 353.9632, obsd 353.9641.

#### 3-(2, 4-dichlorobenzyl)-6-nitro-2, 3-dihydrobenzo[d]thiazol-2-one (6b)

Yield: 90%; ^1^H NMR (500 MHz, DMSO-d_6_) δ 8.82 (d, J = 2.4 Hz, 1H), 8.22 (dd, J = 9.0, 2.5 Hz, 1H), 7.73 (d, J = 2.2 Hz, 1H), 7.41 (d, J = 9.0 Hz, 1H), 7.37 (dd, J = 8.4, 2.2 Hz, 1H), 6.99 (d, J = 8.4 Hz, 1H), 5.30 (s, 2H).; ^13^C NMR (126 MHz, DMSO-d_6_) δ 169.74, 143.10, 141.76, 133.15, 132.86, 131.28, 129.25, 128.86, 127.82, 122.86, 122.76, 119.47, 111.65, 43.70; HRMS (ESI), m/z (M)^+^: calcd 353.9632, obsd 353.9641.

#### General procedure for the synthesis of 3-substitutedbenzyl-6-amino-2, 3-dihydrobenzo[d]thiazol-2-one (7a–b)

To a solution of the appropriate 3-substitutedbenzyl-6-amino-2, 3-dihydrobenzo[d]thiazol -2-one (**6a–b**) (5 mmol) in ethanol/THF mixture (3∶1) (100 ml) was added 10% pd/C (200 mg) and the mixture was hydrogenated in a bar-shaker hydrogenator at 35 psi at room temperature for 6 hr. The mixture was filtered on cellite; the filtrate was concentrated to give the crude amines (**7a–b**). Recrystallization from diethyl ether gave pure compounds (**7a–b**).

#### 3-(3, 4-dichlorobenzyl)-6-amino-2, 3-dihydrobenzo[d]thiazol-2-one (7a)

Yield: 95%; ^1^H NMR (500 MHz, Chloroform-d) δ 7.31 (d, J = 2.2 Hz, 1H), 7.19 (dd, J = 8.2, 2.3 Hz, 1H), 7.03 (d, J = 8.3 Hz, 1H), 6.72 (d, J = 2.4 Hz, 1H), 6.60 (dd, J = 8.5, 2.3 Hz, 1H), 6.49 (d, J = 8.5 Hz, 1H), 4.95 (s, 1H), 3.62 (s, 2H); ^13^C NMR (126 MHz, Chloroform-d) δ 169.77, 143.04, 135.68, 133.04,132.05, 130.87, 129.07, 128.83, 126.47, 123.82, 113.80, 111.59, 109.11, 45.06; HRMS (ESI), m/z (M+H)^+^: calcd 324.9969, obsd 324.9963.

#### 3-(2, 4-dichlorobenzyl)-6-amino-2, 3-dihydrobenzo[d]thiazol-2-one (7b)

Yield: 99%; ^1^H NMR (500 MHz, Chloroform-d) δ 7.35 (d, J = 2.2 Hz, 1H), 7.05 (dd, J = 8.3, 2.3 Hz, 1H), 6.82 (d, J = 8.4 Hz, 1H), 6.72 (d, J = 2.5 Hz, 1H), 6.54 (d, J = 8.6 Hz, 1H), 6.47 (dd, J = 8.6, 2.4 Hz, 1H), 5.06 (s, 2H), 3.44 (s, 2H); ^13^C NMR (126 MHz, Chloroform-d) δ 169.79, 143.15, 134.10, 133.27, 131.30, 129.50, 128.75, 128.49, 127.65, 123.69, 113.91, 111.72, 109.01, 43.12; HRMS (ESI), m/z (M+H)^+^: calcd 324.9969, obsd 324.9963.

#### General procedure for the synthesis of substituted Phenyl isocyanates (11a–h)

A mixture of the appropriate acid (**8a–h**) (20 mmol) and thionyl chloride (5 ml) were stirred under reflux for 5 h. The mixture was evaporated under reduced pressure to give the crude acid chloride (**9a–h**) which was used as such in the next step.

To a well stirred suspension of sodium azide (1.95 g, 15 mmol, 1.5 equiv) in acetone (20 ml) at 0°C was added the appropriate acid chloride (**9a–h**) (10 mmol, 1.0 equiv). The reaction mixture was stirred vigorously for 30 min at 0°C. The reaction mixture was thereafter filtered through cotton wool and the solvent was evaporated in vacuo to give the corresponding azide (**10a–h**) which was used as such in the next step.

A solution of the appropriate azide (**10a–h**) (3 mmol) in dry benzene (5 ml) was heated at 70°C for 3 h. The solvent was then evaporated under reduced pressure to give the corresponding isocyanate (**11a–h**) which was used as such in the next step.

#### General procedure for the synthesis of N-(3-substitutedbenzyl-2-oxo-2, 3-dihydrobenzo[d]thiazol -6-yl)-3-substitutedphenylurea or thiourea (12a-l)

To a solution of 3-substitutedbenzyl-6-amino-2, 3-dihydrobenzo[d]thiazol-2-one (**7a–b**) (0.3 mmol, 1.0 equiv) in methylene chloride (10 ml) was added the appropriate phenyl isocyanates or isothiocyanate (**11a–k**) (1.0 mmol, 3.0 equiv) and the mixture was stirred at room temperature for 24 h. After the completion of the reaction as monitored by TLC, the formed precipitate was filtered, washed with methylene chloride, dried and recrystallized from methanol to give the target products (**12a–l**).

In case of the isothiocyanate derivative, after 24 h, the reaction mixture was evaporated and the residue was triturated with diethyl ether and the resulting solid was filtered and washed with diethyl ether, dried and recrystallized from DCM/Hexane to give the target product (**12c**).

#### 1-(3-(2, 4-dichlorobenzyl)-2-oxo-2, 3-dihydrobenzo[d]thiazol-6-yl)-3-phenylurea (12a)

Yield: 97%; ^1^H NMR (500 MHz, DMSO-d_6_) δ 8.76 (s, 1H), 8.71 (s, 1H), 7.92 (d, J = 2.2 Hz, 1H), 7.72 (d, J = 2.1 Hz, 1H), 7.48 – 7.43 (m, 2H), 7.39 (dd, J = 8.5, 2.2 Hz, 1H), 7.33 – 7.24 (m, 3H), 7.06 (d, J = 8.7 Hz, 1H), 7.01 – 6.91 (m, 2H), 5.19 (s, 2H); ^13^C NMR (126 MHz, DMSO-d_6_) δ 168.81, 152.54, 139.59,137.01, 135.82, 132.92, 132.80, 131.94, 131.16, 129.16, 128.74, 128.72, 127.82, 121.82, 118.14, 117.56, 113.02, 111.61, 42.98; HRMS (ESI), m/z (M+H)^+^: calcd 444.0340, obsd 444.0337.

#### 1-(3-(3, 4-dichlorobenzyl)-2-oxo-2, 3-dihydrobenzo[d]thiazol-6-yl)-3-phenylurea (12b)

Yield: 90%; ^1^H NMR (500 MHz, DMSO-d_6_) δ 8.75 (s, 1H), 8.71 (s, 1H), 7.88 (d, J = 2.2 Hz, 1H), 7.70 – 7.55 (m, 2H), 7.46 (d, J = 7.9 Hz, 2H), 7.35 – 7.18 (m, 5H), 6.97 (t, J = 7.3 Hz, 1H), 5.18 (s, 2H); ^13^C NMR (126 MHz, DMSO-d_6_) δ 168.94, 152.55, 139.58, 137.05, 135.78, 131.27, 131.10, 130.98, 130.35, 129.33, 128.74, 127.34, 121.85, 121.81, 118.15, 117.49, 113.00, 111.70, 44.08; HRMS (ESI), m/z (M+H)^+^: calcd 444.0340, obsd 444.0337.

#### 1-(3-(2, 4-dichlorobenzyl)-2-oxo-2, 3-dihydrobenzo[d]thiazol-6-yl)-3-phenylthiourea (12c)

Yield: 95%; ^1^H NMR (500 MHz, DMSO-d_6_) δ 9.89 (s, 1H), 9.75 (s, 1H), 7.87 (d, J = 2.1 Hz, 1H), 7.73 (d, J = 2.2 Hz, 1H), 7.51 – 7.46 (m, 2H), 7.40 (dd, J = 8.4, 2.2 Hz, 1H), 7.37 – 7.29 (m, 3H), 7.18 – 7.08 (m, 2H), 6.97 (d, J = 8.4 Hz, 1H), 5.23 (s, 2H); ^13^C NMR (126 MHz, DMSO-d_6_) δ 179.92, 169.07, 139.34, 135.25, 133.44, 133.00, 132.86, 131.87, 129.21, 128.80, 128.40, 127.83, 124.48, 123.76, 123.66, 121.16, 119.48, 111.21, 43.09; HRMS (ESI), m/z (M+H)^+^: calcd 460.0111, obsd 460.0106.

#### 1-(4-chlorophenyl)-3-(3-(3, 4-dichlorobenzyl)-2-oxo-2, 3-dihydrobenzo[d]thiazol-6-yl)urea (12d)

Yield: 80%; ^1^H NMR (500 MHz, DMSO-d_6_) δ 8.85 (s, 1H), 8.78 (s, 1H), 7.86 (d, J = 2.3 Hz, 1H), 7.68 – 7.57 (m, 2H), 7.53 – 7.43 (m, 2H), 7.35-7.31 (m, 3H), 7.29 – 7.17 (m, 2H), 5.18 (s, 2H); ^13^C NMR (126 MHz, DMSO-d_6_) δ 168.94, 152.46, 138.61, 137.04, 135.57, 131.27, 131.25, 130.99, 130.35, 129.33, 128.57, 127.34, 125.33, 121.85, 119.68, 117.66, 113.18, 111.70, 44.09; HRMS (ESI), m/z (M+H)^+^: calcd 475.9794, obsd 475.9785.

#### 1-(4-bromophenyl)-3-(3-(2, 4-dichlorobenzyl)-2-oxo-2, 3-dihydrobenzo[d]thiazol-6-yl)urea (12e)

Yield: 95%; ^1^H NMR (500 MHz, DMSO-d_6_) δ 9.86 (s, 1H), 9.80 (s, 1H), 7.92 (d, J = 2.3 Hz, 1H), 7.72 (d, J = 2.3 Hz, 1H), 7.47 – 7.43 (m, 4H), 7.38 (dd, J = 8.3, 2.1 Hz, 1H), 7.30 (dd, J = 8.8, 2.3 Hz, 1H), 7.09 (d, J = 8.7 Hz, 1H), 6.93 (d, J = 8.4 Hz, 1H), 5.19 (s, 2H); ^13^C NMR (126 MHz, DMSO-d_6_) δ 168.77, 152.63, 139.32, 135.99, 132.84, 131.97, 131.44, 131.07, 129.14, 128.70, 127.81, 121.80, 119.70, 117.22, 112.94, 112.83, 112.61, 111.66, 43.01; HRMS (ESI), m/z (M+H)^+^: calcd 521.9445 , obsd 521.9440.

#### 1-(3-chlorophenyl)-3-(3-(3, 4-dichlorobenzyl)-2-oxo-2, 3-dihydrobenzo[d]thiazol-6-yl)urea (12f)

Yield: 75%; ^1^H NMR (500 MHz, DMSO-d_6_) δ 9.16 (s, 1H), 9.05 (s, 1H), 7.88 (s, 1H), 7.71 (d, J = 2.3, 1H), 7.66 – 7.59 (m, 2H), 7.37 – 7.25 (m, 5H), 7.01 (d, J = 7.1 Hz, 1H), 5.18 (s, 2H); ^13^C NMR (126 MHz, DMSO-d_6_) δ 168.95, 152.46, 141.24, 137.04, 135.52, 133.14, 131.27, 130.98, 130.35, 130.33, 129.34, 128.27, 127.35, 121.85, 121.35, 117.61, 117.42, 116.49, 113.13, 111.71, 44.09; HRMS (ESI), m/z (M+H)^+^: calcd 477.9950, obsd 477.9941.

#### 1-(2-chlorophenyl)-3-(3-(3, 4-dichlorobenzyl)-2-oxo-2, 3-dihydrobenzo[d]thiazol-6-yl)urea (12g)

Yield: 75%; ^1^H NMR (500 MHz, DMSO-d_6_) δ 8.89 (s, 1H), 8.82 (s, 1H), 7.87 (d, J = 2.2, 1H), 7.66 – 7.58 (m, 2H), 7.49 (d, J = 8.4 Hz, 2H), 7.41 – 7.19 (m, 5H), 5.17 (s, 2H); ^13^C NMR (126 MHz, DMSO-d_6_) δ 168.94, 152.47, 138.62, 137.03, 135.59, 131.28, 131.23, 130.97, 130.35, 129.33, 128.57, 128.56, 127.33, 125.33, 121.85, 119.66, 119.65, 117.63, 113.14, 111.70, 44.09; HRMS (ESI), m/z (M+H)^+^: calcd 477.9950, obsd 477.9943.

#### 1-(3-(3, 4-dichlorobenzyl)-2-oxo-2, 3-dihydrobenzo[d]thiazol-6-yl)-3-(4-nitrophenyl)urea (12h)

Yield: 90%; ^1^H NMR (500 MHz, DMSO-d_6_) δ 9.49 (s, 1H), 9.01 (s, 1H), 8.20 (d, J = 8.5 Hz, 2H), 7.89 (s, 1H), 7.76 – 7.60 (m, 4H), 7.37 (d, J = 8.5 Hz, 1H), 7.30 – 7.22 (m, 2H), 5.19 (s, 2H); ^13^C NMR (126 MHz, DMSO-d_6_) δ 168.98, 152.01, 146.28, 140.97, 137.01, 135.00, 131.67, 131.27, 131.00, 130.35, 129.34, 127.35, 125.11, 121.92, 117.96, 117.43, 113.58, 111.74, 44.11; HRMS (ESI), m/z (M+H)^+^: calcd 489.0191, obsd 489.0187.

#### 1-(3-(2, 4-dichlorobenzyl)-2-oxo-2, 3-dihydrobenzo[d]thiazol-6-yl)-3-(4-methoxyphenyl)urea (12i)

Yield: 70%; ^1^H NMR (500 MHz, DMSO-d_6_) δ 8.82 (s, 1H), 8.64 (s, 1H), 7.91 (d, J = 2.1 Hz, 1H), 7.72 (d, J = 2.2 Hz, 1H), 7.42 – 7.31 (m, 3H), 7.28 (dd, J = 8.7, 2.2 Hz, 1H), 7.05 (d, J = 8.7 Hz, 1H), 6.94 (d, J = 8.4 Hz, 1H), 6.87 (d, J = 2.3, 2H), 5.19 (s, 2H), 2.60 (s, 3H); ^13^C NMR (126 MHz, DMSO-d_6_) δ 168.79, 154.40, 152.76, 136.10, 132.92, 132.80, 132.64, 131.96, 130.96, 129.15, 128.71, 127.81, 121.77, 119.89, 117.37, 113.94, 112.80, 111.59, 55.12, 42.97; HRMS (ESI), m/z (M+H)^+^: calcd 474.0445, obsd 474.0438.

#### 1-(3-(3, 4-dichlorobenzyl)-2-oxo-2, 3-dihydrobenzo[d]thiazol-6-yl)-3-(4-fluorophenyl)urea (12j)

Yield: 85%; ^1^H NMR (500 MHz, DMSO-d_6_) δ 9.18 (s, 1H), 8.60 (s, 1H), 8.15 (d, J = 8.4 Hz, 1H), 7.90 (d, J = 8.5 Hz, 1H), 7.63 (d, J = 8.4, 2H), 7.33 (d, J = 8.7 Hz, 1H), 7.34 – 7.21 (m, 3H), 7.14 (d, J = 8.0 Hz, 1H), 7.01 (dd, J = 7.5 Hz, 1H), 5.18 (s, 2H); ^13^C NMR (126 MHz, DMSO-d_6_) δ 168.96, 152.20, 137.03, 135.49, 131.28, 130.99, 130.35, 129.32, 127.33, 124.47, 122.44, 121.96, 120.48, 117.36, 115.00, 114.85, 112.88, 111.77, 44.09; HRMS (ESI), m/z (M+H)^+^: calcd 462.0246, obsd 462.0241.

#### 1-(3-(3, 4-dichlorobenzyl)-2-oxo-2, 3-dihydrobenzo[d]thiazol-6-yl)-3-(2, 4-dichlorophenyl)urea (12k)

Yield: 90%; ^1^H NMR (500 MHz, DMSO-d_6_) δ 9.53 (s, 1H), 9.16 (s, 1H), 8.42 (s, 1H), 8.20 (d, J = 9.0 Hz, 1H), 8.11 (d, J = 9.0 Hz, 1H), 7.89 (s, 1H), 7.65 – 7.55 (m, 2H), 7.41 – 7.30 (m, 2H), 7.25 (d, J = 8.3 Hz, 1H), 5.18 (s, 2H); ^13^C NMR (126 MHz, DMSO-d_6_) δ 168.97, 151.99, 137.00, 135.20, 134.82, 131.36, 130.36, 129.31, 128.55, 127.54, 127.31, 126.74, 126.04, 123.40, 123.16, 122.52, 121.97, 117.48, 113.03, 111.77, 44.10; HRMS (ESI), m/z (M+H)^+^: calcd 511.9560, obsd 511.9554.

#### 1-(3-(3, 4-dichlorobenzyl)-2-oxo-2, 3-dihydrobenzo[d]thiazol-6-yl)-3-o-tolylurea (12l)

Yield: 92%; ^1^H NMR (500 MHz, DMSO-d_6_) δ 9.42 (s, 1H), 8.22 (s, 1H), 7.92 (s, 1H), 7.82 (d, J = 8.2 Hz, 1H), 7.66 – 7.59 (m, 2H), 7.35 (d, J = 8.9 Hz, 1H), 7.32 – 7.22 (m, 2H), 7.20 – 7.10 (m, 2H), 6.94 (t, J = 7.6 Hz, 1H), 5.18 (s, 2H), 2.25 (s, 3H); ^13^C NMR (126 MHz, DMSO-d_6_) δ 168.93, 152.81, 137.39, 137.07, 136.19, 131.27, 130.99, 130.34, 130.13, 129.32, 127.68, 127.34, 126.05, 122.63, 121.85, 121.12, 117.20, 112.62, 111.73, 109.48, 44.08, 17.92; HRMS (ESI), m/z (M+H)^+^: calcd 458.0496, obsd 458.0492.

#### N-(3-(3, 4-dichlorobenzyl)-2-oxo-2, 3-dihydrobenzo[d]thiazol-6-ylcarbamothioyl)benzamide (13)

To a solution of 6-amino-3-(3,4-dichlorobenzyl)benzo[d]thiazol-2(3H)-one (**7a**) (0.3 mmol, 1.0 equiv) in methylene chloride (10 ml) was added benzoyl isothiocyanate (1.0 mmol, 3.0 equiv) and the mixture was stirred at room temperature for 24 h. After the completion of the reaction as monitored by TLC the reaction mixture was evaporated and the residue was triturated with hexane and the resulting solid was filtered and washed with hexane, dried and recrystallized from DCM/Hexane to give the target product (**13**).

Yield: 95%; ^1^H NMR (500 MHz, DMSO-d_6_) **δ** 12.53 (s, 1H), 11.64 (s, 1H), 8.05 (d, J = 2.2 Hz, 1H), 8.01 – 7.95 (m, 2H), 7.71 – 7.60 (m, 3H), 7.58 – 7.51 (m, 3H), 7.38 (d, J = 8.6 Hz, 1H), 7.29 (dd, J = 8.3, 2.0 Hz, 1H), 5.23 (s, 2H).; ^13^C NMR (126 MHz, DMSO-d_6_) **δ** 179.75, 169.28, 168.21, 136.86, 134.58, 133.80, 133.12, 132.06, 131.31, 131.04, 130.46 , 129.44, 128.66, 128.41, 127.40, 124.05, 121.38, 119.93, 111.42, 44.25; HRMS (ESI), m/z (M+H)^+^: calcd 488.0061, obsd 488.0055.

### Biological assays

#### In vitro antiproliferative activity

Huh-7 cytotoxicity. The preliminary cytotoxicity of the synthesized compounds were tested against Huh-7 cells by SRB assay as described by Skehan et al [58]. Exponentially growing cells were collected using 0.25% Trypsin-EDTA and plated in 96-well plates at 1000–2000 cells/well. Cells were exposed to the desired concentration of the compounds in DMSO for 72 h and subsequently fixed with TCA (10%) for 1 h at 4oC. After several washings, cells were exposed to 0.4% SRB solution for 10 min in dark place and subsequently washed with 1% glacial acetic acid. After drying overnight, Tris-HCl was used to dissolve the SRB-stained cells and color intensity was measured at 540 nm.

NCI anticancer screening. The human tumor cell lines of the cancer-screening panel are grown in RPMI-1640 medium containing 5% fetal bovine serum and 2 uM L-glutamine. For a typical screening experiment, cells are inoculated into 96 well microtiter plates in 100 pL at plating densities ranging from 5000 to 40,000 cells/well depending on the doubling time of individual cell lines. After cell inoculation, the microtiter plates are incubated at 37°C, 5% CO_2_, 95% air and 100% relative humidity for 24 h prior to addition of experimental drugs.

After 24 h, two plates of each cell line are fixed in situ with TCA, to represent a measurement of the cell population for each cell line at the time of drug addition (Tz). Experimental drugs are solubilized in dimethylsulfoxide at 400-fold the desired final maximum test concentration and stored frozen prior to use. At the time of drug addition, an aliquot of frozen concentrate is thawed and diluted to twice the desired final maximum test concentration with complete medium containing 50 mg/ml gentamicin.

Additional four, 10-fold or log serial dilutions are made to provide a total of five drug concentrations plus control. Aliquots of 100 ml of these different drug dilutions are added to the appropriate microtiter wells already containing 100 ml of medium, resulting in the required final drug concentrations. Following drug addition, the plates are incubated for an additional 48 h at 37°C, 5% CO_2_, 95% air, and 100% relative humidity. For adherent cells, the assay is terminated by the addition of cold TCA. Cells are fixed in situ by the gentle addition of 50 ml of cold 50% (w/v) TCA (final concentration, 10% TCA) and incubated for 60 min at 4°C. The supernatant is discarded, and the plates are washed five times with tap water and air dried. Sulforhodamine B (SRB) solution (100 ml) at 0.4% (w/v) in 1% acetic acid is added to each well, and plates are incubated for 10 min at room temperature.

After staining, unbound dye is removed by washing five times with 1% acetic acid and the plates are air dried. Bound stain is subsequently solubilized with 10 mMtrizma base, and the absorbance is read on an automated plate reader at a wavelength of 515 nm. For suspension cells, the methodology is the same except that the assay is terminated by fixing settled cells at the bottom of the wells by gently adding 50 ml of 80% TCA (final concentration, 16% TCA). Using the seven absorbance measurements [time zero, (Tz), control growth, (C), and test growth in the presence of drug at the five concentration levels (Ti)], the percentage growth is calculated at each of the drug concentrations levels. Three dose response parameters are calculated for each experimental agent. Growth inhibition of 50% (GI_50_), which is the drug concentration resulting in a 50% reduction in the net cell growth. The drug concentration resulting in total growth inhibition (TGI). Lethal concentration 50 ( the drug concentration results in 50% reduction in the initial cell count. Values are calculated for each of these three parameters if the level of activity is reached; however, if the effect is not reached or is exceeded, the value for that parameter is expressed as greater or less than the maximum or minimum concentration tested [59].

### Cell cycle analysis

Effects of **12a** on the stages of the cell cycle were determined using the PI staining technique. Briefly, MCF-7 cells were grown in 25 cm2 flasks at a density of 4×105 cells in 5 ml per flask. After allowing for overnight attachment the cells were treated with the tested drug 10 µM. Cells were incubated for 24 hr then collected by trypsinization, making sure to include the floating cells. After washing in PBS the cells were fixed in ice cold absolute alcohol. Cells were then stained using The CycleTEST™ PLUS DNA Reagent Kit (BD Biosciences, San Jose, CA) according to the manufacturer's instructions. The cell cycle distribution was determined using a FACS Callibur instrument (BD Biosciences, San Jose, CA) [60].

### In vitro profiling of protein kinase inhibitors

The percent inhibition of 200 different kinases by compound **12a** at 10 µM concentration was determined using KINEX™ protein kinase microarray-based small molecule inhibitor profiling platform from Kinexus bioinformatics corporation, Vancouver, Canada. The assay technique depends on Co-incubating a test compound with the biotinylated ATP probe on the protein kinase microarray which allows simultaneous determination of the affinity of the compound against hundreds of protein kinases on the array on a competition binding basis. The kinase to which the compound exhibits binding will experience a reduction of the binding of the ATP probe, and the remaining ATP probe covalently bound to the kinases on the array can be detected with the fluorescently-labeled streptavidin conjugate (see Figure S2 of the supplementary data).

## Supporting Information

Text S1 **Urea-derivatives kinases complexes used to generate field templates.**
(DOCX)

Text S2 **Colour codes used to designate field templates.**
(DOCX)

Text S3 **SVM (Support vector machine) model.**
(DOCX)

Text S4 **Bayesian model.**
(DOCX)

Text S5 **Structure-based pharmacophores.**
(DOCX)

Text S6 **Molecular modeling implementation results.**
(DOCX)

Text S7 **Ftrees results of feature trees similarity against NCI database.**
(DOCX)

Text S8 **Profile of compounds (12b, 12d, 12e, 12k) on the 60 tumor cell line panel at the test dose of 10 uM.**
(DOCX)

Table S1 **Kinase profiling data.**
(DOCX)

Figure S1 **Clinically validated cancer kinome.**
(TIF)

Figure S2 **Schematic depiction of Protein Kinase Microarray-based small molecule inhibitor profiling platform.**
(TIF)

File S1 **Pymol session files of the retrieved urea-based kinase inhibitors complexes.**
(ZIP)

File S2 **Dataset of 141 Syk kinase inhibitors.**
(ZIP)
