# Supplementary material for: Applying Ligands Profiling Using Multiple Extended Electron Distribution Based Field Templates and Feature Trees Similarity Searching in the Discovery of New Generation of Urea-Based Antineoplastic Kinase Inhibitors
Source: PLoS One. 2012 Nov 20;7(11):e49284. doi: 10.1371/journal.pone.0049284 (PMC3502486; doi:10.1371/journal.pone.0049284)

**Urea-derivatives kinases complexes used to generate field templates**


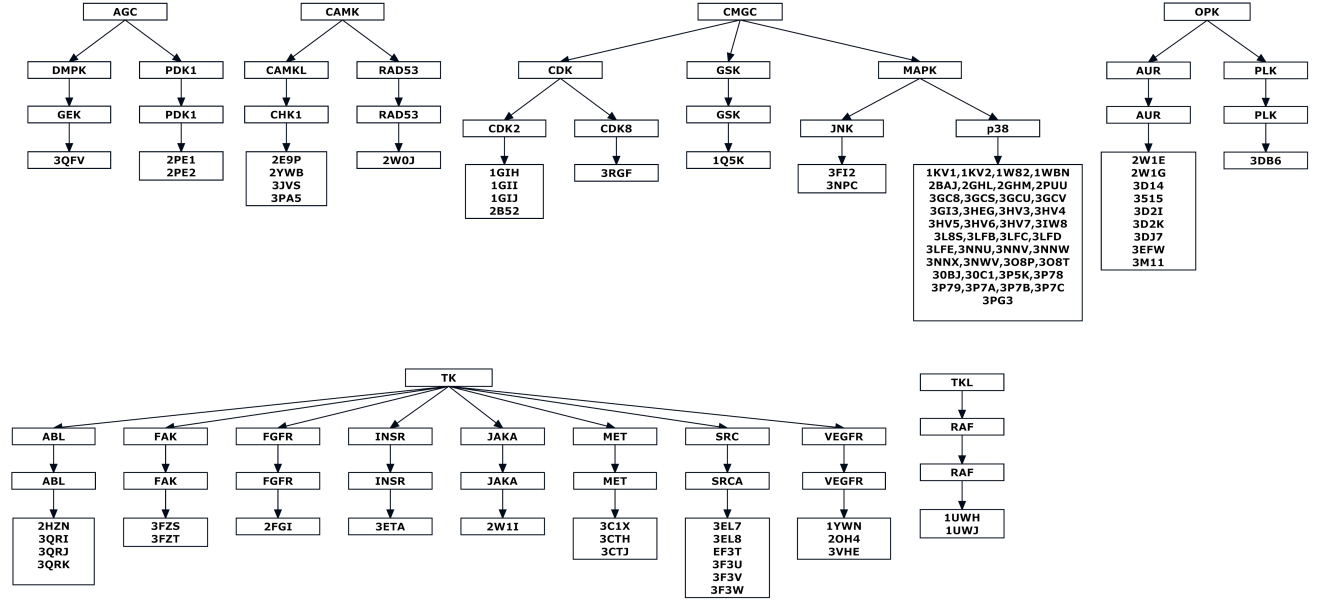


|  | **PDB code** | **Family** | **Subfam** | **DFG** | **aC** | **Binding** | **Urea binding mode** |
| --- | --- | --- | --- | --- | --- | --- | --- |
| **AGC** | **3QFV** | DMPK | GEK | In | In | GY-HRD | Hinge |
|  | **2PE1** | PDK1 | PDK1 | In | In | GY-HRD | DFG and alphaC |
|  | **2PE2** | PDK1 | PDK1 | In | In | GY-HRD | DFG and alphaC |
| **CAMK** | **2E9P** | CAMKL | CHK1 | In | In | GYAHRD | HINGE |
|  | **2YWP** | CAMKL | CHK1 | - | In | GY-HRD | HINGE |
|  | **3JVS** | CAMKL | CHK1 | - | In | ---------- | Allosteric |
|  | **3PA5** | CAMKL | CHK1 | In | In | GYAHRD | HINGE |
|  | **2W0J** | RAD53 | RAD53 | In | - | GYAHRD | HINGE |
| **CMGC** | **1GIH** | CDK | CDK2 | In | - | G--HRD | HINGE |
|  | **1GII** | CDK | CDK2 | In | - | G--HRD | HINGE |
|  | **1GIJ** | CDK | CDK2 | In | - | GY-HRD | HINGE |
|  | **2B52** | CDK | CDK2 | In | - | GY-HRD | Hyd1 |
|  | **3RGF** | CDK | CDK8 | - | - | GYAHRD | DFG and alphaC |
|  | **1Q5K** | GSK | GSK | In | In | GY-HRD | HINGE |
|  | **1KV1** | MAPK | P38 | - | In | GYAHRD | DFG and alphaC |
|  | **1KV2** | MAPK | P38 | - | In | GYAHRD | DFG and alphaC |
|  | **1W82** | MAPK | P38 | - | In | -YAHRD | DFG and alphaC |
|  | **1WBN** | MAPK | P38 | - | In | GYAHRD | DFG and alphaC |
|  | **2BAJ** | MAPK | P38 | - | In | GYAHRD | DFG and alphaC |
|  | **2GHL** | MAPK | P38 | In | In | GYAHRD | DFG and alphaC |
|  | **2GHM** | MAPK | P38 | - | In | GYAHRD | DFG |
|  | **2PUU** | MAPK | P38 | - | In | GYAHRD | alphaC |
|  | **3FI2** | MAPK | JNK | In | - | GYAHRD | Hyd1 |
|  | **3GCQ** | MAPK | P38 | - | - | GYAHRD | DFG and alphaC |
|  | **3GCS** | MAPK | P38 | - | - | GYAHRD | DFG and alphaC |
|  | **3GCU** | MAPK | P38 | Out | In | GYAHRD | DFG and alphaC |
|  | **3GCV** | MAPK | P38 | Out | In | GYAHRD | DFG and alphaC |
|  | **3GI3** | MAPK | P38 | - | In | GYAHRD | DFG and alphaC |
|  | **3HEG** | MAPK | P38 | - | - | GYAHRD | DFG and alphaC |
|  | **3HV3** | MAPK | P38 | - | - | GYAHRD | DFG and alphaC |
|  | **3HV4** | MAPK | P38 | Out | In | GYAH-D | DFG and alphaC |
|  | **3HV5** | MAPK | P38 | Out | In | GYAHRD | DFG and alphaC |
|  | **3HV6** | MAPK | P38 | - | - | GYAHRD | DFG and alphaC |
|  | **3HV7** | MAPK | P38 | - | In | -YAHRD | DFG and alphaC |
|  | **3IW8** | MAPK | P38 | - | - | GYAHRD | DFG and alphaC |
|  | **3L8S** | MAPK | P38 | Out | In | GYAH-D | HINGE |
|  | **3LFB** | MAPK | P38 | - | In | -YAHRD | DFG and alphaC |
|  | **3LFC** | MAPK | P38 | - | In | -YA-RD | DFG and alphaC |
|  | **3LFD** | MAPK | P38 | - | In | GYA-RD | DFG and alphaC |
|  | **3LFE** | MAPK | P38 | - | In | GYAHRD | DFG and alphaC |
|  | **3LFF** | MAPK | P38 | - | In | -YAHRD | DFG and alphaC |
|  | **3NNU** | MAPK | P38 | - | In | GYAHRD | DFG and alphaC |
|  | **3NNV** | MAPK | P38 | - | In | -YAHRD | DFG and alphaC |
|  | **3NNW** | MAPK | P38 | - | In | GYAHRD | DFG and alphaC |
|  | **3NNX** | MAPK | P38 | - | In | GYAHRD | DFG and alphaC |
|  | **3NPC** | MAPK | P38 | - | In | GYAHRD | DFG and alphaC |
|  | **3NWW** | MAPK | P38 | - | In | GYAHRD | DFG and alphaC |
|  | **3O8P** | MAPK | P38 | Out | - | GYAHRD | DFG and alphaC |
|  | **3O8T** | MAPK | P38 | - | In | GYAHRD | DFG and alphaC |
|  | **3O8U** | MAPK | P38 | - | In | -YAHRD | DFG and alphaC |
|  | **3OBJ** | MAPK | P38 | - | - | -YA-RD | DFG and alphaC |
|  | **3OC1** | MAPK | P38 | - | In | -YAHRD | DFG and alphaC |
|  | **3P5K** | MAPK | P38 | - | In | GYA-RD | DFG and alphaC |
|  | **3P78** | MAPK | P38 | - | In | GYAHRD | DFG and alphaC |
|  | **3P79** | MAPK | P38 | - | In | GYAHRD | DFG and alphaC |
|  | **3P7A** | MAPK | P38 | - | In | GYAHRD | DFG and alphaC |
|  | **3P7B** | MAPK | P38 | - | In | GYAHRD | DFG and alphaC |
|  | **3P7C** | MAPK | P38 | - | In | GYAHRD | DFG and alphaC |
|  | **3PG3** | MAPK | P38 | - | - | GYAHRD | DFG and alphaC |
| **OPK** | **2W1E** | AUR | AUR | In | - | GY-HRD | Hyd1 |
|  | **2W1G** | AUR | AUR | In | - | GY-HR- | Hyd1 |
|  | **3D14** | AUR | AUR | In | - | GYAHRD | Hyd1 |
|  | **3D15** | AUR | AUR | In | - | GYAHRD | Hyd1 |
|  | **3D2I** | AUR | AUR | In | - | GYAHRD | Hyd1 |
|  | **3D2K** | AUR | AUR | In | - | GYAHRD | Hyd1 |
|  | **3DJ7** | AUR | AUR | In | - | GYAHRD | Hyd1 |
|  | **3EFW** | AUR | AUR | - | - | GYAHRD | Hyd1 |
|  | **3M11** | AUR | AUR | In | - | GYAHRD | Hyd1 |
|  | **3DB6** | PLK | PLK | In | - | GYAHRD | Hyd1 and alphaC |
| **TK** | **2HZN** | ABL | ABL | Out | In | GYAHRD | DFG and alphaC |
|  | **3QRI** | ABL | ABL | Out | In | GYAHRD | DFG and alphaC |
|  | **3QRJ** | ABL | ABL | Out | In | GYAHRD | DFG and alphaC |
|  | **3QRK** | ABL | ABL | Out | In | -YA-RD | DFG and alphaC |
|  | **3FZS** | FAK | FAK | Out | In | GYAHRD | DFG and alphaC |
|  | **3FZT** | FAK | FAK | Out | In | GYAHRD | DFG and alphaC |
|  | **2FGI** | FGFR | FGFR | - | In | GY-HRD |  |
|  | **3ETA** | INSR | INSR | Out | In | GYAHRD | DFG and alphaC |
|  | **2W1I** | JAKA | JAKA | In | In | GY-HRD |  |
|  | **3C1X** | MET | MET | - | - | GYAHRD | DFG and alphaC |
|  | **3CTH** | MET | MET | Out | In | GYAHRD | DFG and alphaC |
|  | **3CTJ** | MET | MET | Out | In | GYAHRD | DFG and alphaC |
|  | **3EL7** | SRC | SRCA | - | In | GYAHRD | DFG and alphaC |
|  | **3EL8** | SRC | SRCA | Out | In | GYAHRD | DFG and alphaC |
|  | **3F3T** | SRC | SRCA | Out | In | GYAHRD | DFG and alphaC |
|  | **3F3U** | SRC | SRCA | Out | In | GY-HRD |  |
|  | **3F3V** | SRC | SRCA | Out | In | GYAHRD | DFG and alphaC |
|  | **3F3W** | SRC | SRCA | Out | In | GYAHRD | DFG and alphaC |
|  | **1YWN** | VEGFR | VEGFR | - | In | GYAHRD | DFG and alphaC |
|  | **2OH4** | VEGFR | VEGFR | Out | In | GYAHRD | DFG and alphaC |
|  | **3VHE** | VEGFR | VEGFR | Out | In | GYA-RD | DFG and alphaC |
| **TKL** | **1UWH** | RAF | RAF | Out | In | GYAHRD | DFG and alphaC |
|  | **1UWJ** | RAF | RAF | Out | In | GYAHRD | DFG and alphaC |

**Regarding the table:**

1-Binding is annotated using GYAHRD which is short for G-loop, Hyd1, alphaC, hinge, HRD and DFG motifs respectively. They can be defined as follows:

| **Region** | **Residues** | **Description** |
| --- | --- | --- |
| **G-Loop** | *GxGxxGxxV* | Important component for ligand binding. The invariant conserved Glycines allow a close approach of the nucleotide to the peptide backbone. |
| **Hyd1** | *Lys72* | Residues within this region are in hydrophobic contact with the adenine ring of ATP forming a hydrophobic pocket. The invariant Lys72 residue interacts with β-phosphoryl groups of the bound ATP and is thought to be crucial in mediating correct orientation of the ligand. |
| **alphaC** | *Glu91* | The spacial position of Lys72 is secured by a salt-bridge with Glu91 in the center of the only helix in the C-lobe of the enzyme. This helix is thought to be important for enzyme regulation since the activation of kinases is often accompanied by changes in it's orientation. |
| **Hinge** |  | Linker region between the N and C-lobe. The adenine ring of ATP forms hydrogen bonds with backbone atoms of the hinge residues. The residue N-terminal to these H-Bond contacts is termed the *Gatekeeper* and controls access to the back cleft. The C-terminal residues of the hinge region form in concert with the G-Loop the ribose binding pocket. |
| **HRD** | *Asp166 Lys168 Asn171* | Catalytic center with the characteristic sequence motif *HRDxxxxN* that facilitates phosphoryl-transfer to substrate. Lys168 forms direct contact to the γ-phosphoryl group of ATP, neutralizes the negative charge and stabilizes the intermediate state. Asp166 represents the catalytic base that abstracts the proton from the hydroxyl-group of the substrate, Asn171 coordinates with the second Mg^2+^-ion (Mg2) that chelates the α- and γ-phosphate oxygens. |
| **DFG** | *Asp184 Phe185* | This motif is located at the base of the kinase activation segment. The carboxyl-group of Asp184 is in complex with the chelating Mg^2+^-ion (Mg1) which coordinates β- and γ-phosphoryl oxygens forming almost an optimal octahedral coordinate scheme. The orientation of Phe185 residue has in many kinases been shown to contribute to stabilizing the active site pocket by forming hydrophobic contacts with residues from the αC-helix and the nearby HRD motif (hydrophobic spine). |

2- Regarding the DFG and alphaC annotations:

**DFG** Specify the DFG conformation state (*DFG-out* or *DFG-in*) of the kinases.

**alphaC** Specify the interaction state (*alphaC-out* or *alphaC-in*) of the highly conserved Lys72 with Glu91 in the center of αC-helix. In the *in* conformation, Glu91 forms an ionic interaction with Lys72.

3- The complexes analysis was based on color codes which designate the different regions of the binding site. These color codes are listed below:


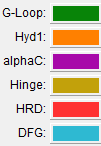


4-Regarding the role of these regions in ATP binding:


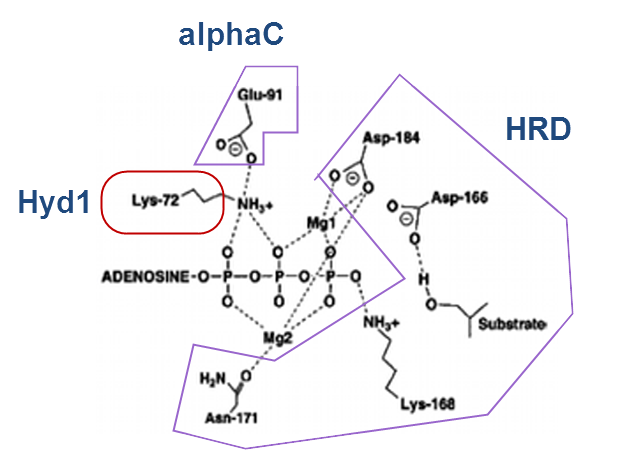

Supplement: Text S1 — Urea-derivatives kinases complexes used to generate field templates. (DOCX) [file pone.0049284.s001.docx]
