# Supplementary material for: Applying Ligands Profiling Using Multiple Extended Electron Distribution Based Field Templates and Feature Trees Similarity Searching in the Discovery of New Generation of Urea-Based Antineoplastic Kinase Inhibitors
Source: PLoS One. 2012 Nov 20;7(11):e49284. doi: 10.1371/journal.pone.0049284 (PMC3502486; doi:10.1371/journal.pone.0049284)

**Color codes used to designate field templates**


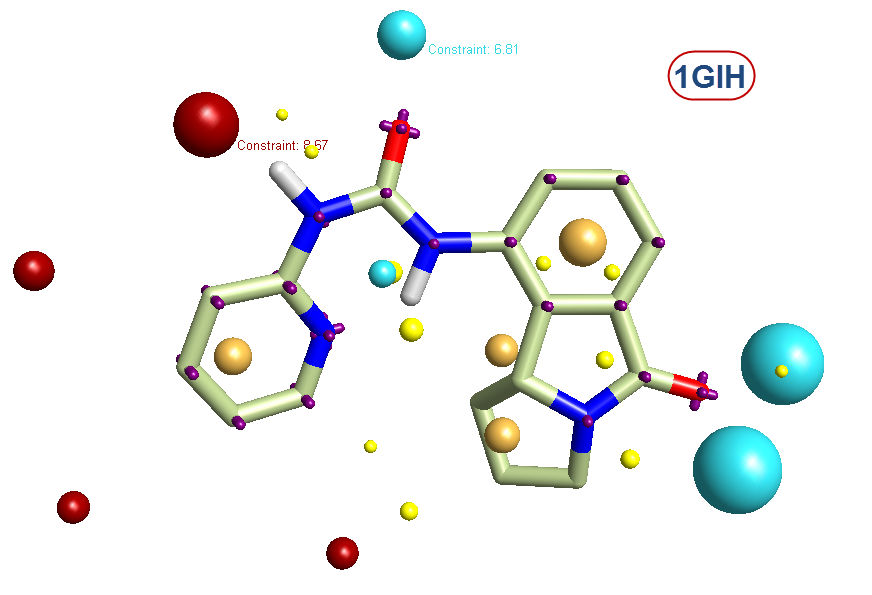


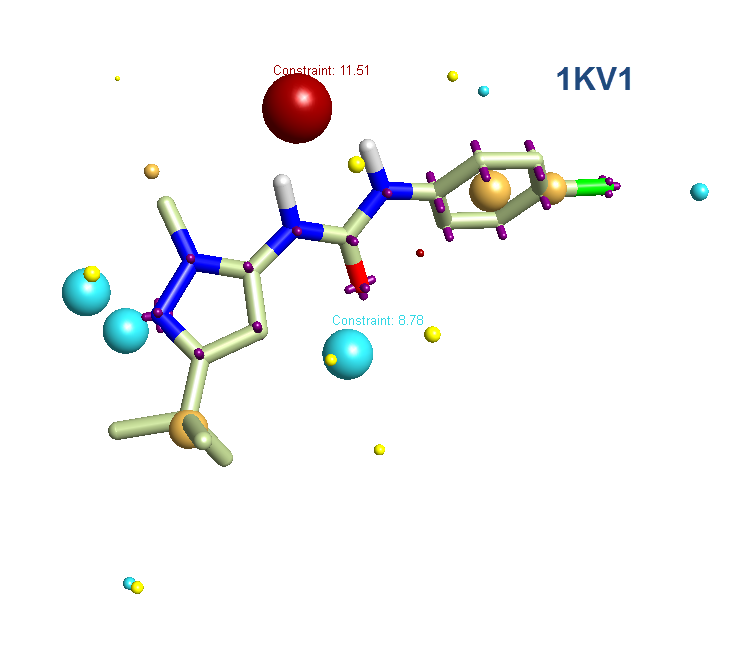


1GIH represents human cyclin-dependent kinase 2 complexed with the cdk4 inhibitor while 1KV1 represents p38 map kinase in complex with inhibitor.

The field templates generated for these two complexes are color coded according to this key:


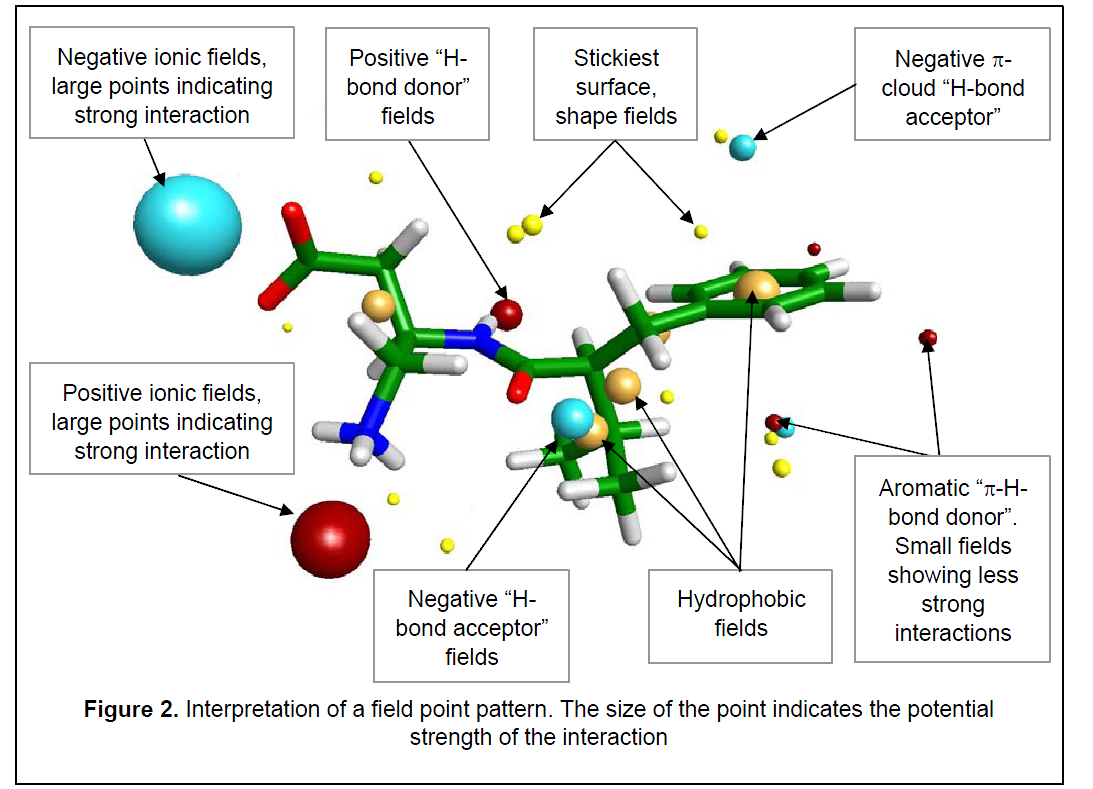

Supplement: Text S2 — Colour codes used to designate field templates. (DOCX) [file pone.0049284.s002.docx]
