# Supplementary material for: Applying Ligands Profiling Using Multiple Extended Electron Distribution Based Field Templates and Feature Trees Similarity Searching in the Discovery of New Generation of Urea-Based Antineoplastic Kinase Inhibitors
Source: PLoS One. 2012 Nov 20;7(11):e49284. doi: 10.1371/journal.pone.0049284 (PMC3502486; doi:10.1371/journal.pone.0049284)
Supplement: Text S3 — SVM (Support vector machine) model. (DOCX) [file pone.0049284.s003.docx]

**SVM (Support vector machine) model**

The ligands were extracted from the urea derivatives kinase complexes and duplicates were removed to finally give 73 diverse ligand.

A set of decoys (1247 urea-based ligands) extracted from zinc database was used in this model. Initially the urea-based compounds were extracted from zinc database using substructure searching which depends on SMILES Arbitrary Target Specification (SMARTS) pattern. After that, the decoys were selected from them such that each active ligand of the 73 has 36 corresponding decoys. The criteria of selection were as following: (i) molecular weight of ±40 Da; (ii) same number of rotational bonds, HBDs (Hydrogen bond donors), and HBAs (Hydrogen bond acceptors); (iii) cLogP of ±1.0 and (iv) TPSA ±15[[1](#_ENREF_1)]. However, this was not feasible in some cases so in case of failure of meeting these strict requirements, the conditions were modified slightly to allow finding proper solutions. Mostly, the conditions were modified to allow ±1 in the number of rotational bonds, HBDs (Hydrogen bond donors), and HBAs (Hydrogen bond acceptors). Finally, the redundant compounds were removed to form a set of 1247 urea-based decoys.

SVM was carried out as following:

R SVM function implemented in Accelrys pipeline pilot 8 was used to build a model for kinase activity. The descriptor used belongs to the Extended-Connectivity Fingerprints (ECFPs) class. ECFPs are well suited to the recognition of the presence or absence of particular substructures. It is known that the three main parameters of ECFPs are maximum diameter, fingerprint length, and identifier counts. Herein, we used ECFP_4 which denotes that the maximum diameter is set to 4 (http://www.chemaxon.com/jchem/doc/user/ECFP.html).We consider compounds either active (73 compounds) or inactive (decoys).

We split the data in training and test set (50/50), and save the test set for validation later. Internal validation was carried using a 5-fold cross validation on the training data. This is clear as shown in the figure.


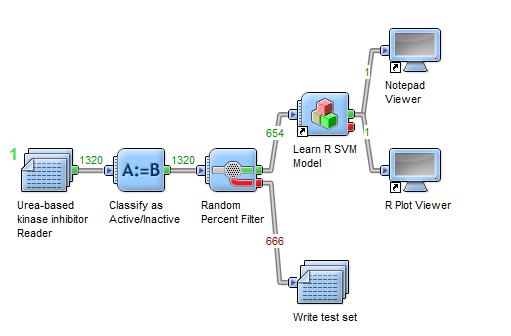


The result was quickly examined using the R-plot viewer which showed an excellent 5-fold cross validation ROC score of 0.98 as shown in the following diagram:


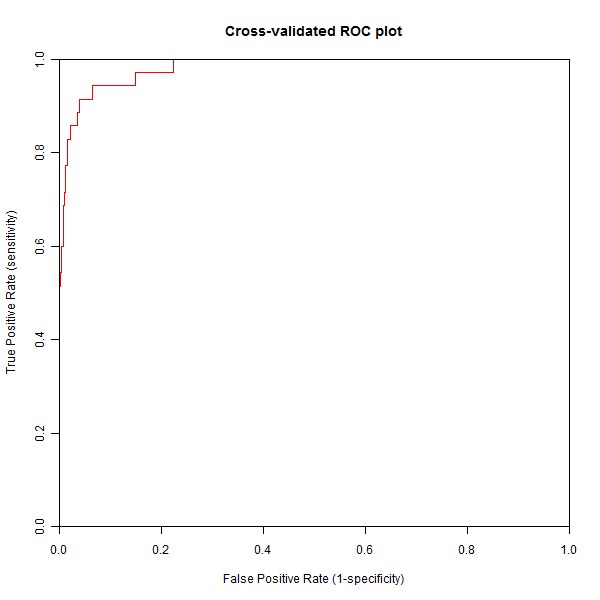


The test set was used for external validation using the following pipeline protocol which creates an enrichment plot, ROC plot, and computes an overall ROC score.


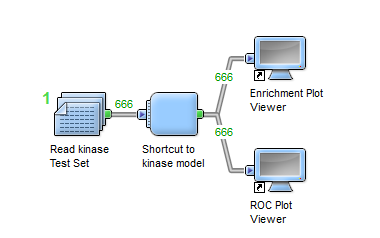


The enrichment plot is given in the following figure:


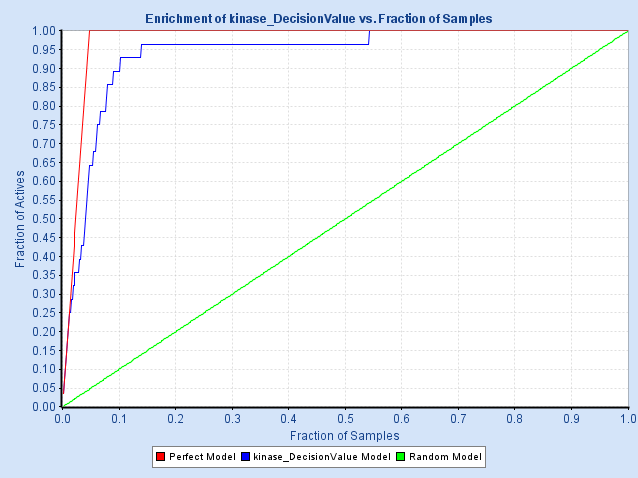


The plot results from applying the learned model to the set of test data with known good and bad values. Internally, the data records are sorted by the learned model score from highest to lowest. The X-axis of the plot contains the fraction of records seen following the sort. The Y-axis contains the fraction of good records found in the sorted list up to that point. The plot lets you see how well the model assigns higher scores to known good records than to known bad records.

In addition to the enrichment plot for the model, plots for two hypothetical models are shown as limiting cases. For a "perfect model," every good record appears in the sorted list before every bad record. For a "random model," the fraction of good records seen is just the fraction of total records seen, meaning the random model makes no distinction at all between good and bad records.

The ROC plot is also given in the following figure:


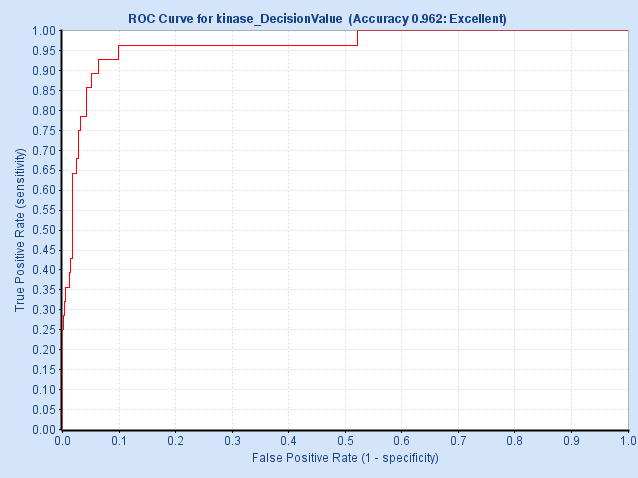


A ROC plot can help in understanding the tradeoff between model sensitivity (the model's ability to identify true positives) and model specificity (its ability to avoid false positives) in a learned model.

As shown, the ROC score -- the area under the curve of the ROC plot- is reported in the plot title. The score is a number between 0.5 and 1.0. The closer the ROC score is to 1.0, the better the model is at distinguishing good from bad samples. It is clear that the model score is excellent.

1. Irwin JJ (2008) Community benchmarks for virtual screening. J Comput Aided Mol Des 22: 193-199.
