# Supplementary material for: Applying Ligands Profiling Using Multiple Extended Electron Distribution Based Field Templates and Feature Trees Similarity Searching in the Discovery of New Generation of Urea-Based Antineoplastic Kinase Inhibitors
Source: PLoS One. 2012 Nov 20;7(11):e49284. doi: 10.1371/journal.pone.0049284 (PMC3502486; doi:10.1371/journal.pone.0049284)
Supplement: Text S4 — Bayesian model. (DOCX) [file pone.0049284.s004.docx]

**Bayesian model**

The model was constructed by the same dataset used in the model above.

The set was divided into training and test sets. The only difference is that we used a 3D pharmacophore-based fingerprint that is built using 3 points. The features used were H-bond acceptor feature, H-bond donor feature and hydrophobic feature.

The descriptors (just like Tuplets in Tripos Sybyl) are pharmacophore distance multiplets, created from known ligands.

The full pharmacophoric pattern found for each ligand is decomposed into its constituent two, three, or four feature pharmacophore distance multiplets and is encoded into a fingerprint. This was done for the single bioactive conformer (extracted from the complex) of the ligand molecule.

The dataset including the 73 active compounds and the 1247 decoys were divided into training and test sets according to the protocol of generating training and test set in Accelrys Discovery studio 3.0. They were divided on 50/50 basis according to a defined set of descriptors: AlogP, Molecular_Weight, Num_H_Donors, Num_H_Acceptors, Num_RotatableBonds, Num_Atoms, Num_Rings, Num_AromaticRings, Num_Fragments, Molecular_PolarSurfaceArea. The splitting method used was random in which the full ligand set is sorted by a random index, the "Top N" ligands are then assigned to the training set based on the value of the Training Set Percentage parameter.

The results of the internal validation using the 5-fold cross validation and the external validation using ROC plot are illustrated as following:


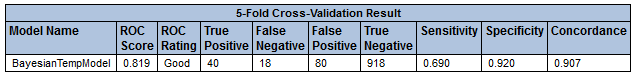


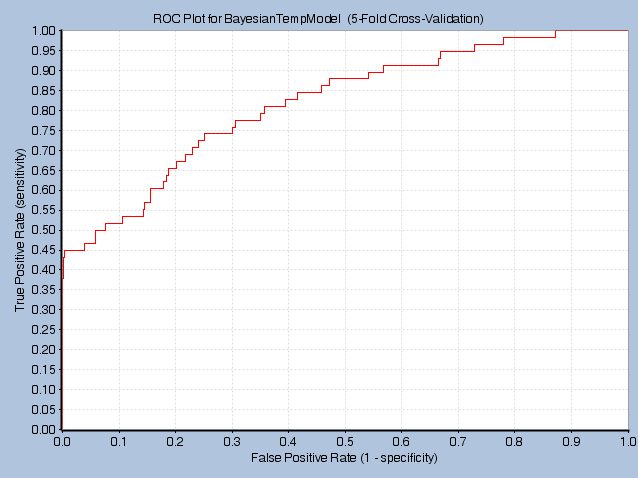


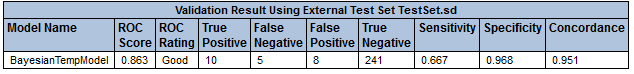


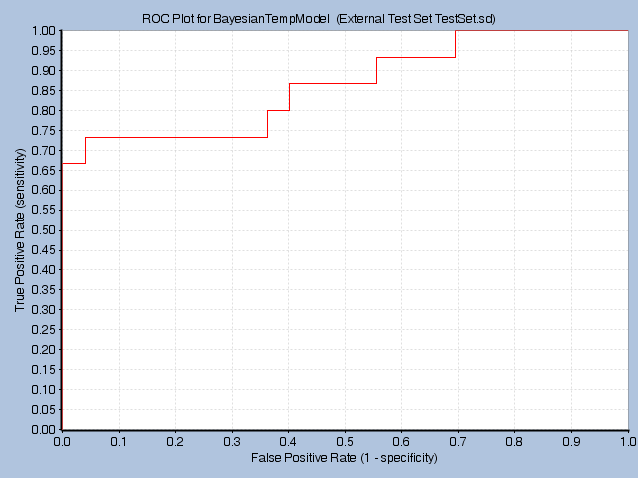


The hits retrieved from both models were merged and followed by duplicates removal.
