# Supplementary material for: Applying Ligands Profiling Using Multiple Extended Electron Distribution Based Field Templates and Feature Trees Similarity Searching in the Discovery of New Generation of Urea-Based Antineoplastic Kinase Inhibitors
Source: PLoS One. 2012 Nov 20;7(11):e49284. doi: 10.1371/journal.pone.0049284 (PMC3502486; doi:10.1371/journal.pone.0049284)
Supplement: Text S5 — Structure-based pharmacophores. (DOCX) [file pone.0049284.s005.docx]

**Structure-based pharmacophores**

This technique was developed by Wolber and Langer as a good technique for screening of new compounds instead of computationally expensive docking.[^1^](#_ENREF_1) The technique was implemented already in ligandscout software.[^2^](#_ENREF_2) This algorithm extracts information according to certain rules depending on nearby contact residues.

As we pointed out in the complex analysis, the urea fragment can either bind to Hinge, DFG or Hyd1 regions or may not bind to these regions at all. Thus, we modified any created pharmacophore and added a custom urea-fragment feature (created using catalyst) to guarantee the correct positional mapping of this feature. The custom urea-fragment feature is illustrated below:


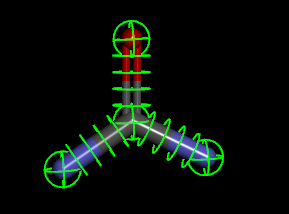


The pharmacophores were created for each group as follows:

***AGC group***


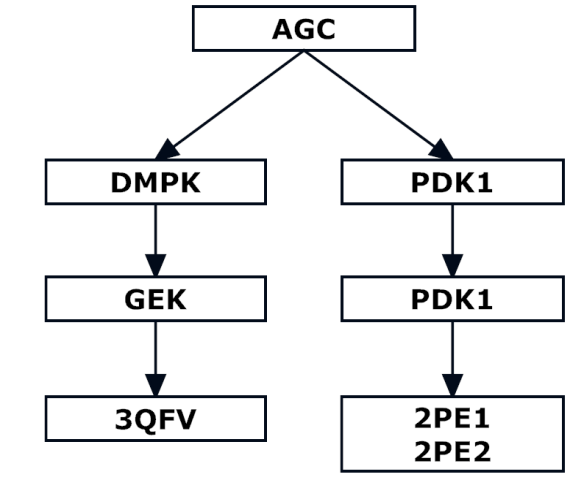


2 pharmacophores were created; one for 3QFV while the other for 2PE1 and 2PE2 as follows:

For 3QFV, it is very important to note that both the amide linkage and the urea bind with hinge region.


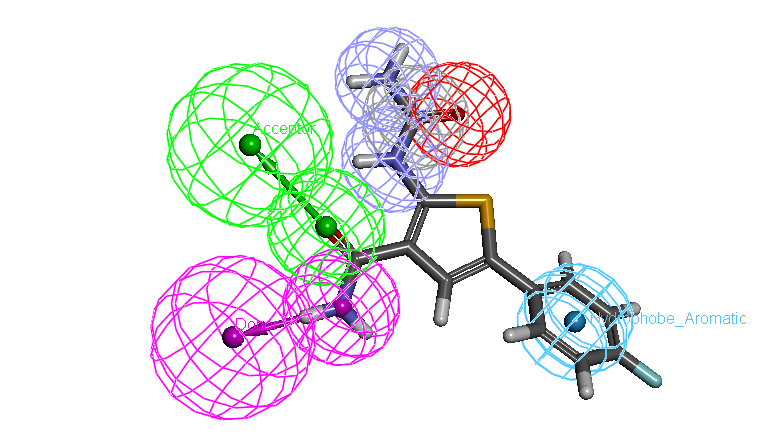


This can be deduced from the following 3D-interaction where the yellow loop is the hinge region.


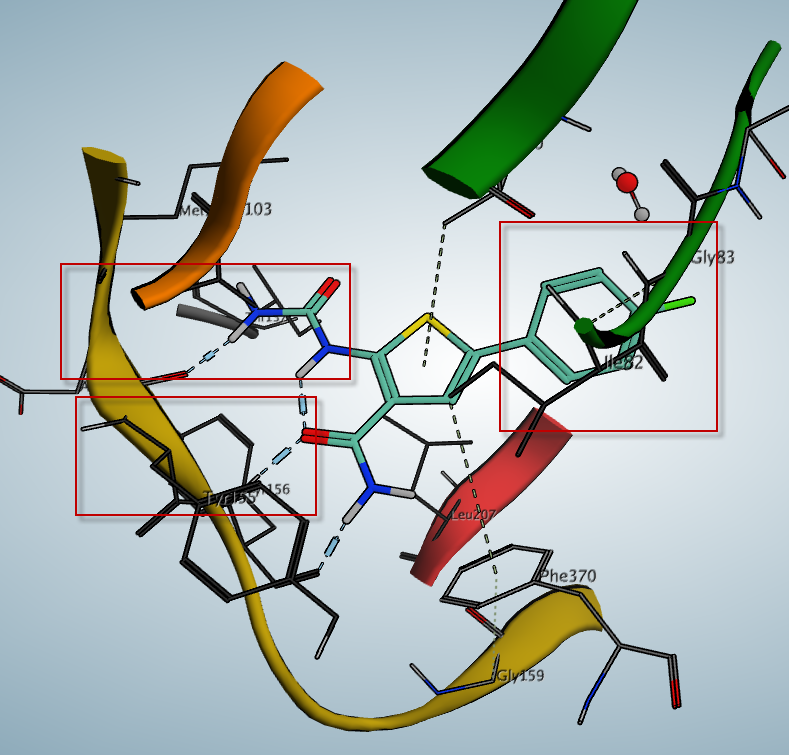


For the PDK1 Subfamily, the structure-based pharmacophore is illustrated below:


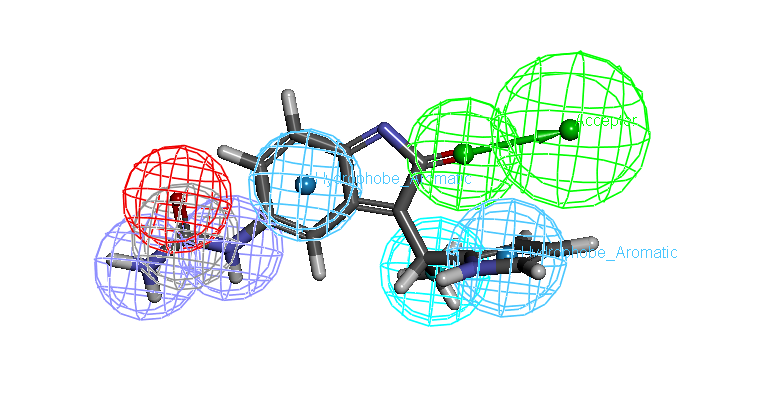


It is very important to know that the urea binds to the DFG region while the carbonyl oxygen binds to Hinge region.

***CAMK group***

Normally, urea-based inhibitors bind to the DFG region but in the CAMK group, it is observed that it binds to the hinge region.


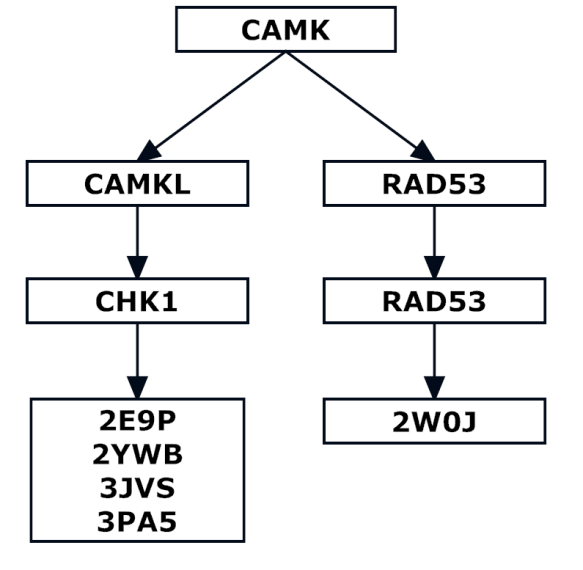


Out of these PDB codes, 3JVS was excluded as it binds to an allosteric site. Other than that, all the urea-based inhibitors in this class are hinge region binders.


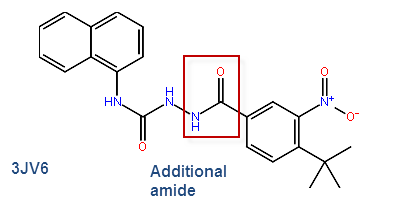


For 2E9P,
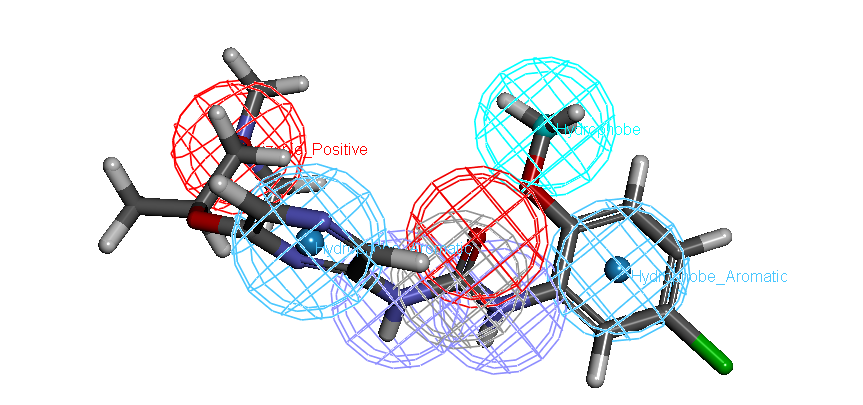


For 2YWP, the cyano group is a Hyd1 binder.


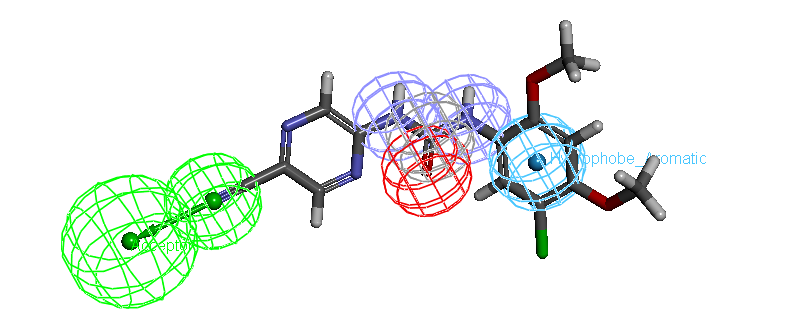


For 3PA5,


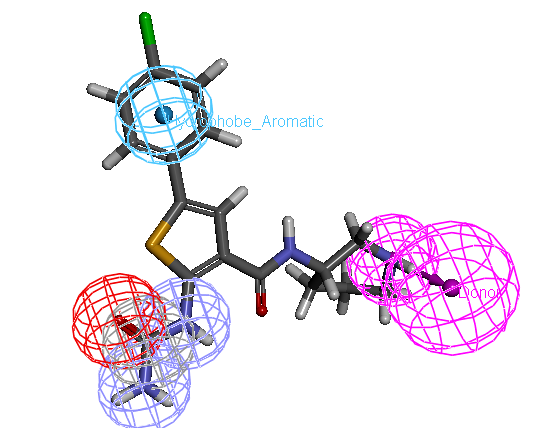

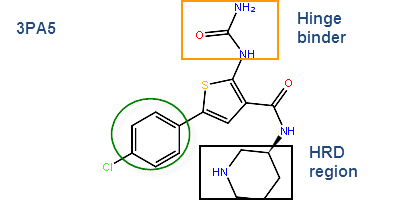


For 2W0J,


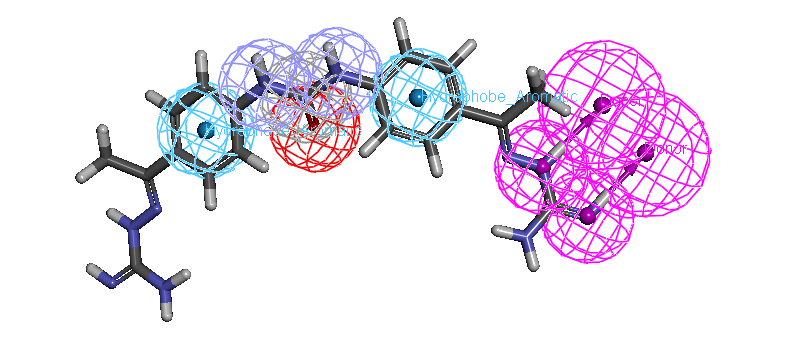


***CMGC group***


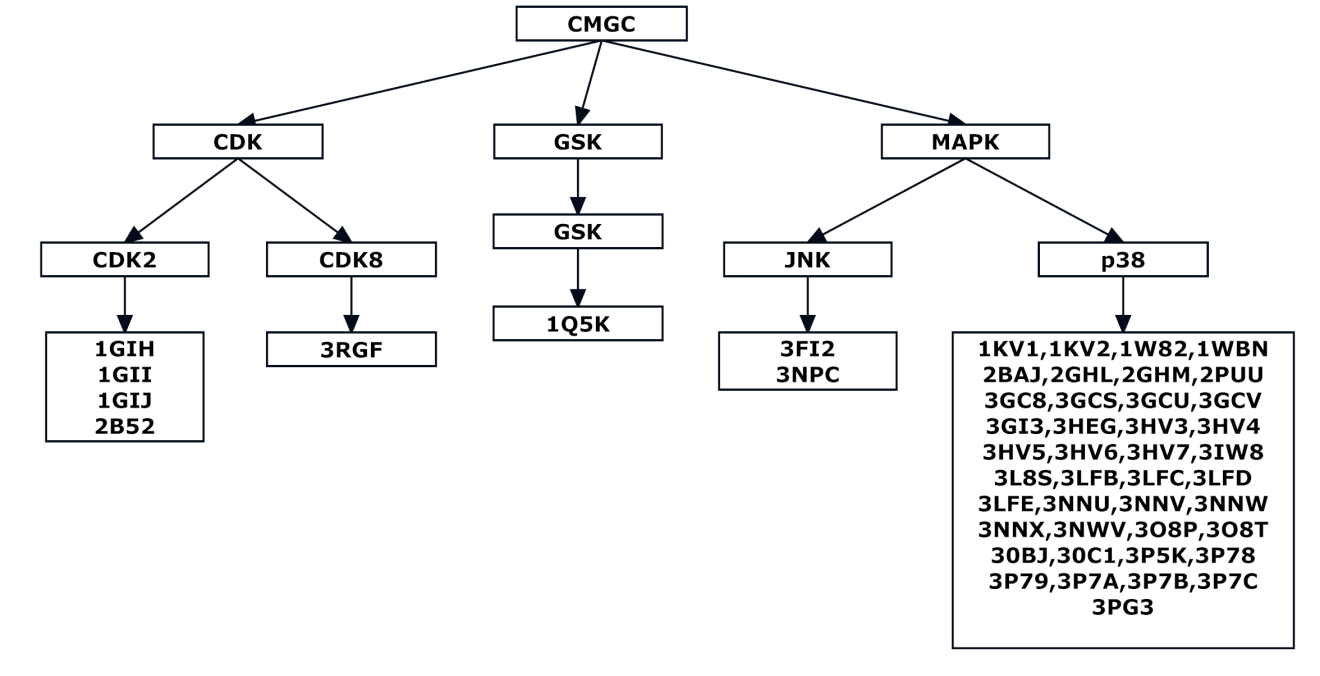


There are different pharmacophores which were used for this group.

We will begin with p38. There are two main major pharmacophores. The first one is based on 1W82 while the second is based on 1WBN.

For the one which is based on 1W82, the urea binds to the DFG and alpha-C region. However, it has no extension which can interact with Hinge region via H-bond.


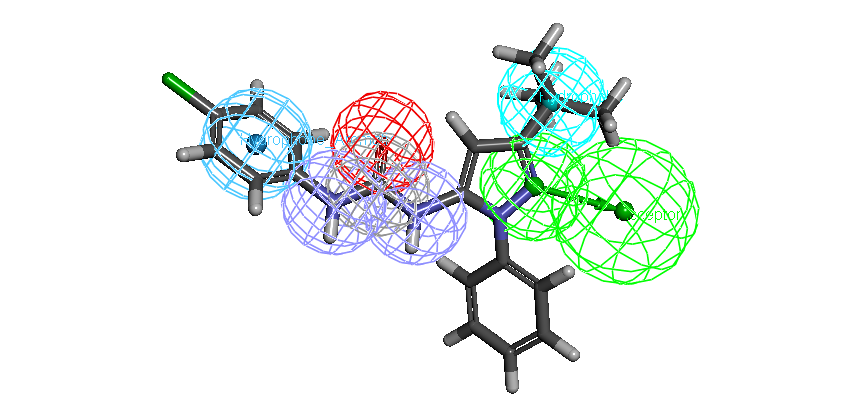


This pharmacophore maps with the ligands of the following complexes:


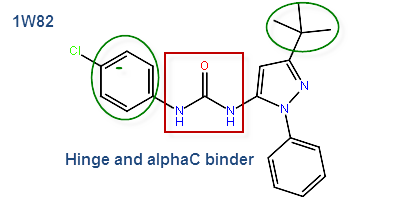

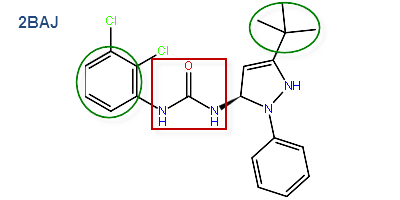


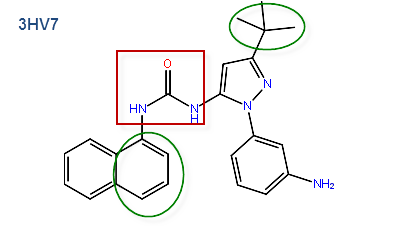

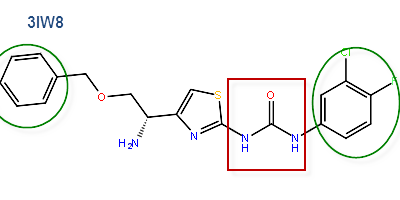


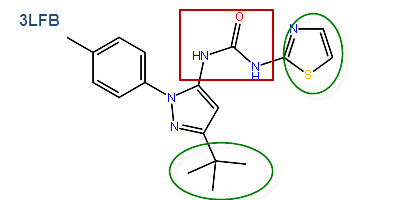

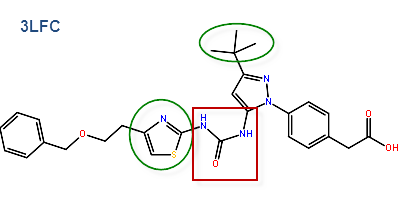


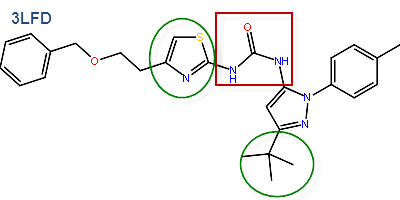

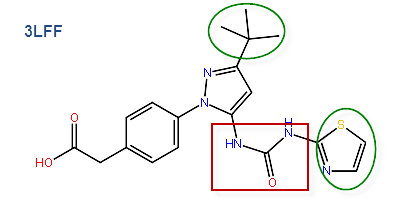


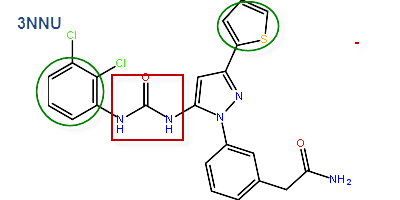

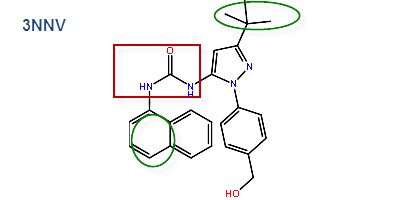


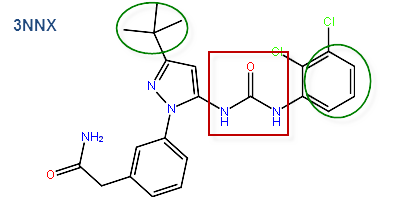

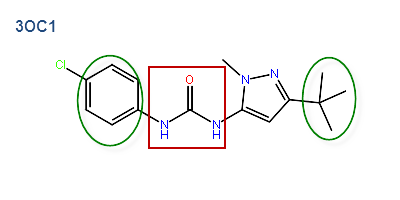


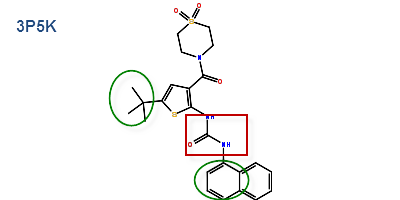

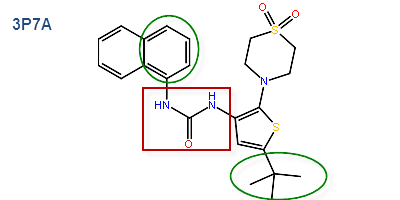


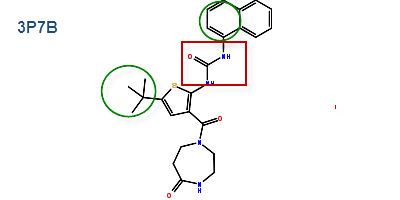

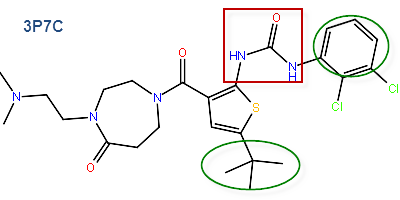


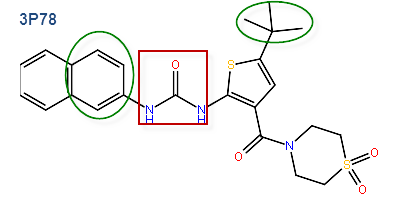

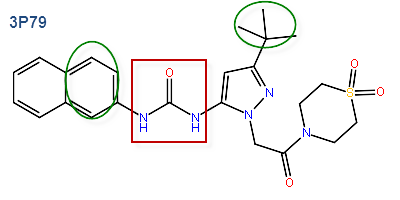


For the pharmacophore which is based on 1WBN, the urea binds to the DFG and alpha-C region. In addition it has extension which can form a hydrogen bond to the hinge region (shown in yellow).


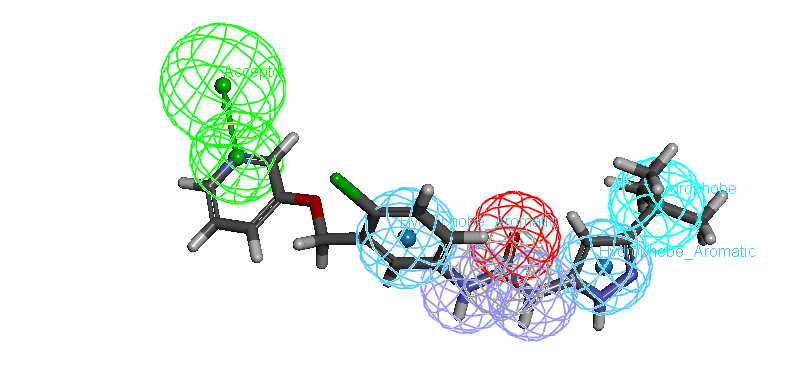


This pharmacophore maps with the ligands of the following complexes:

They are given in the following diagram:


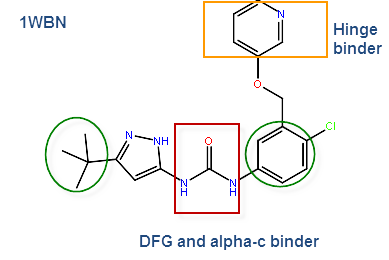

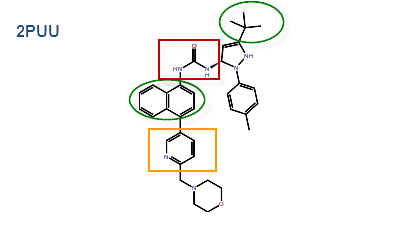

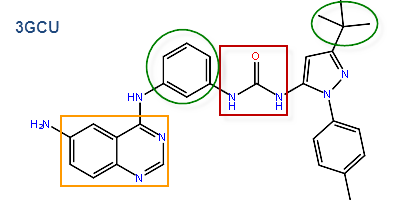

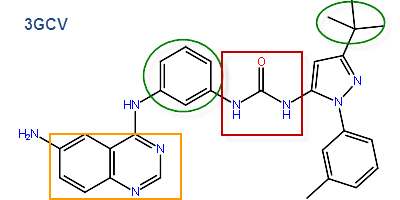


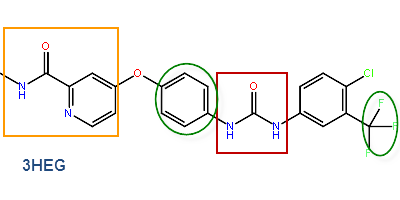

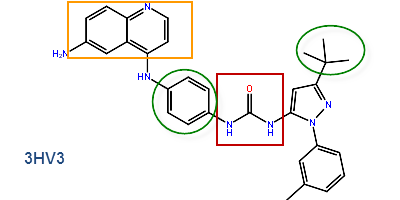


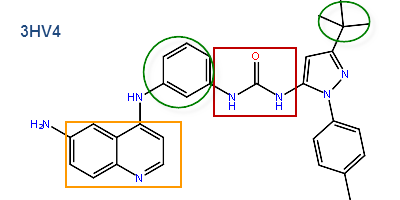

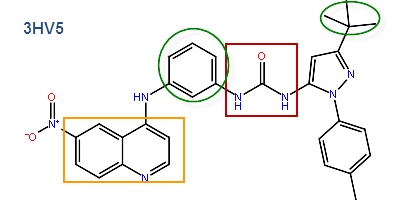


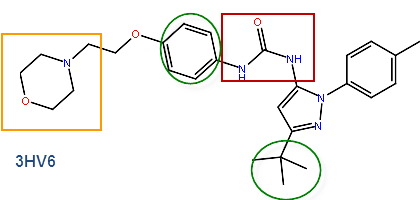

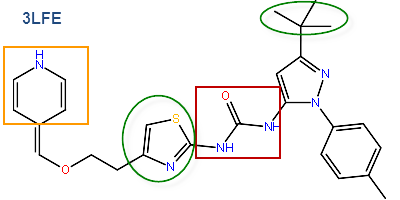


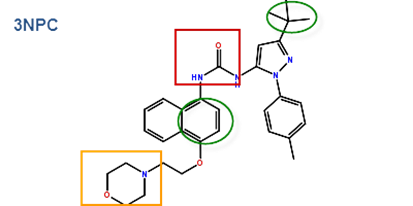

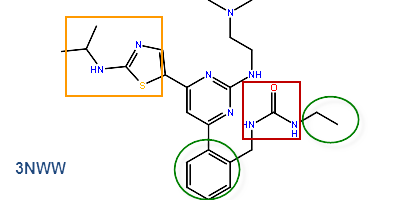


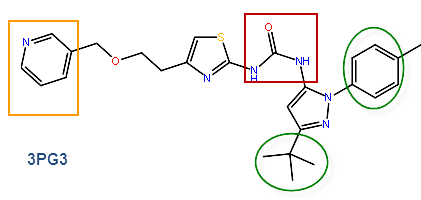


There were other pharmacophores for p38 which deviate from these two common pharmacophores. They are listed below:

Regarding 2GHL and 2GHM, the urea binds to the DFG and alpha-c region via Water Bridge while the amino-pyrimidine interacts with the Hinge region.


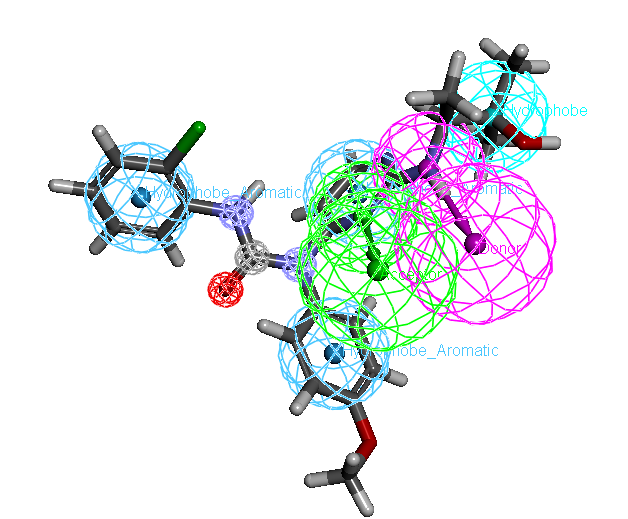


Regarding 3L8S, the amide linkage together with the urea fragment act as hinge region binder.


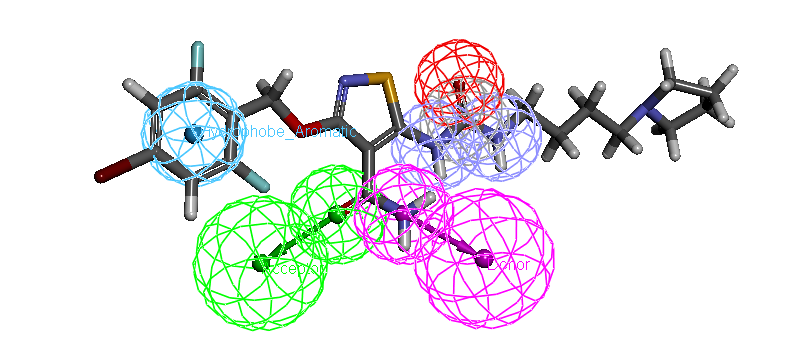


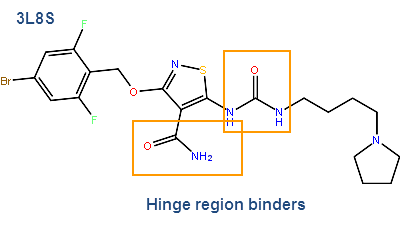


For the cdk2, we created a pharmacophore based on 1GIH and another one based on 2B52.

For 1GIH, the urea binds to the hinge region.


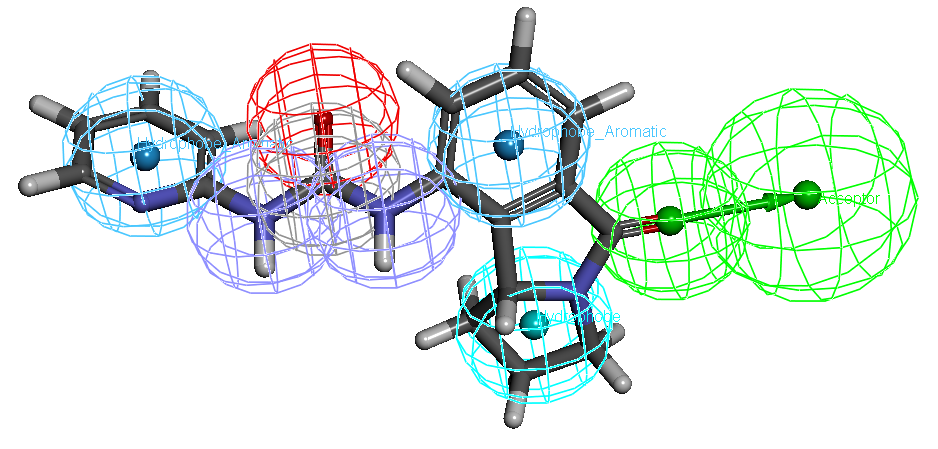


For 2B52, the urea binds to Hyd1 region.


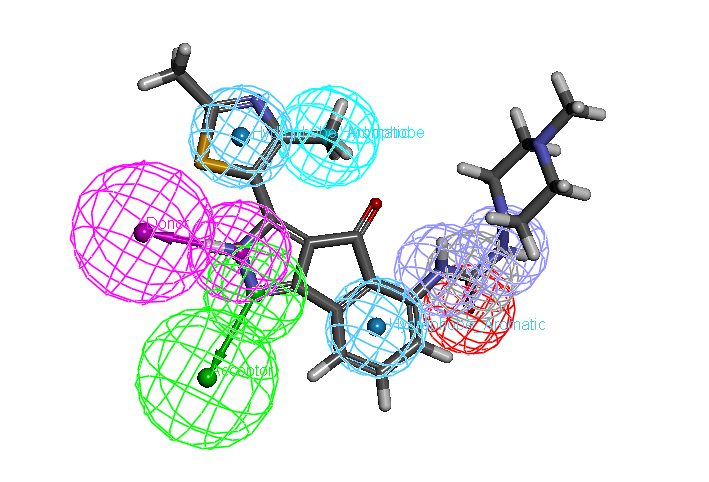


For GSK, the 1Q5K urea fragment binds to the hinge region.


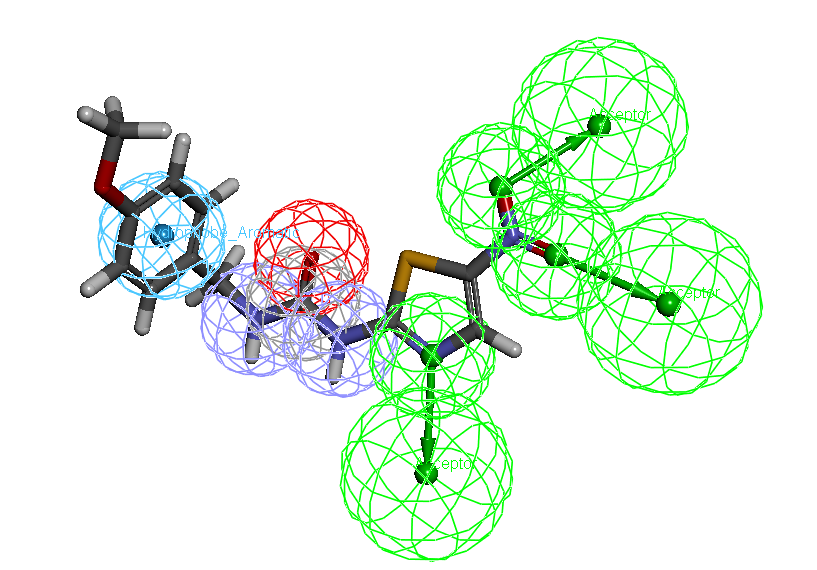


For JNK subfamily, the 3NPC maps with the pharmacophore based on 1WBN PDB code while 3FI2 pharmacophore is illustrated below. The pyrazole binds to the hinge region while urea binds to the Hyd1 region.


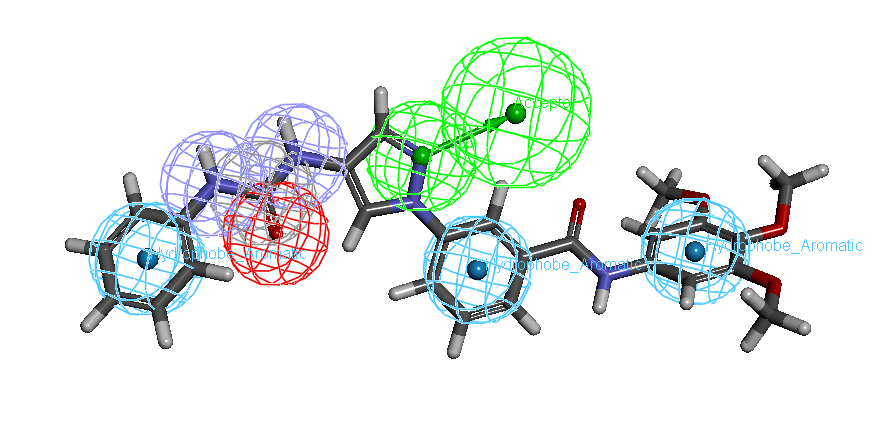


***OPK group***


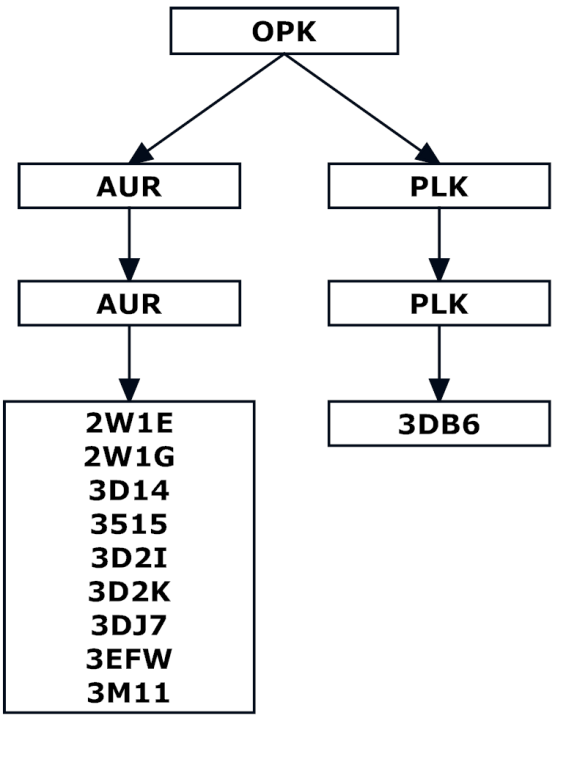


The urea here in the OPK group binds mostly to Hyd1 region.

2W1E and 2W1G shares a common pharmacophore. The others share another pharmacophore which is very similar to the pharmacophore of the 1WBN p38 map kinase.

Regarding 2W1E,


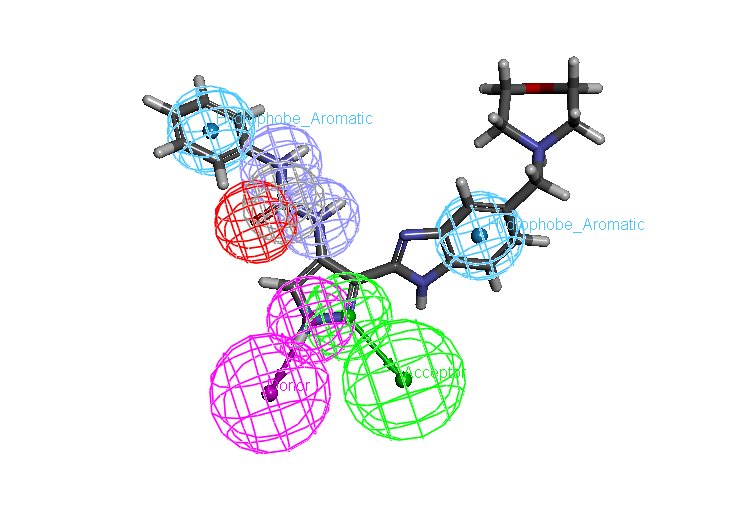

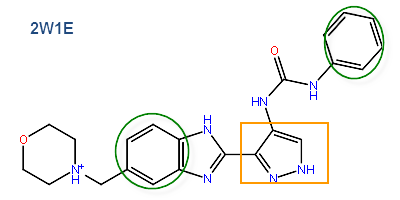


For the others, the pharmacophore is built using 3D2I where the urea binds to the Hyd1 region:


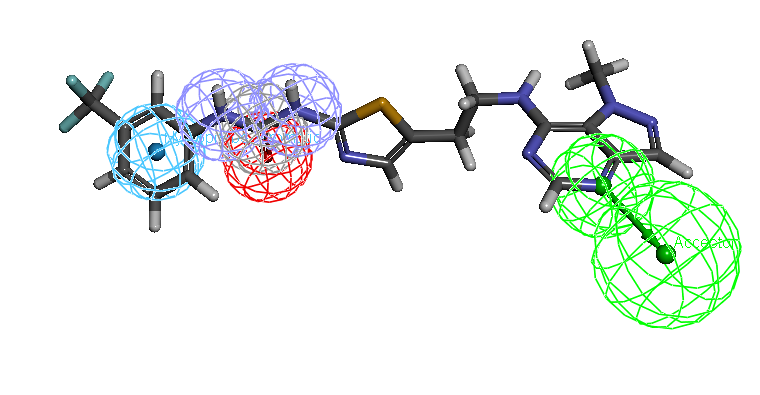


The inhibitors sharing the same pharmacophore are listed below:


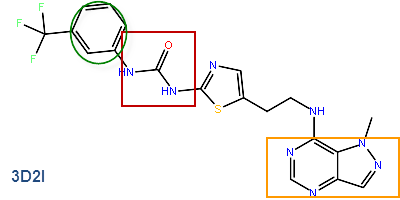

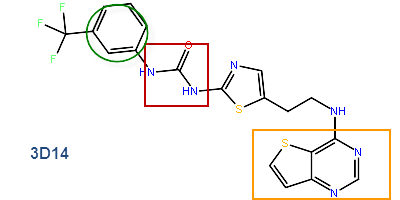


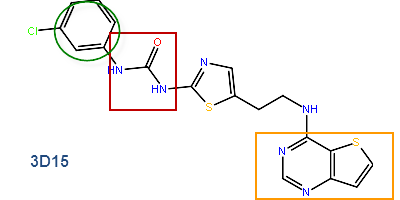

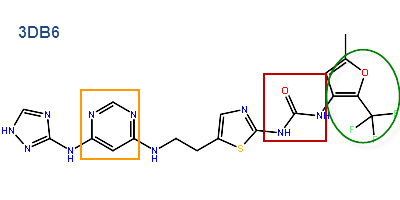


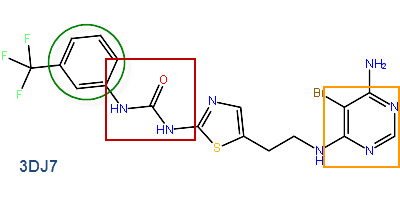

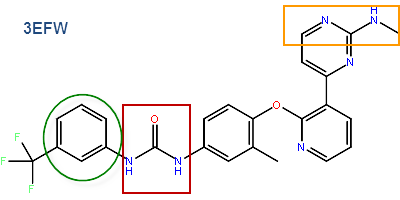


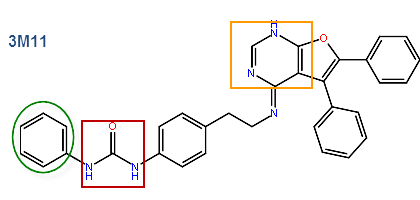


***TK and TKL groups***


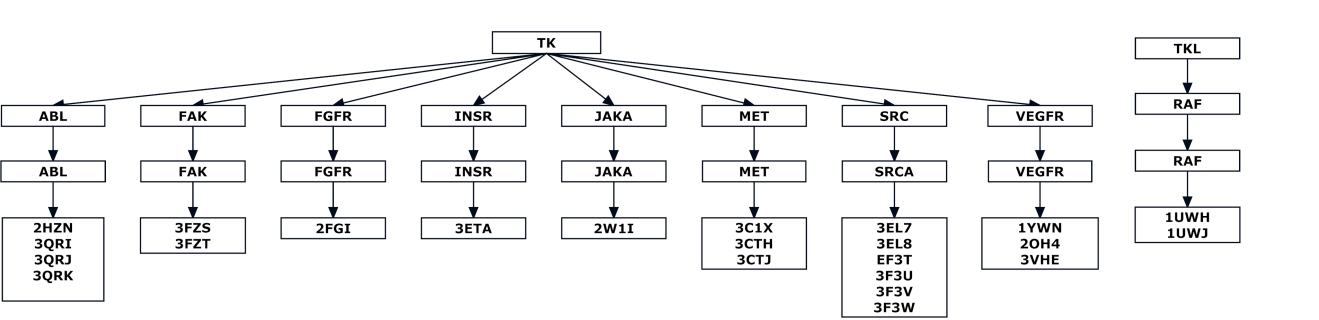


The general trend for the urea binding mode for these two groups is binding to the DFG and alpha-c regions.

One pharmacophore was built using 2HZN PDB code. It has an extension which can form hydrogen bond with the Hinge region.


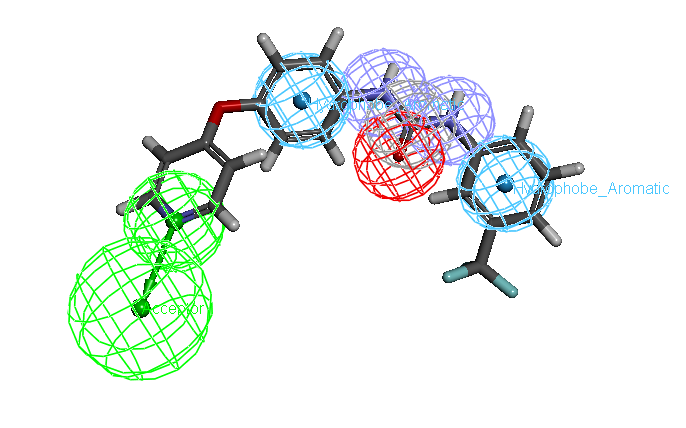


The following kinase inhibitors share the same pharmacophore:


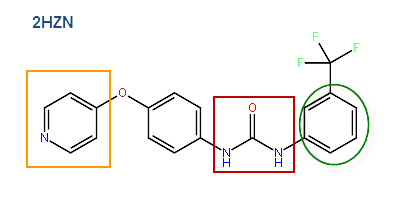

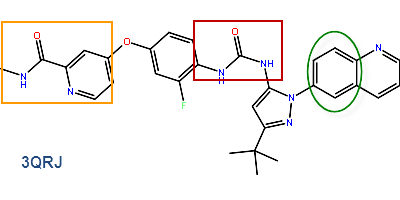


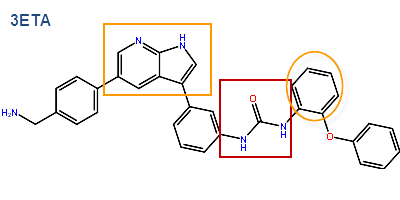

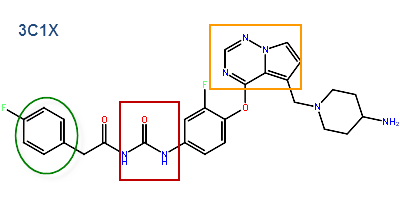


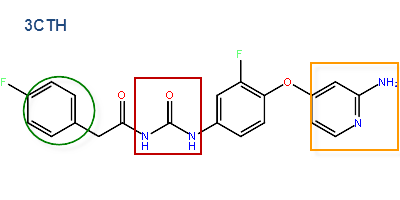

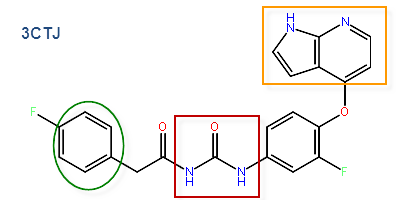


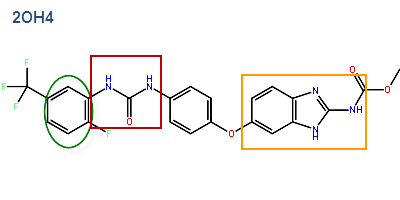

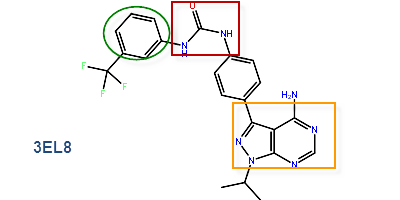


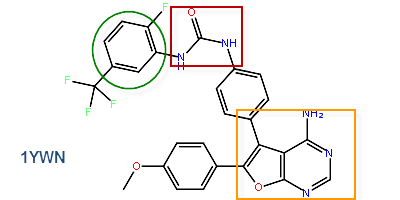

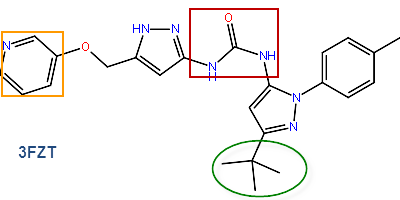


Another pharmacophore was built based on 3QRK


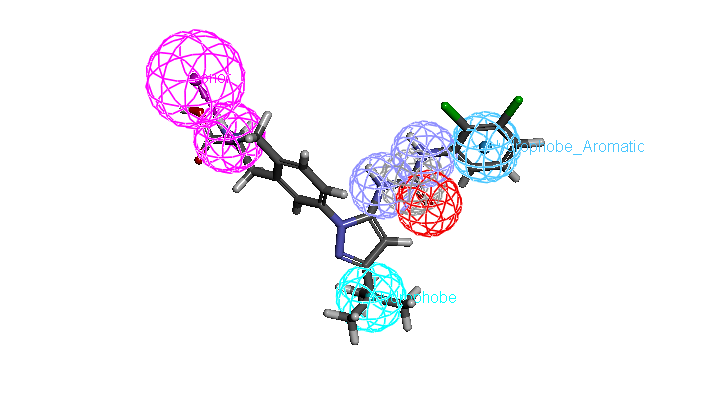

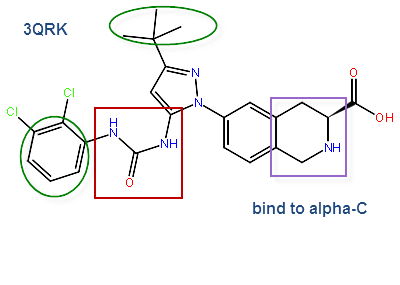


Another pharmacophore was based on 2FGI where the Nitrogen of the pyrido[2,3-d]pyrimidine is hydrogen bond acceptor that binds to the Hinge region.


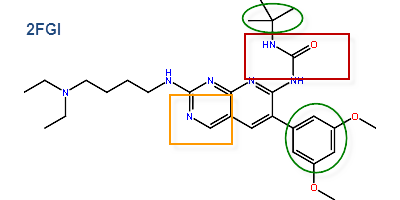

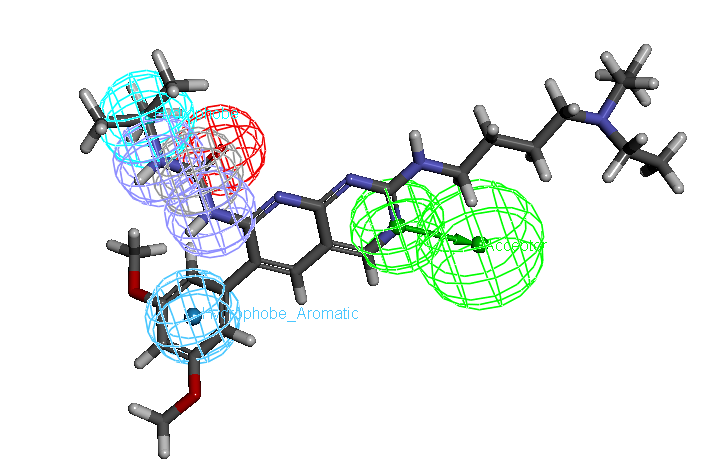


1. Wolber, G.; Langer, T. *Journal of Chemical Information and Modeling* **2005,** *45*, 160.

2. Boyd, S. *Chem World-Uk* **2006,** *3*, 69.
