# Supplementary material for: Applying Ligands Profiling Using Multiple Extended Electron Distribution Based Field Templates and Feature Trees Similarity Searching in the Discovery of New Generation of Urea-Based Antineoplastic Kinase Inhibitors
Source: PLoS One. 2012 Nov 20;7(11):e49284. doi: 10.1371/journal.pone.0049284 (PMC3502486; doi:10.1371/journal.pone.0049284)
Supplement: Text S6 — Molecular modeling implementation results. (DOCX) [file pone.0049284.s006.docx]

**Molecular modeling implementation results**

The screening followed the workflow depicted below:


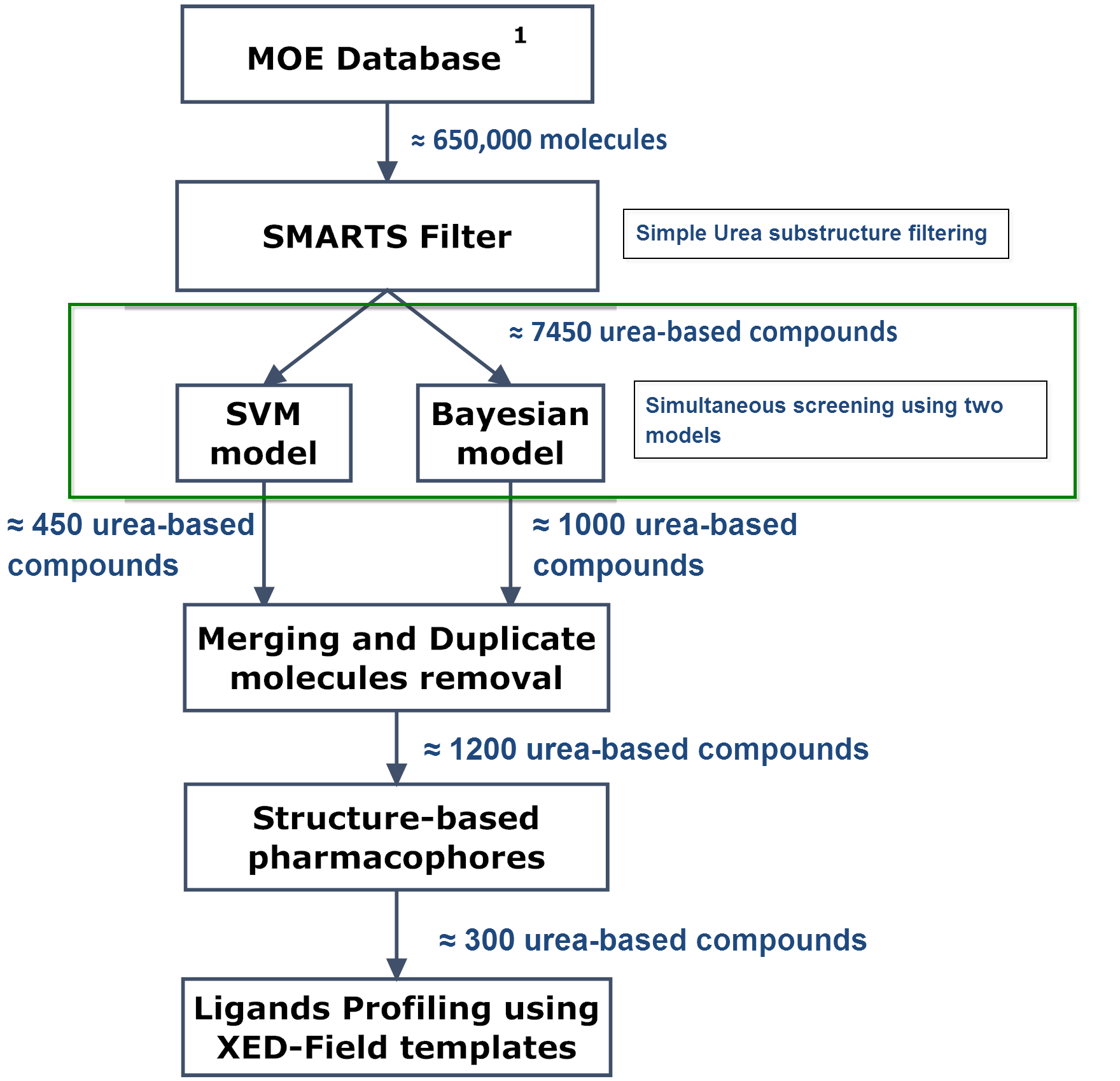


The database^1^ deployed in this screening was the one included in the MOE 2010 package representing the following commercial screening databases:

| A-Synthese-Biotech ASDI Akos Art-Chem Asinex Aurora Biofocus Bionet Biotech Corp. of America Cerep Chem T&I | Chembridge Chemdiv Chemical Block Chemstar Comgenex EMC Microcollection Enamine Exclusive Chemistry FCHC InovaPharm InterBioScreen | Labotest Life Chemicals Lithuania MDD World Molecules MDPI Maybridge Menai Moscow MedChem Labs Nanosyn Otava Peakdale | Pharmeks Princeton Biomolecular Pyxis Discovery Scientific Exchange Sigma-Aldrich SPECS Spectrum Info TimTec Toslab Tripos Vitas-M Lab |
| --- | --- | --- | --- |

Out of the 300 molecules profiled against the urea-based kinase inhibitors templates, 12 were included in the study to represent the selection methodology.

| Compound 1 | ChemDiv Screening collection No longer available |
| --- | --- |
| Compound 2 | ChemDiv Screening Collection Order Number: 1488-0665 |
| Compound 3 | Aurora Screening Library Order Number: K00.438.576 |
| Compound 4 | ChemDiv Screening Collection Order Number: F538-0877 |
| Compound 5 | ChemDiv Screening Collection Order Number: L907-0014 |
| Compound 6 | ChemDiv Screening Collection Order Number: G756-2381 |
| Compound 7 | ChemDiv Screening Collection Order Number: G433-0165 |
| Compound 8 | ChemDiv Screening Collection Order Number: 8012-5139 |
| Compound 9 | ChemDiv Screening Collection Order Number: D003-1056 |
| Compound 10 | ChemDiv Screening Collection Order Number: D454-0340 |
| Compound 11 | ChemDiv Screening Collection Order Number: G851-0649 |
| Compound 12 | ChemDiv Screening Collection No longer available |
