# Supplementary material for: Applying Ligands Profiling Using Multiple Extended Electron Distribution Based Field Templates and Feature Trees Similarity Searching in the Discovery of New Generation of Urea-Based Antineoplastic Kinase Inhibitors
Source: PLoS One. 2012 Nov 20;7(11):e49284. doi: 10.1371/journal.pone.0049284 (PMC3502486; doi:10.1371/journal.pone.0049284)
Supplement: Text S7 — Ftrees results of feature trees similarity against NCI database. (DOCX) [file pone.0049284.s007.docx]

**Ftrees results of feature trees similarity against NCI database:**

| Query:  O=C(Nc1ccccc1)Nc4ccc3sc(=O)n(Cc2ccc(Cl)cc2Cl)c3c4 |  | 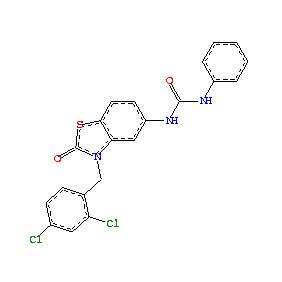 |
| --- | --- | --- |
| Rank , compound code and SMILES | Similarity | Structure |
| 1- 159268 SMILES:O=C1Nc2ccccc2c2ccccc2NC(CCCCCCCC1)=O | 0.899 | 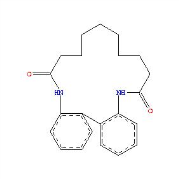 |
| 2-629694 SMILES:O=C1Nc2ccccc2c2ccccc2NC(CCCC1)=O | 0.899 | 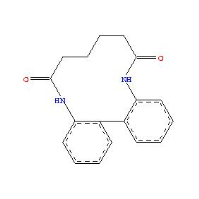 |
| 3-195294 SMILES:O=C(Nc1ccccc1)Nc1cc2oc3ccccc3c2cc1 | 0.898 | 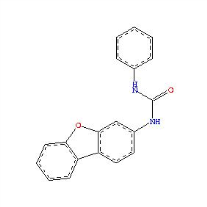 |
| 4-42065  SMILES:O=C(Nc1ccccc1OC)Nc1cccc2cc3ccccc3nc12 | 0.896 | 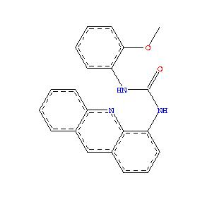 |
| 5-307235 SMILES:O=C1N2CCCCNC(CC(NCCC2c2ccccc2C=C1)=O)c1ccccc1 | 0.895 | 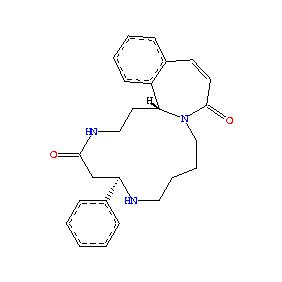 |
| 6-629693  SMILES:O=C1Nc2ccccc2c2ccccc2NC(CC1)=O | 0.892 | 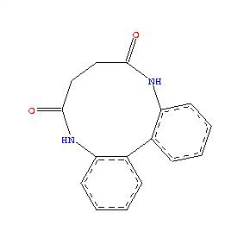 |
| 7-78710  SMILES:OC(C1=Nc2cccc3cccc(N1)c23)c1ccccc1 | 0.891 | 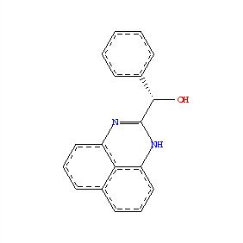 |
| 8-629698 SMILES:O=C1Nc2ccccc2c2ccccc2NC(CCCCCCCCC1)=O | 0.887 | 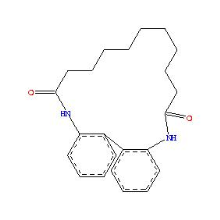 |
| 9-366466 SMILES:O=C1NCCCN2C(c3ccccc3C(C2)CCNC(C1)c1ccccc1)=O | 0.887 | 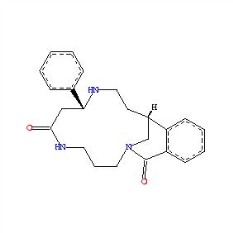 |
| 10-629695  SMILES:O=C1Nc2ccccc2c2ccccc2N(C(CCCC1)=O)CC | 0.886 | 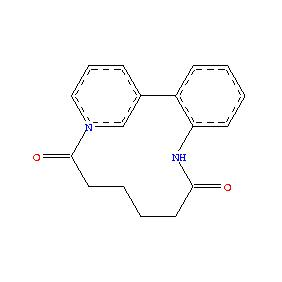 |
| 11-141298 SMILES:O=C(NCc1ccccc1)Cn1cccc(c1)C(=O)Nc1ccccc1 | 0.885 | 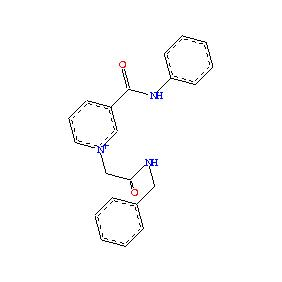 |
| 12-629696 SMILES:O=C1Nc2ccccc2c2ccccc2N(C(CC1)=O)CCCN(C)C | 0.884 | 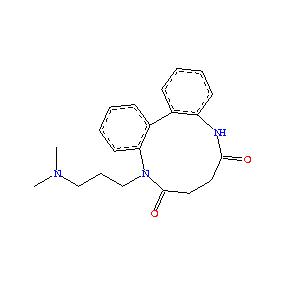 |
| 13-141275 SMILES:Clc1ccc(cc1)NC(=O)Cn1cccc(c1)C(=O)Nc1ccccc1 | 0.883 | 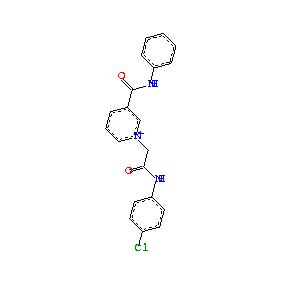 |
| 14-180807 SMILES:O=C(Nc1ccccc1)c1cc2C(N(C(c2cc1)=O)c1ccccc1)=O | 0.882 | 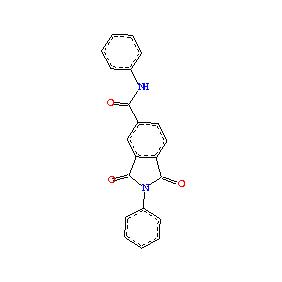 |
| 15-307140  SMILES:N1=Cc2ccccc2NCCNc2ccccc2C=NCCCC1 | 0.882 | 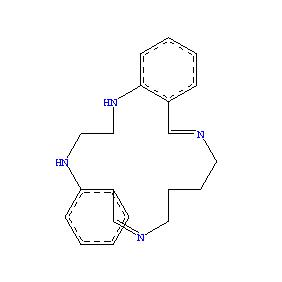 |
| 16-673156 SMILES:O=C(OC1C#CCSCC#CC=C1)Cc1nccc2c3ccccc3nc12 | 0.879 | 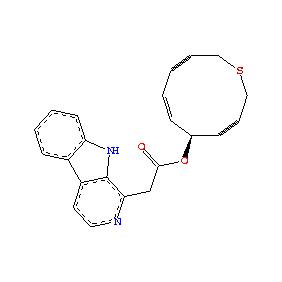 |
| 17-307139  SMILES:N1=Cc2ccccc2NCCNc2ccccc2C=NCCC1 | 0.879 | 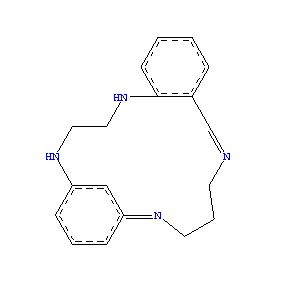 |
| 18-154693 SMILES:O=C1OC(c2cccc3cccc1c23)(c1ccccc1)c1ccccc1 | 0.879 | 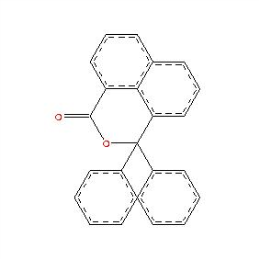 |
| 19-307138  SMILES:N1=Cc2ccccc2NCCNc2ccccc2C=NCC1 | 0.877 | 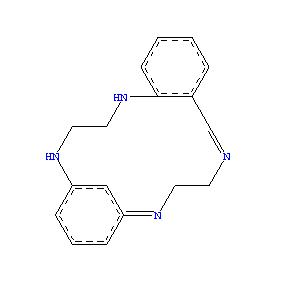 |
| 20-78793  SMILES:O=C(Nc1ncnc2n(cnc12)Cc1ccccc1)c1ccccc1 | 0.877 | 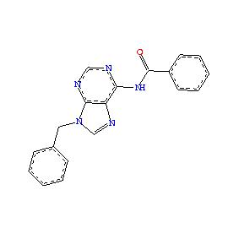 |
| 21-141297 SMILES:Clc1ccc(cc1)NC(=O)c1cccn(c1)CC(=O)NCc1ccccc1 | 0.877 | 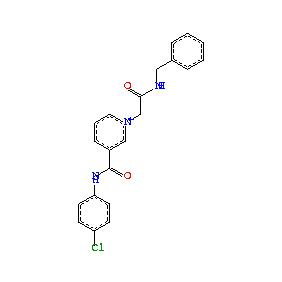 |
| 22-694166 SMILES:Clc1ccc(cc1)Oc1ccc(cc1Cl)N1C(CSC1c1ccc(cc1Cl)Cl)=O | 0.876 | 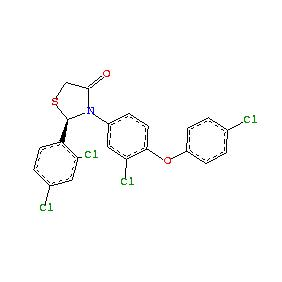 |
| 23-659102 SMILES:Clc1ccc(cc1)C=C1SC=2SC=C([N+]=2C1=O)CC(=O)Nc1ccccc1 | 0.876 | 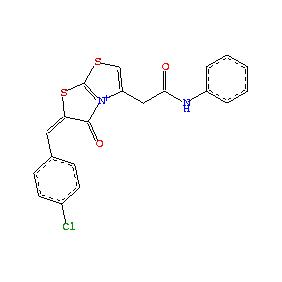 |
| 24-13980 SMILES:Clc1cccc2C(c3c(cccc3C(c12)=O)NC(=O)c1ccccc1)=O | 0.876 | 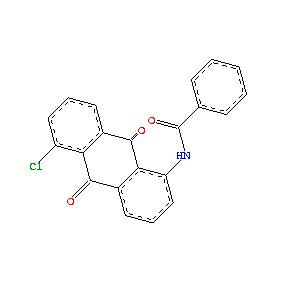 |
