# Supplementary material for: Applying Ligands Profiling Using Multiple Extended Electron Distribution Based Field Templates and Feature Trees Similarity Searching in the Discovery of New Generation of Urea-Based Antineoplastic Kinase Inhibitors
Source: PLoS One. 2012 Nov 20;7(11):e49284. doi: 10.1371/journal.pone.0049284 (PMC3502486; doi:10.1371/journal.pone.0049284)
Supplement: Text S8 — Profile of compounds (12b, 12d, 12e, 12k) on the 60 tumor cell line panel at the test dose of 10 uM. (DOCX) [file pone.0049284.s008.docx]

**Profile of compounds (12b, 12d, 12e, 12k) on the 60 tumor cell line panel at the test dose of 10 uM**


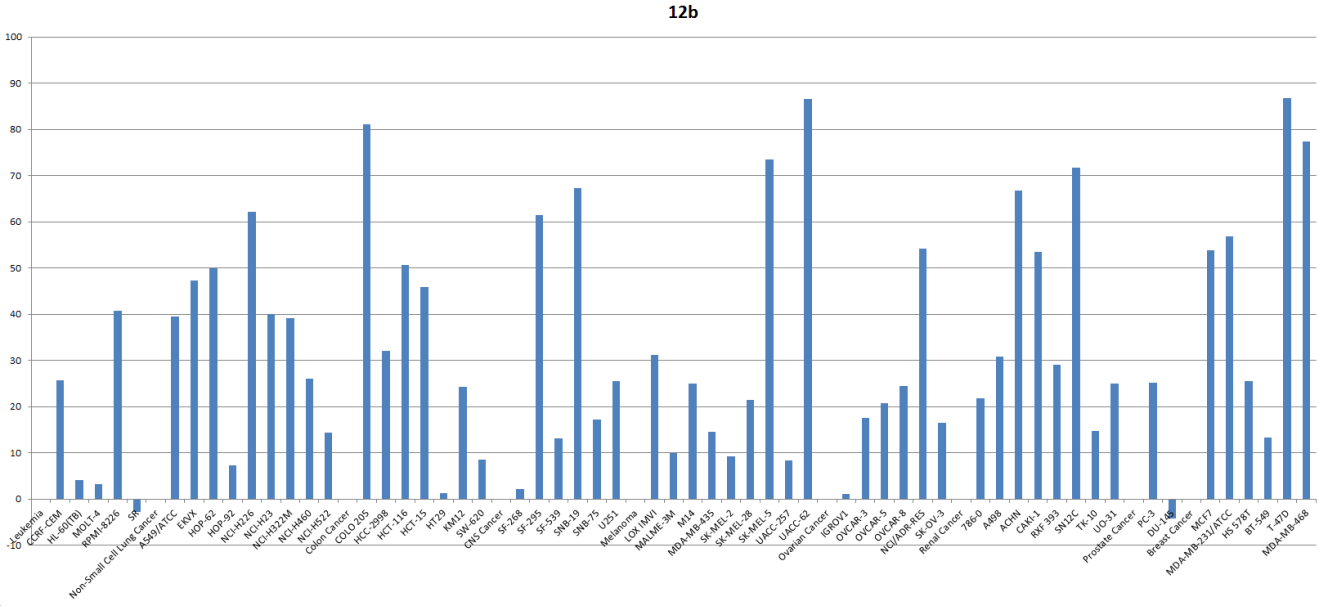


Compound **12b** shows moderate growth inhibition that didn’t exceed 100% inhibition at the test dose of 10 uM. The compound is cytostatic at 10 uM. It didn’t exceed the inhibitory limit (100% inhibition) to the lethal effect at given test dose.


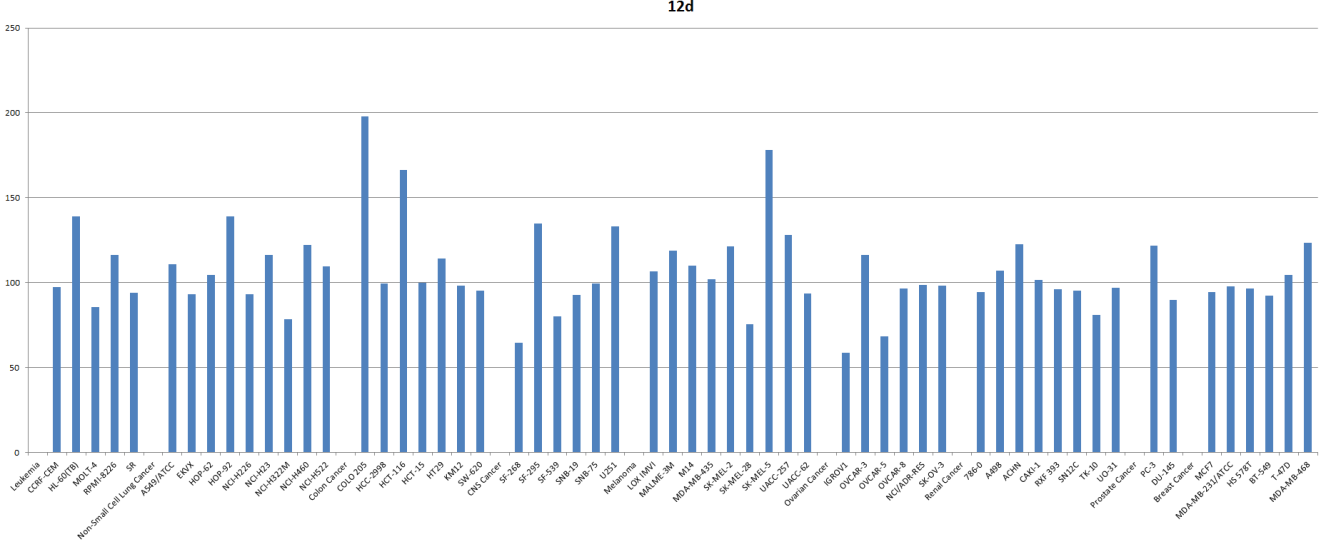


Compound **12d** showed potent growth inhibition on some tumor cell lines (e.g. some colon and melanoma cell lines).The compound effect has exceeded the inhibitory limit (100% inhibition) to the lethal effect (regression of tumor size from the original size at the beginning of the experiment) at the test dose (10 uM). The compound cytotoxicity is prominent at the test dose for some specific cell lines where it approaches the limit of 200% (100% lethality or complete tumor regression) in some of the cell lines under test at the test dose. It may indicate that this compound may have another leading mechanism that is responsible for the strong antineoplastic activity if compared to compound **12b**.


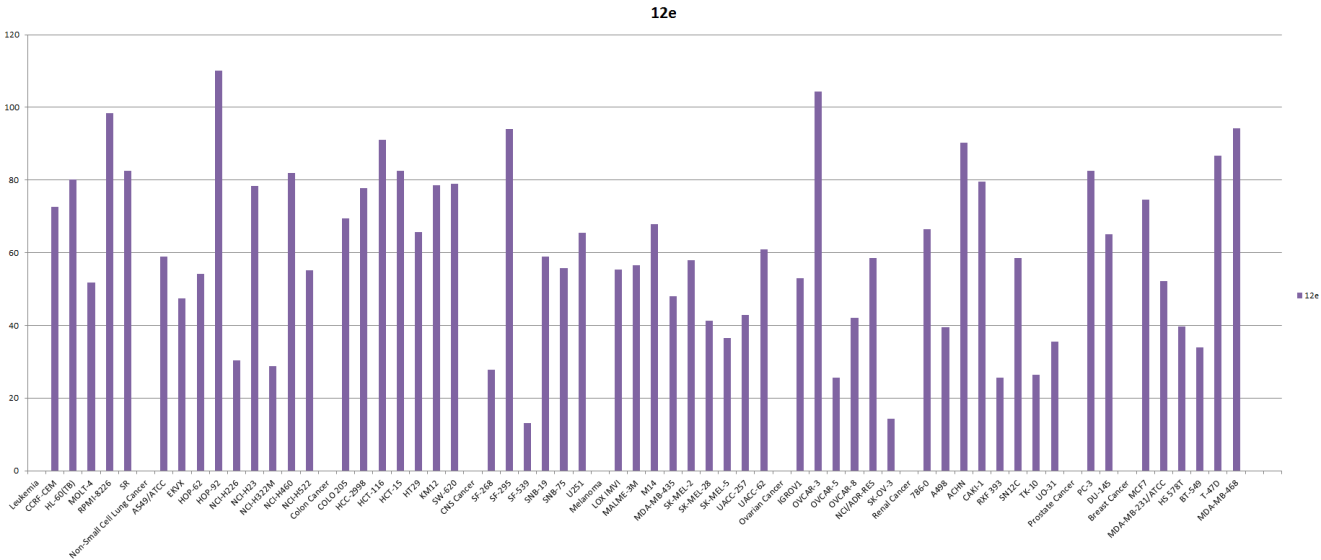


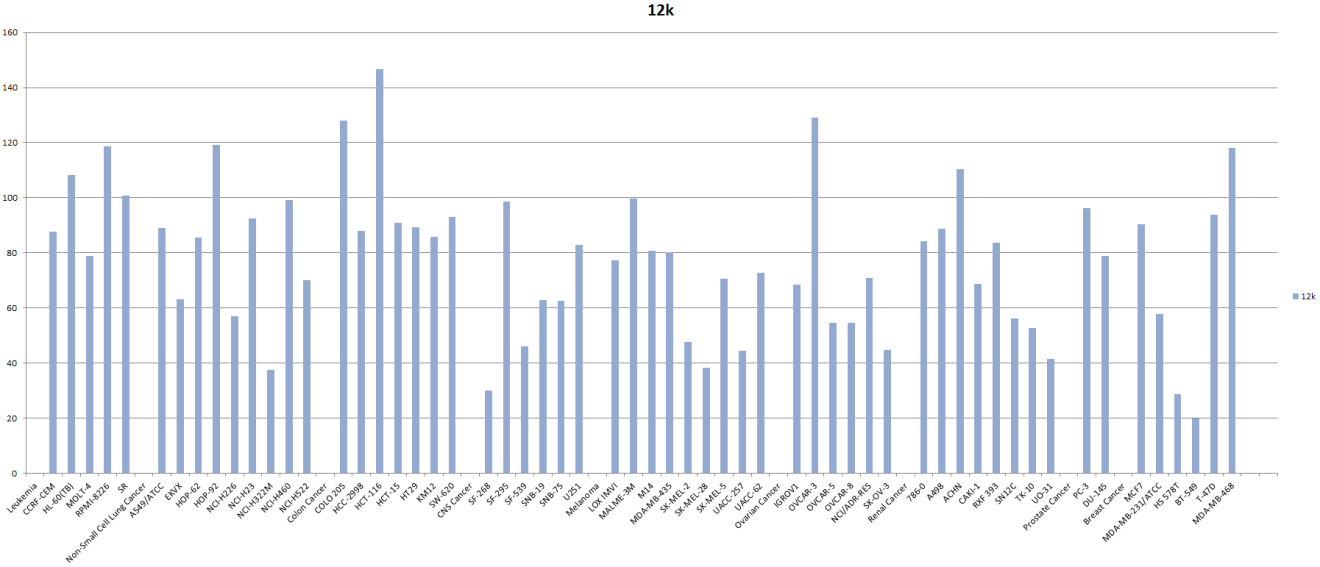


Both compounds **12e** and **12k** are showing moderate growth inhibition that exceeds the inhibitory limit (100% inhibition) to the lethal effect by about 50 % on some tumor cell lines. They didn’t approach the limit of 200% (total lethality) like **12d** but exceeds the 100% inhibition in many cell lines unlike **12b**. That is why we considered them moderate.

To get the whole picture, we illustrated the activity pattern of the four compounds :


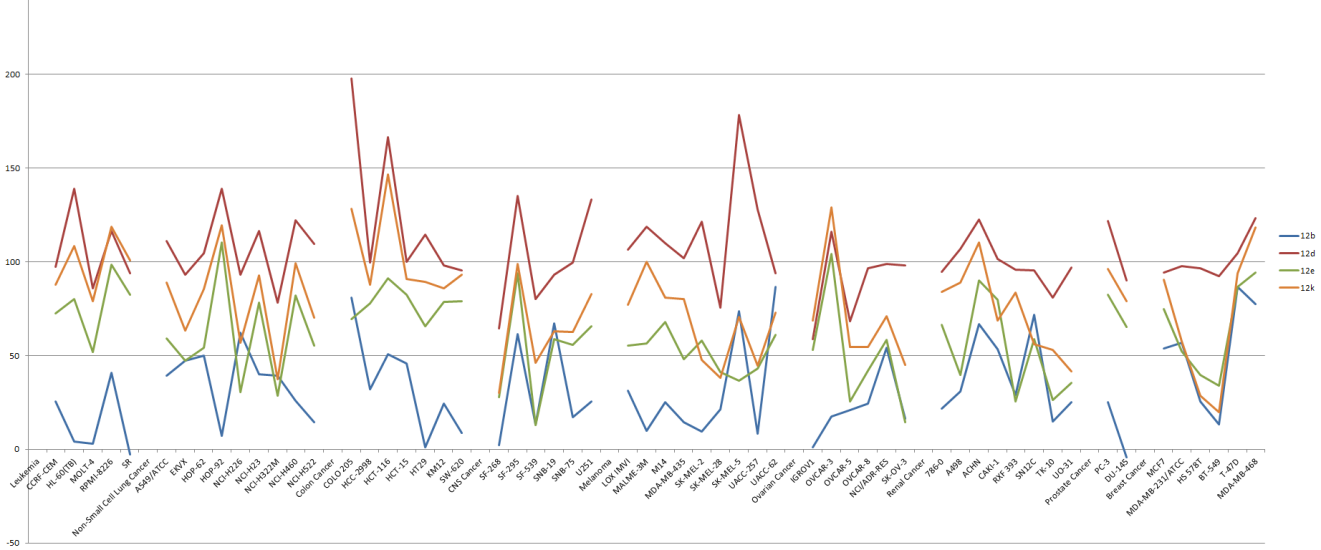


The profile of the 4 compounds shows interesting results regarding their biological pattern. It indicates that the 4 compounds belong to the same class but have variable degree of growth inhibition (**12d**>**12k**>**12e**>**12b**). This preliminary profile shows that these compounds have the same biological trend but with variable degree.
