# Supplementary material for: Applying Ligands Profiling Using Multiple Extended Electron Distribution Based Field Templates and Feature Trees Similarity Searching in the Discovery of New Generation of Urea-Based Antineoplastic Kinase Inhibitors
Source: PLoS One. 2012 Nov 20;7(11):e49284. doi: 10.1371/journal.pone.0049284 (PMC3502486; doi:10.1371/journal.pone.0049284)
Supplement: Table S1 — Kinase profiling data. (DOCX) [file pone.0049284.s009.docx]

**Kinase profiling data**

| **Kinase** | **%** | **Kinase** | **%** | **Kinase** | **%** | **Kinase** | **%** | **Kinase** | **%** |
| --- | --- | --- | --- | --- | --- | --- | --- | --- | --- |
| **MRCKa** | **-25** | **MARK1** | **1** | **HIPK1** | **47** | **NEK6** | **6** | **FMS** | **-7** |
| **ROCK1** | **-122** | **MARK3** | **-18** | **HIPK3** | **3** | **PKR** | **10** | **c-KIT** | **4** |
| **ROCK2** | **-15** | **MAPKAPK2** | **-25** | **HIPK4** | **32** | **MST1** | **-3** | **FLT3** | **2** |
| **PDK1** | **9** | **MAPKAPK3** | **-5** | **GSK3a** | **-47** | **PAK4** | **8** | **h-SRC1** | **-21** |
| **AKT1** | **-8** | **MLCK** | **-108** | **ERK1** | **-12** | **TAOK2** | **16** | **BLK** | **-15** |
| **AKT2** | **3** | **PKD2** | **25** | **p38a** | **29** | **TAOK1** | **-60** | **ITK** | **-13** |
| **PKCβII (betaII)** | **-17** | **CHK2** | **-9** | **MSSK1** | **-13** | **STK3**  **(MST2)** | **-2** | **TXK** | **-8** |
| **PKCe (epislon)** | **6** | **TSSK2** | **-12** | **GSK3b** | **1** | **MST4** | **16** | **BTK** | **-4** |
| **PKC (zeta)** | **-18** | **Camk1d** | **-33** | **JNK1** | **-28** | **CSK** | **15** | **KDR**  **(VEGFR2)** | **-12** |
| **RSK1** | **40** | **Camk1b** | **-3** | **ERK2** | **1** | **CSK** | **24** | **LYN B** | **-15** |
| **RSK3** | **-10** | **CAMK2a** | **18** | **SRPK1** | **-33** | **DDR2** | **-44** | **PDGFRB** | **19** |
| **RSK4** | **-7** | **CHK1** | **-30** | **SRPK2** | **-8** | **EPHB1** | **-33** | **PDGFRA** | **-2** |
| **DMPK** | **-44** | **AMPKA1B1G1** | **-1** | **p38b** | **-8** | **EPHB4** | **4** | **RET** | **45** |
| **MRCKb** | **-19** | **AMPKA2B1G1** | **-6** | **p38d** | **-11** | **EPHB3** | **6** | **FGR** | **-14** |
| **GRK5** | **-46** | **MYLK2** | **-9** | **p38g** | **8** | **FGFR3** | **-14** | **HCK** | **6** |
| **AKT3** | **-10** | **MYLK3** | **-43** | **AuroraA(h)** | **1** | **IGF1R** | **-11** | **BRK** | **7** |
| **AKT2** | **-19** | **MNK2** | **-9** | **CAMKK1** | **-2** | **IRR** | **-5** | **BMX** | **-45** |
| **PKC**  ** theta)** | **6** | **CAMK2D** | **-16** | **Camkk2** | **3** | **ABL2** | **-11** | **TEK** | **6** |
| **PKCδ (delta)** | **-9** | **CAMK2g** | **-22** | **PLK1** | **-12** | **ABL1 (h)** | **4** | **TRKC** | **-18** |
| **PKCh (eta)** | **4** | **CAMK2b** | **-2** | **TOPK** | **-4** | **HER2** | **-60** | **ROR2** | **-30** |
| **PKCγ (gamma)** | **-13** | **DAPK3** | **-15** | **MEKK2** | **87** | **HER4** | **-8** | **FYNA** | **67** |
| **RSK2** | **-27** | **DAPK1** | **-17** | **PAK1/CDC42** | **-1** | **EPHA1** | **-10** | **LYNA** | **6** |
| **MSK1** | **-5** | **DCAMKL2** | **-14** | **PAK3** | **1** | **EPHB2** | **-5** | **LCK** | **-11** |
| **P70S6K** | **-13** | **PIM1** | **-1** | **MINK1** | **59** | **FAK** | **9** | **SYK** | **36** |
| **PKAcα (alpha)** | **41** | **PIM2** | **9** | **PAK7**  **(PAK5)** | **46** | **PYK2 (FAK2)** | **-3** | **ZAP70** | **78** |
| **PKAcβ (beta)** | **-18** | **PKC (mu)** | **-4** | **MEK1** | **-4** | **InsR** | **-34** | **TRKA** | **-3** |
| **PKAcγ (gamma)** | **-14** | **CDK5/P25** | **38** | **MEK2** | **-22** | **JAK2** | **-12** | **TRKB** | **24** |
| **PKC (iota)** | **5** | **CDK6/CCND1** | **5** | **CK2a1** | **-18** | **MET(956 -end/H)** | **16** | **FLT1(VEGFR1)** | **28** |
| **PKC (nu)** | **-14** | **CDK6/CCND3** | **-6** | **TBK1** | **-10** | **c-MER** | **-15** | **IRAK2** | **30** |
| **PKCβI (betaI)** | **-12** | **CDK1/CCNA2(cyclinA2)** | **-33** | **NEK7** | **-1** | **TYRO3** | **16** | **ALK1** | **-6** |
| **PKCα (alpha)** | **-52** | **CDK2/CCNE1** | **-9** | **MEKK3** | **-23** | **AXL** | **-20** | **ALK2** | **1** |
| **PRKG1(PKG1)** | **-24** | **CDK7/CCNH/MNAT1** | **-5** | **ASK1** | **-9** | **EPHA2** | **9** | **IRAK4** | **-7** |
| **p70S6Kb** | **3** | **CDK3/CCNE1** | **-22** | **KHS1** | **-4** | **EPHA3** | **-3** | **B-RAF** | **-6** |
| **SGK2** | **83** | **CDK4/CCND3** | **15** | **MST3** | **-9** | **EPHA4** | **9** | **RAF1(EE)** | **-69** |
| **SGK3** | **-10** | **CLK1** | **2** | **LOK** | **-74** | **FER** | **17** | **RIPK2** | **-15** |
| **SGK1** | **14** | **CDK9/CCNK** | **4** | **MYO3b** | **18** | **FES** | **-63** | **ALK4** | **31** |
| **CAMK1** | **-13** | **CDK2/CCNA2** | **-5** | **COT** | **-133** | **FGFR1 (FLT2)** | **-20** | **TGFBR2** | **35** |
| **Camk1g** | **-27** | **CDK4/CCND1** | **-13** | **AURORA C** | **-60** | **RON** | **21** | **PI3KCB/p85a** | **6** |
| **Camk4** | **19** | **CLK2** | **37** | **AuroraB** | **-18** | **MET** | **29** | **PI3KCA/p85a** | **8** |
| **PASK** | **-21** | **CLK3** | **-18** | **NEK2** | **-14** | **MUSK** | **-11** |  |  |

**Color code:**

**Red: AGC Green: CAMK orange: CMGC blue: STE Violet: TK brown: TKL**
