# Supplementary figures and images for: Applying Ligands Profiling Using Multiple Extended Electron Distribution Based Field Templates and Feature Trees Similarity Searching in the Discovery of New Generation of Urea-Based Antineoplastic Kinase Inhibitors
Source: PLoS One. 2012 Nov 20;7(11):e49284. doi: 10.1371/journal.pone.0049284 (PMC3502486; doi:10.1371/journal.pone.0049284)

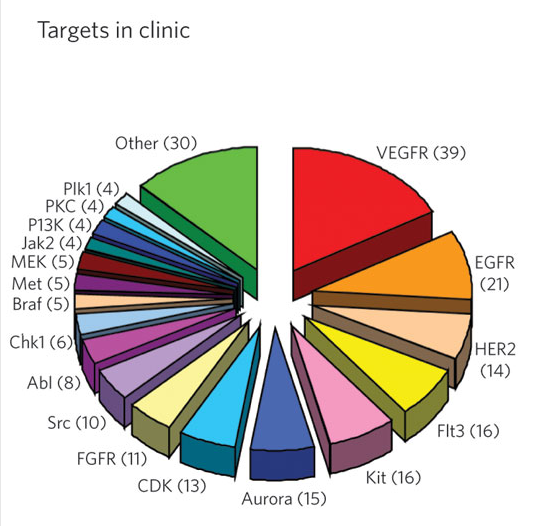

Supplement: Figure S1 — Clinically validated cancer kinome. (TIF) [file pone.0049284.s010.tif]

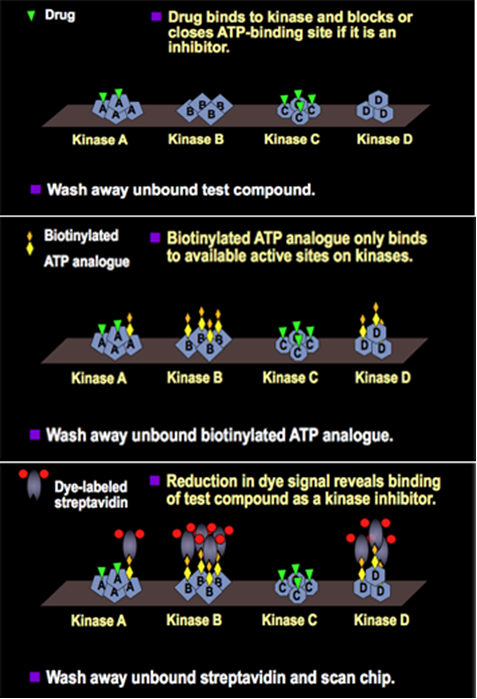

Supplement: Figure S2 — Schematic depiction of Protein Kinase Microarray-based small molecule inhibitor profiling platform. (TIF) [file pone.0049284.s011.tif]
