# Supplementary material for: Applying Ligands Profiling Using Multiple Extended Electron Distribution Based Field Templates and Feature Trees Similarity Searching in the Discovery of New Generation of Urea-Based Antineoplastic Kinase Inhibitors
Source: PLoS One. 2012 Nov 20;7(11):e49284. doi: 10.1371/journal.pone.0049284 (PMC3502486; doi:10.1371/journal.pone.0049284)
Supplement: File S1 — Pymol session files of the retrieved urea-based kinase inhibitors complexes. (ZIP) [file pone.0049284.s012.zip › Pymol session files for urea derivatives kinase complexes.pdf]

Pymol session files for urea derivatives kinases complexes

Part 1: click here

Part 2: click here
